# Supplementary material for: Structure–Activity Relationships of the Antimalarial Agent Artemisinin 10. Synthesis and Antimalarial Activity of Enantiomers of rac-5β-Hydroxy-d-Secoartemisinin and Analogs: Implications Regarding the Mechanism of Action
Source: Molecules. 2021 Jul 8;26(14):4163. doi: 10.3390/molecules26144163 (PMC8304634; doi:10.3390/molecules26144163)
Supplement: Supplementary file 1 [file molecules-26-04163-s001.zip › molecules-1229378-SM.pdf]

*Supporting Information*

# Structure-Activity Relationships of the Antimalarial Agent Artemisinin 10. Synthesis and Antimalarial Activity of Enantiomers of *rac*-5β-Hydroxy-D-Secoartemisinin and Analogs: Implications Regarding the Mechanism of Action.

**Mohamed Jahan[1], Francisco Leon[2], Frank Fronczek[3], Khaled M. Elokely[4,5], John Rimoldi[1], Shabana Khan[6] and Mitchell A. Avery[1,*]**

1. Department of BioMolecular Sciences, Division Medicinal Chemistry, School of Pharmacy, P.O. Box 1848, University of Mississippi, University, MS 38677-1848; mavery@olemiss.edu; jahan@olemiss.edu; jrimoldi@olemiss.edu
2. Department of Drug Discovery and Biomedical Sciences, University of South Carolina, Columbia SC, 29208; JLEON@mailbox.sc.edu
3. Department of Chemistry, Louisiana State University, Baton Rouge, LA. 70803-1804; ffroncz@lsu.edu
4. Department of Pharmaceutical Chemistry, Tanta University, 31527 Tanta, Egypt.
5. Institute for Computational Molecular Science and Department of Chemistry, Temple University, Philadelphia, Pennsylvania 19122, United States; kelokely@temple.edu
6. National Center for Natural Product Research, University of Mississippi, University, MS 38677; skhan@olemiss.edu

*Correspondence: mavery@olemiss.edu; mavery446@gmail.com; Tel.: 662-816-3315

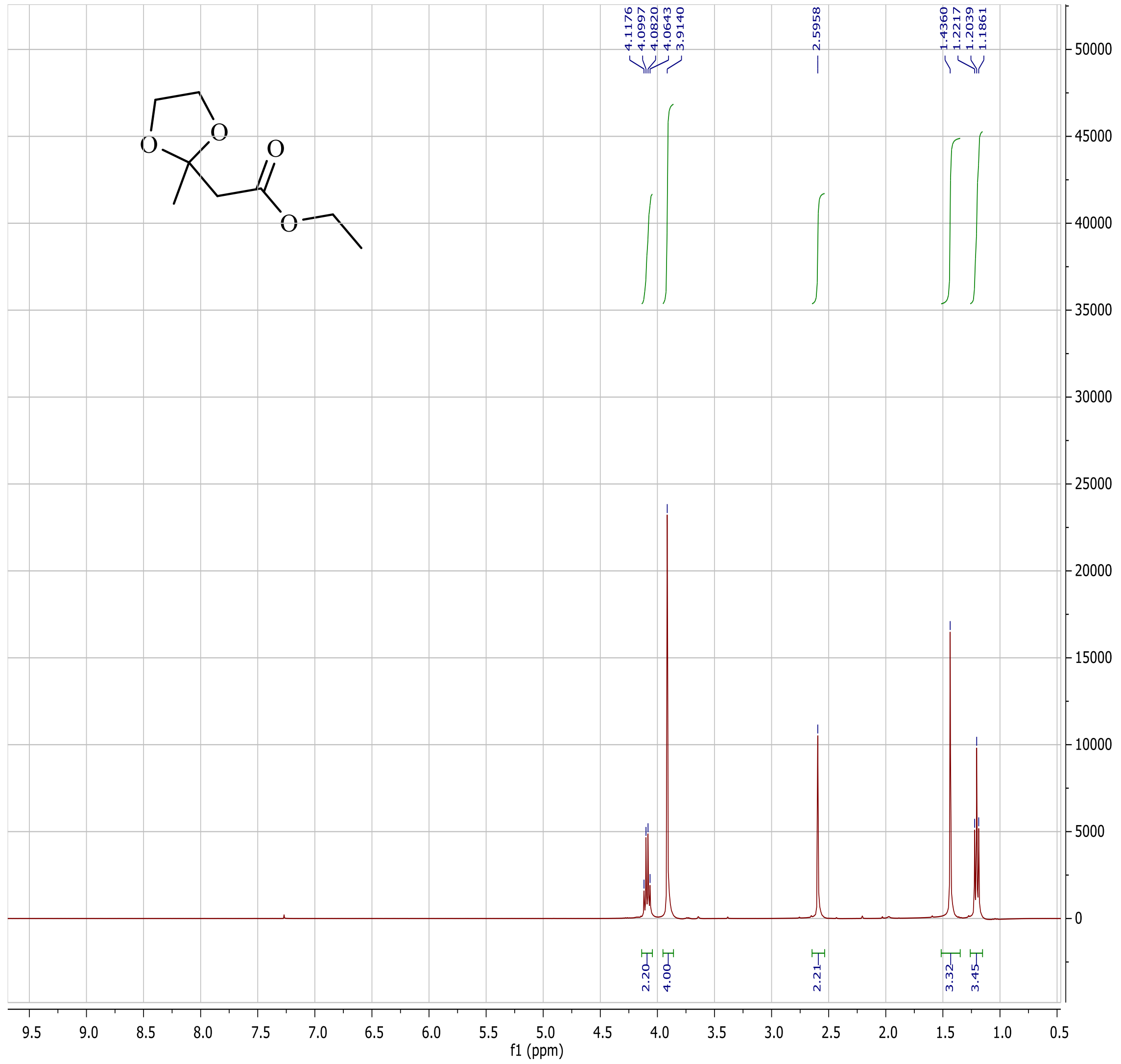


Figure S1. $^{1}$H-spectrum of the compound **3** (CDCl3, 400 MHz).

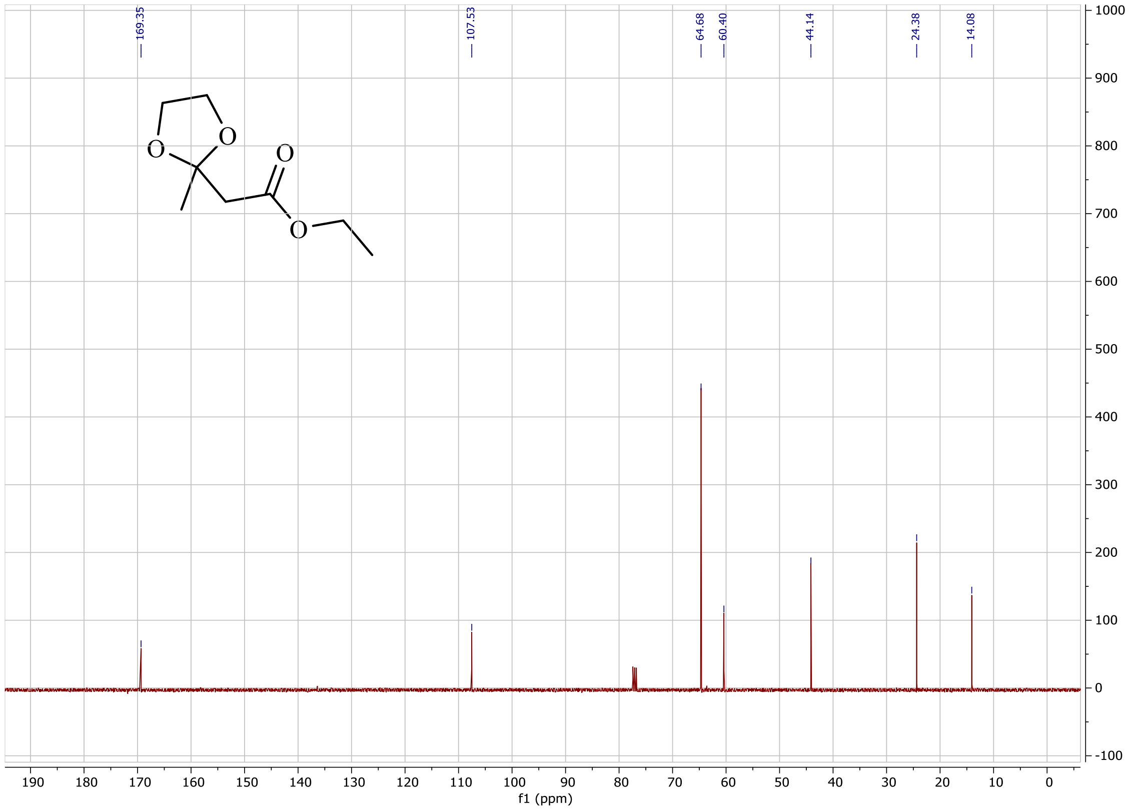


Figure S2. $^{13}C$-NMR spectrum of the compound **3** (CDCl3, 100 MHz).

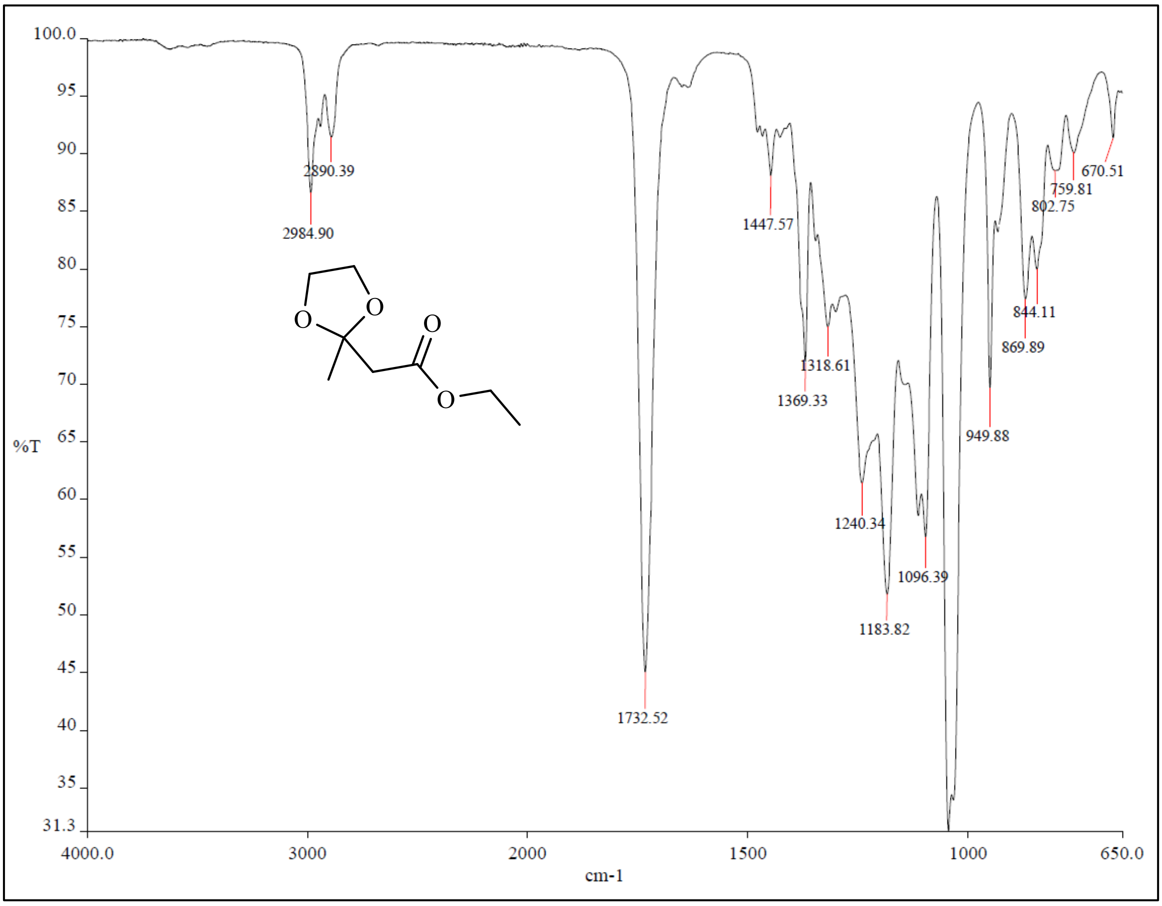


Figure S3. IR-spectrum of the compound **3**.

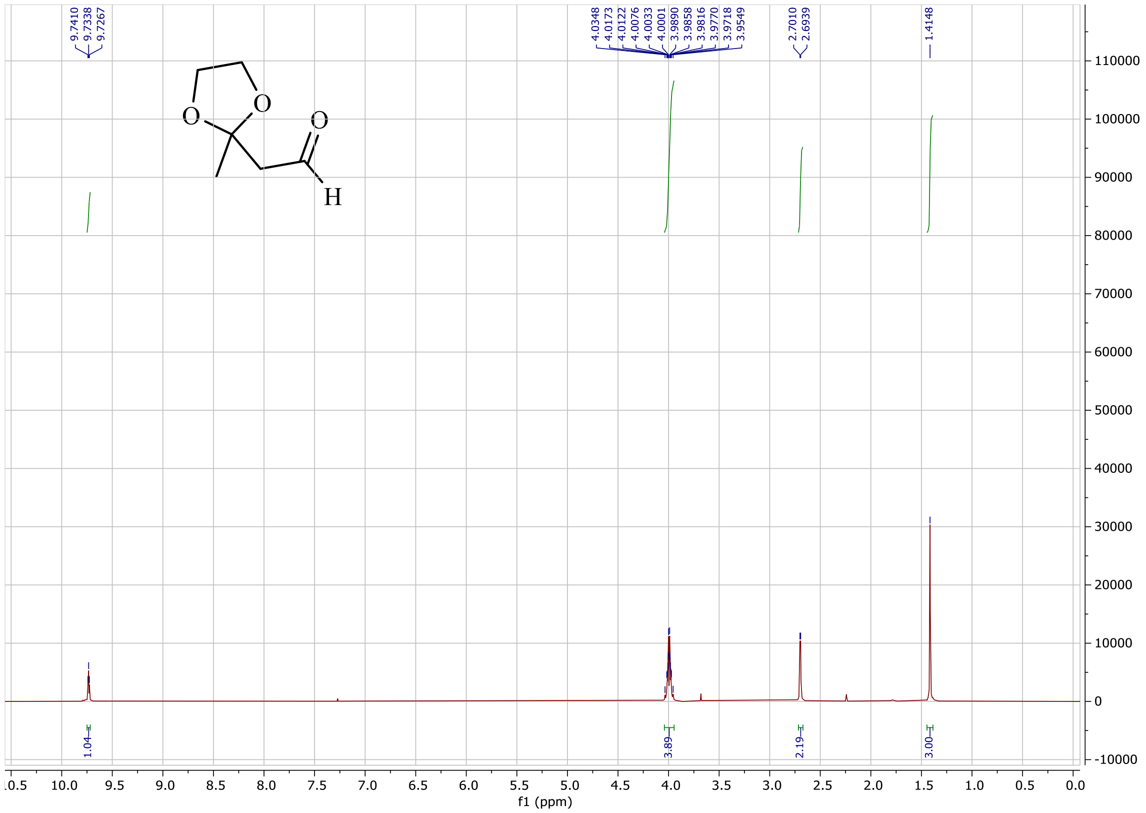


Figure S4. ¹H-spectrum of the compound **4** (CDCl3, 400 MHz).

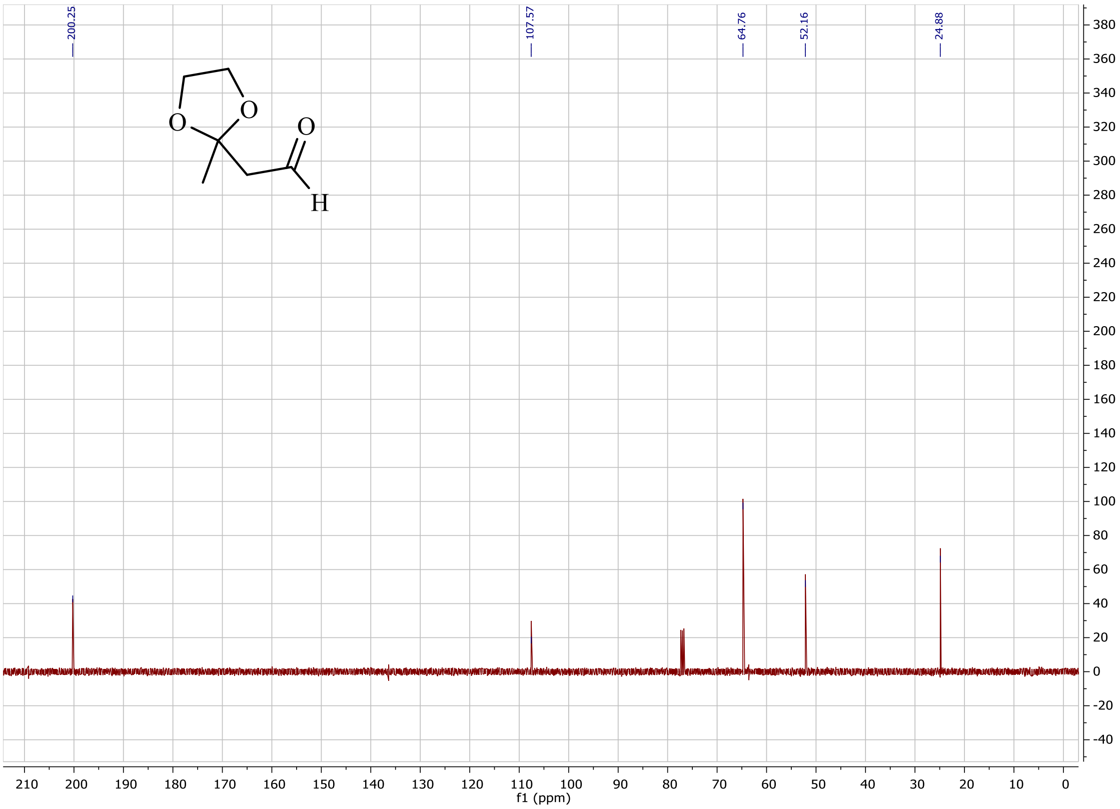


Figure S5. $^{13}$C-NMR spectrum of the compound **4** (CDCl3, 100 MHz).

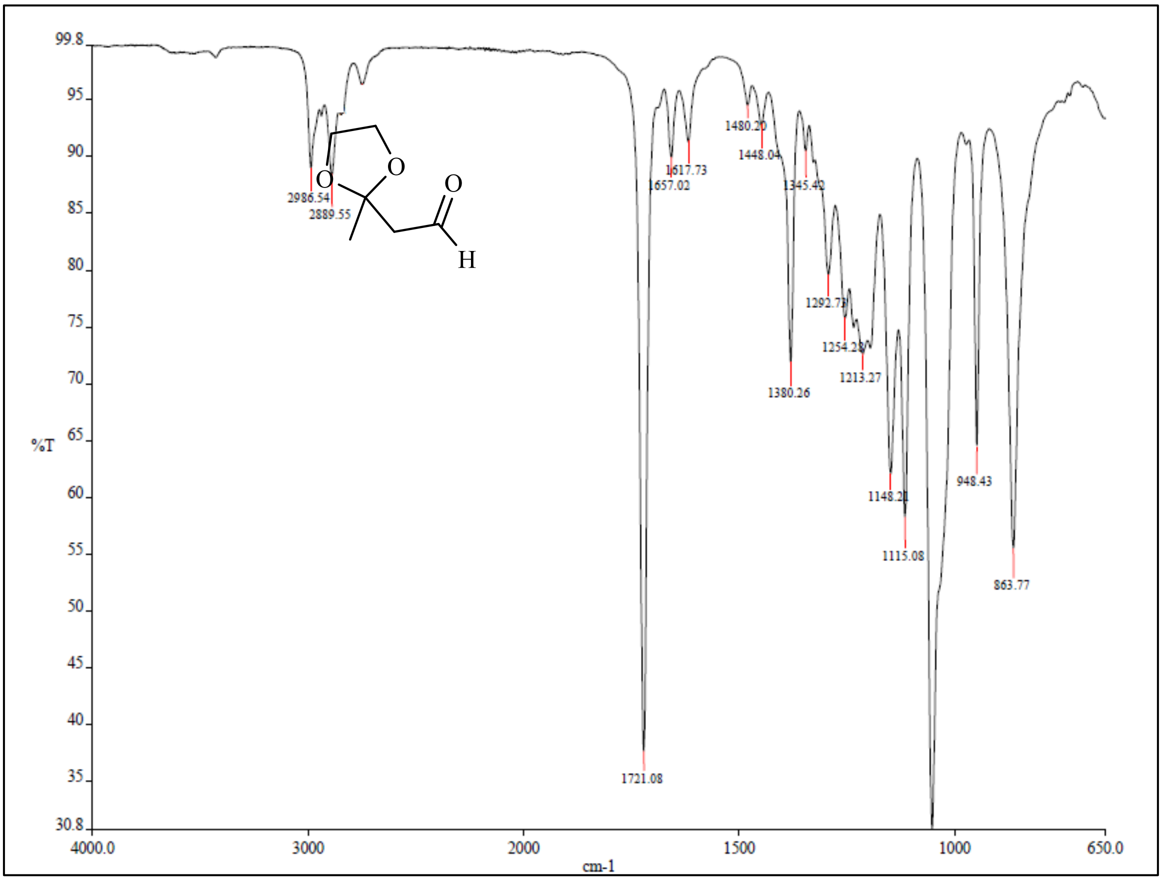


Figure S6. IR-spectrum of the compound **4**.

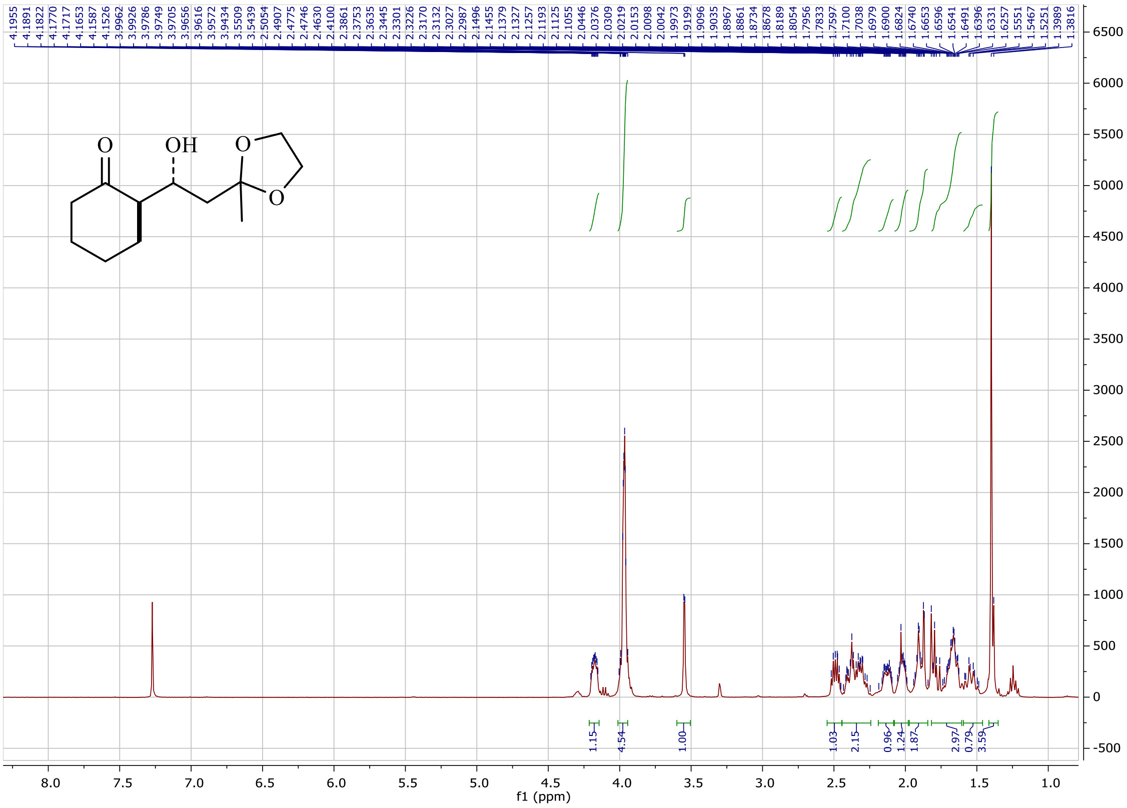

Figure S7. $^{1}H$-spectrum of the compound **5** ($CDCl_3$, 400 MHz).

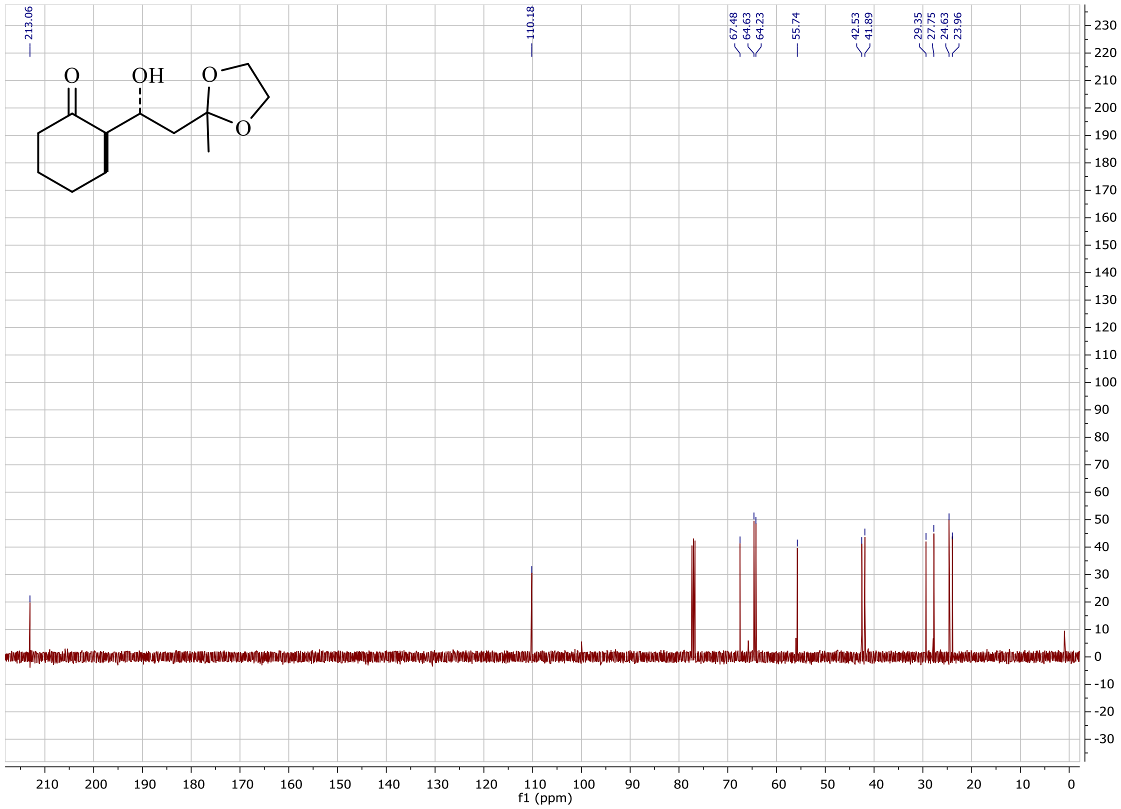


Figure S8. $^{13}$C-NMR spectrum of the compound **5** (CDCl3, 100 MHz).

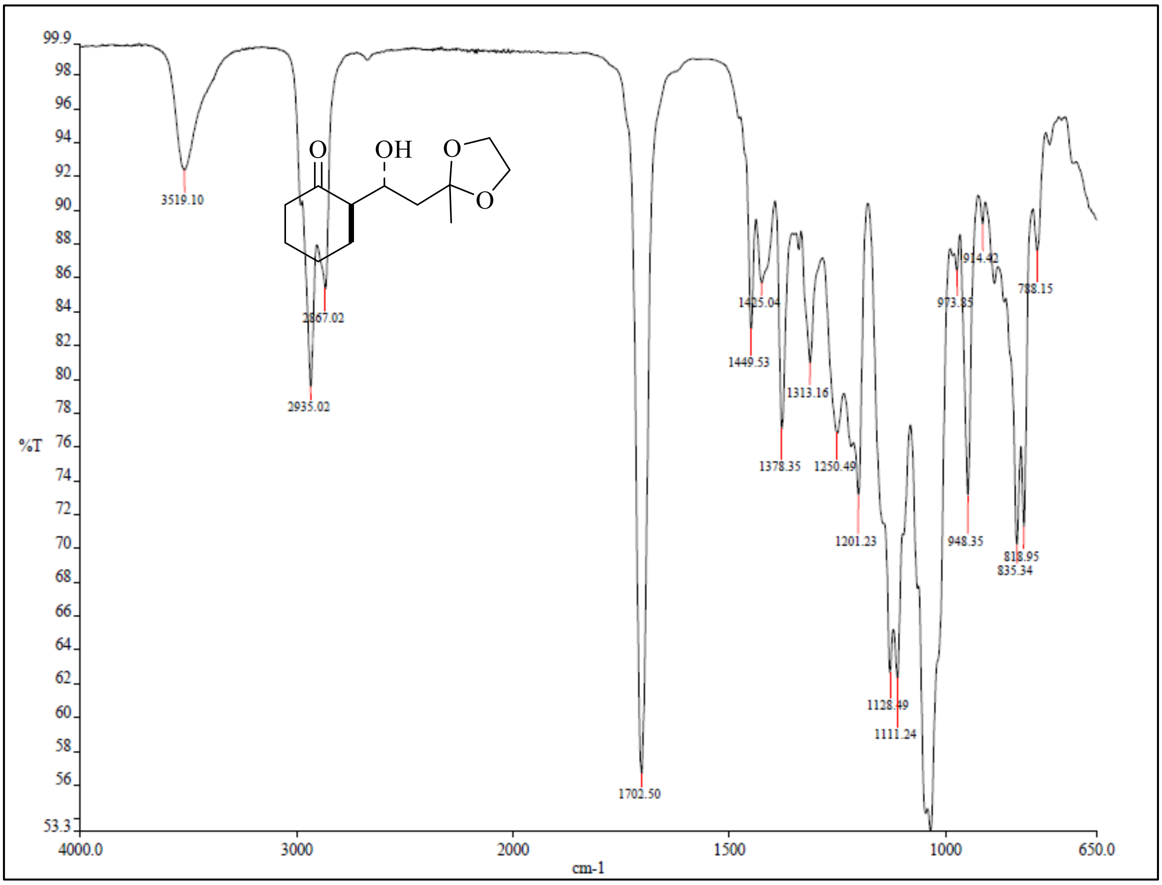


Figure S9. IR-spectrum of the compound **5**.

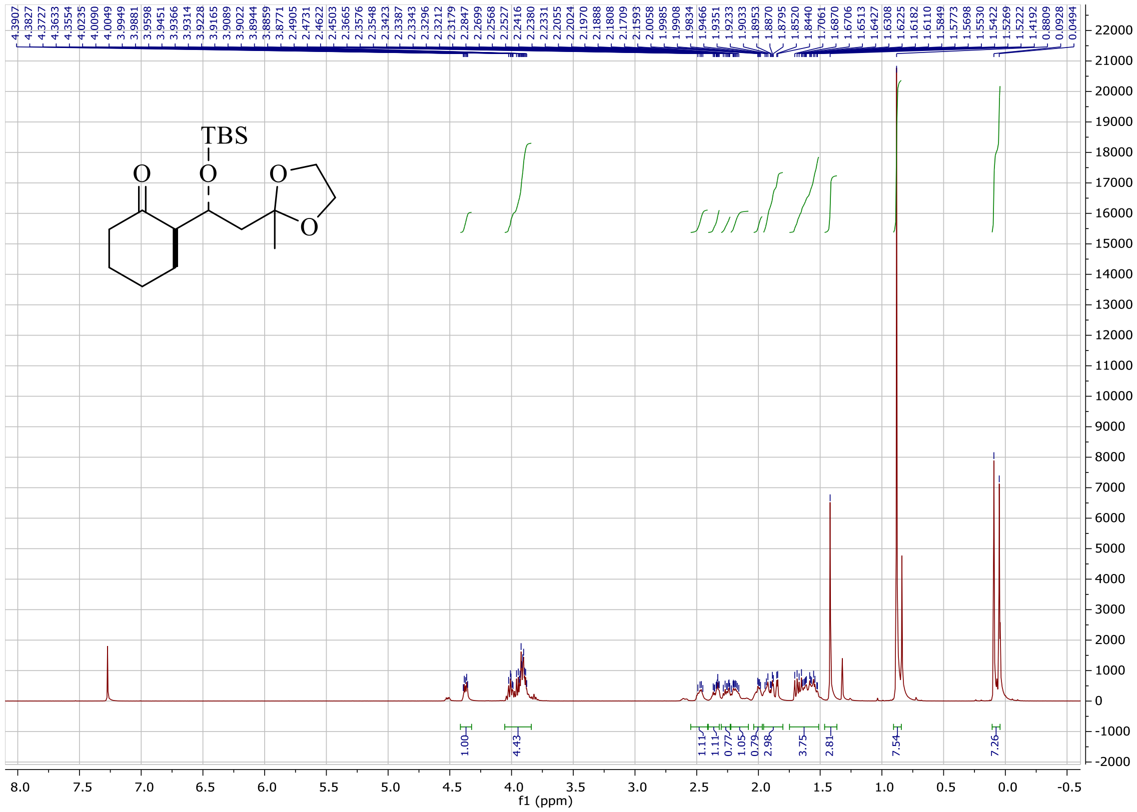

Figure S10. ¹H-spectrum of the compound **6** (CDCl3, 400 MHz).

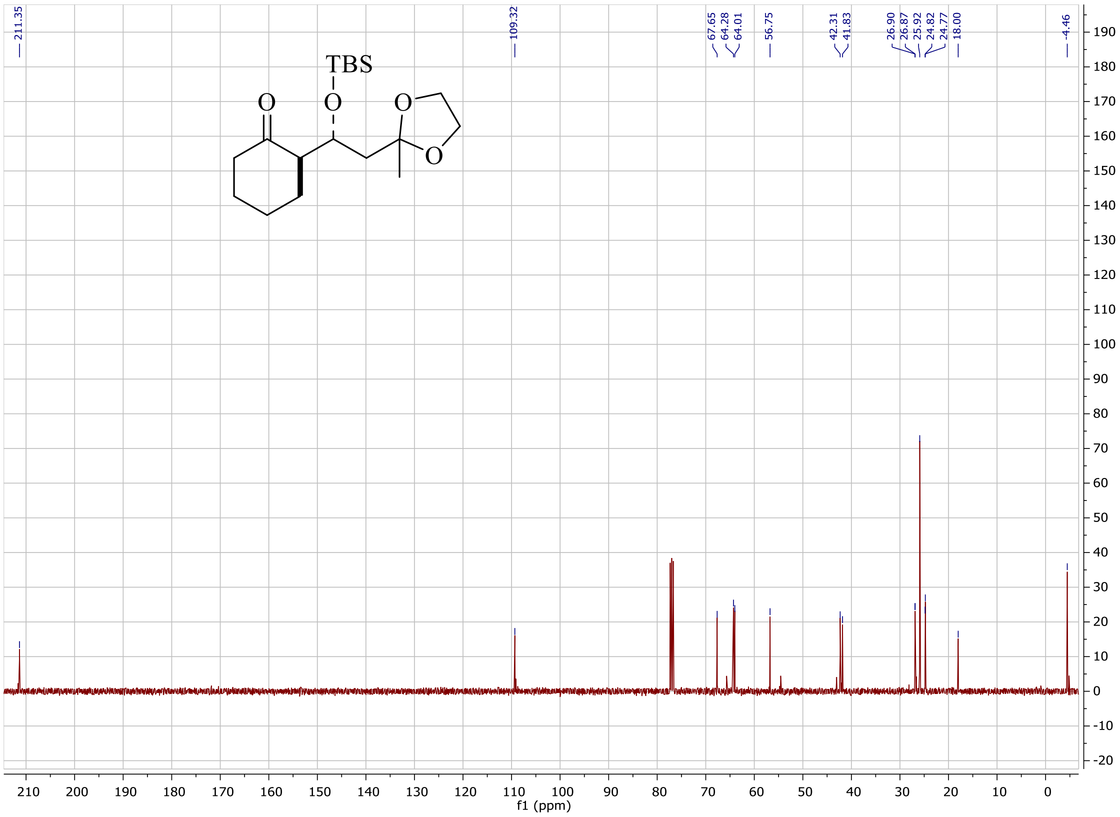

Figure S11.$^{13}$C-NMR spectrum of the compound **6** (CDCl3, 100 MHz).

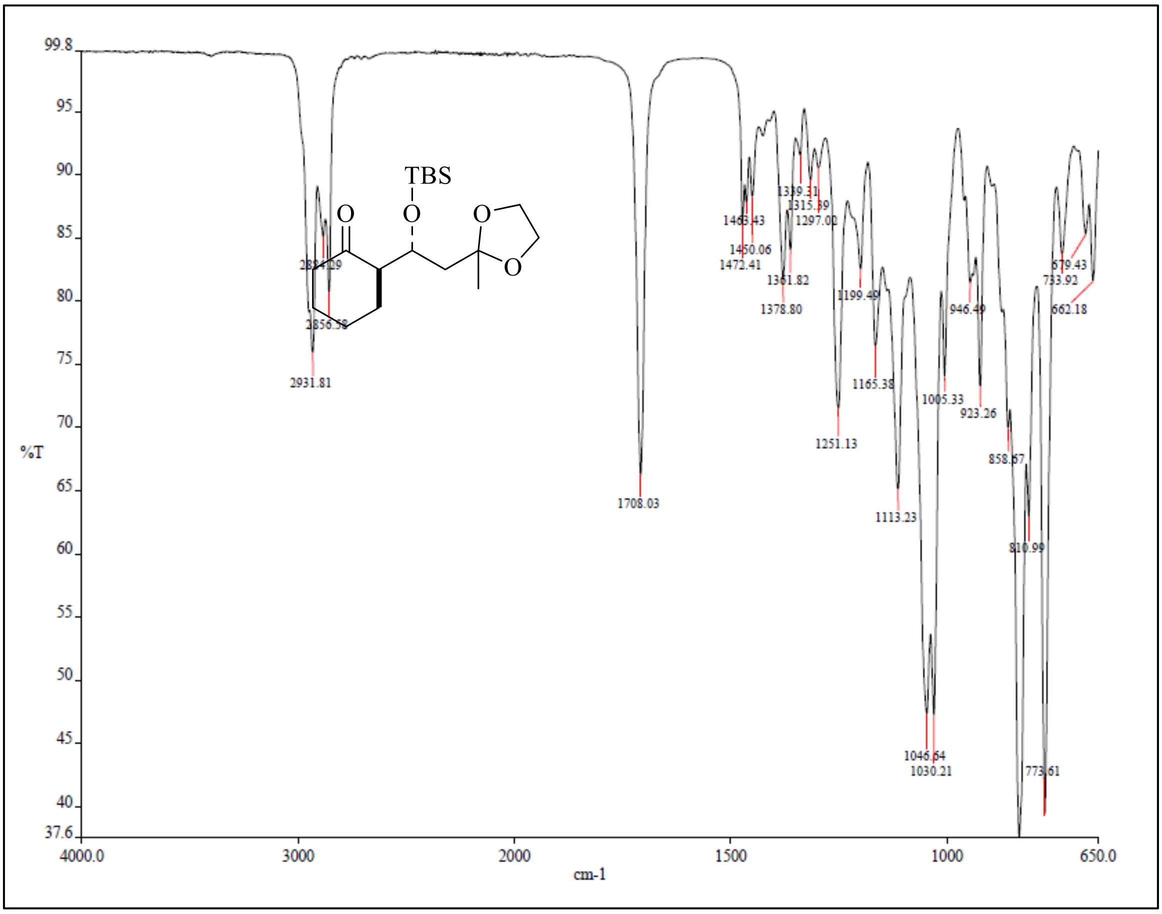


Figure S12. IR-spectrum of the compound **6**.

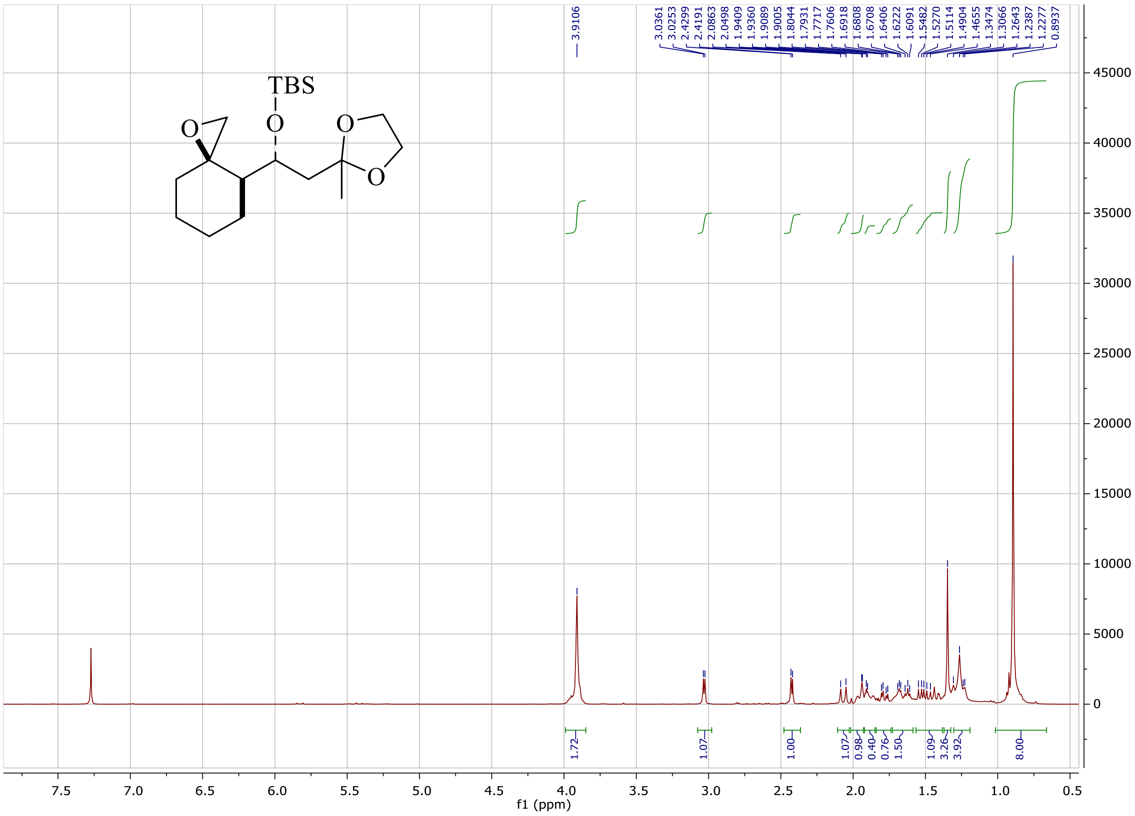

Figure S13. ¹H-spectrum of the compound **7** (CDCl3, 400 MHz).

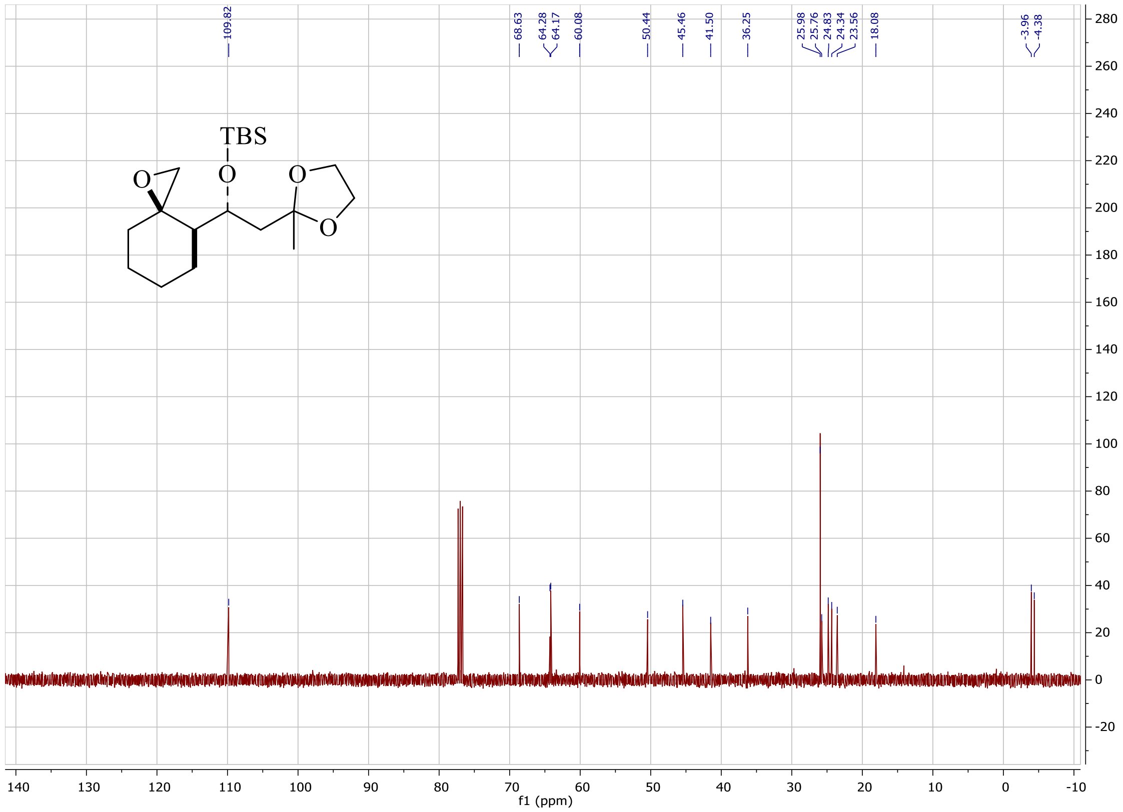


Figure S14. $^{13}$C-NMR spectrum of the compound **7** (CDCl3, 100 MHz).

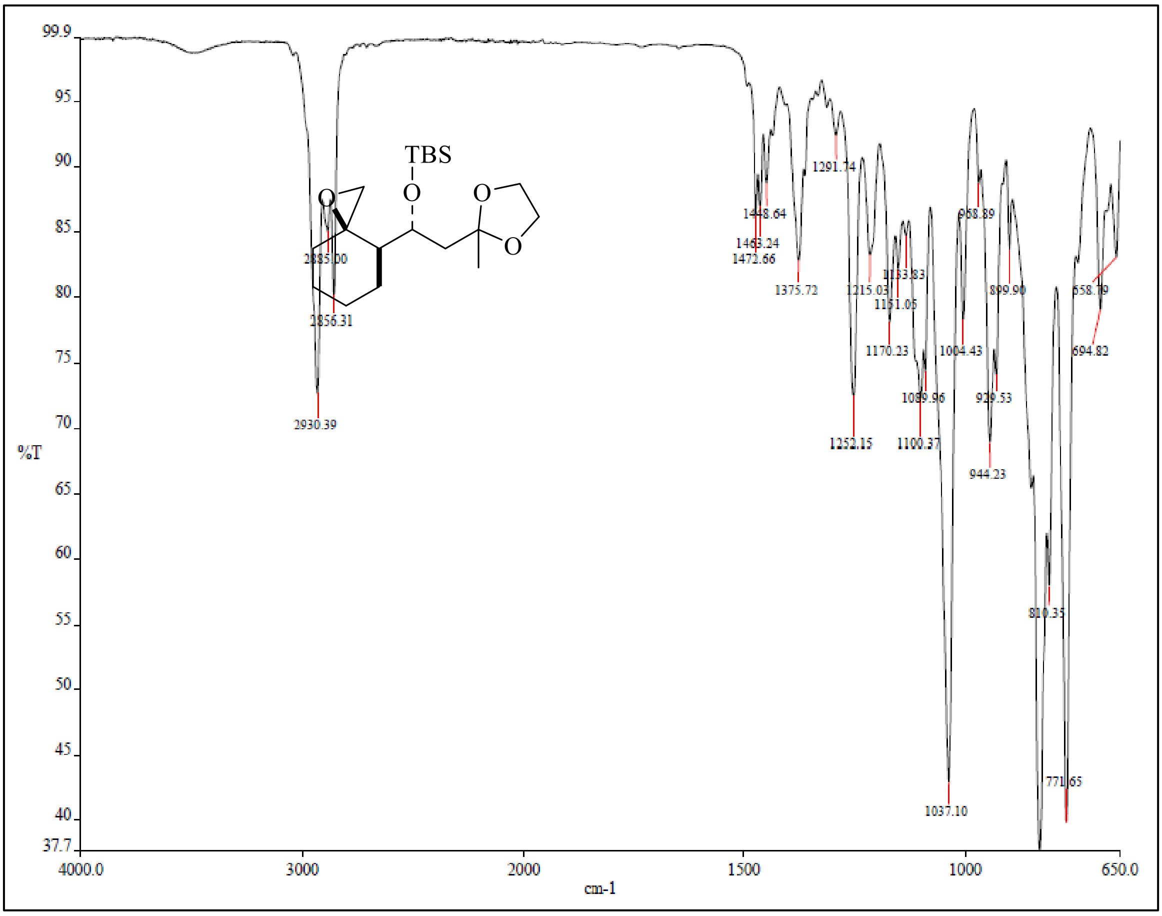


Figure S15. IR-spectrum of the compound **7**.

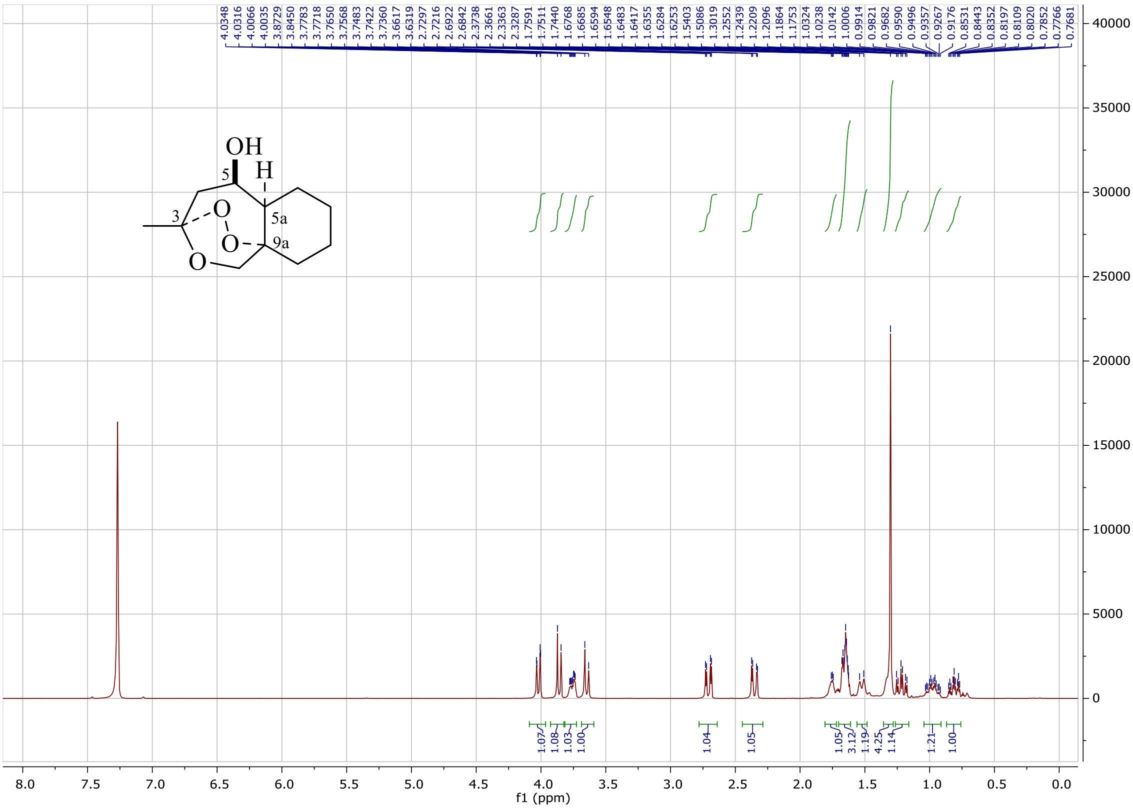

Figure S16. $^{1}$H-spectrum of the compound *rac*-**2** (CDCl3, 400 MHz).

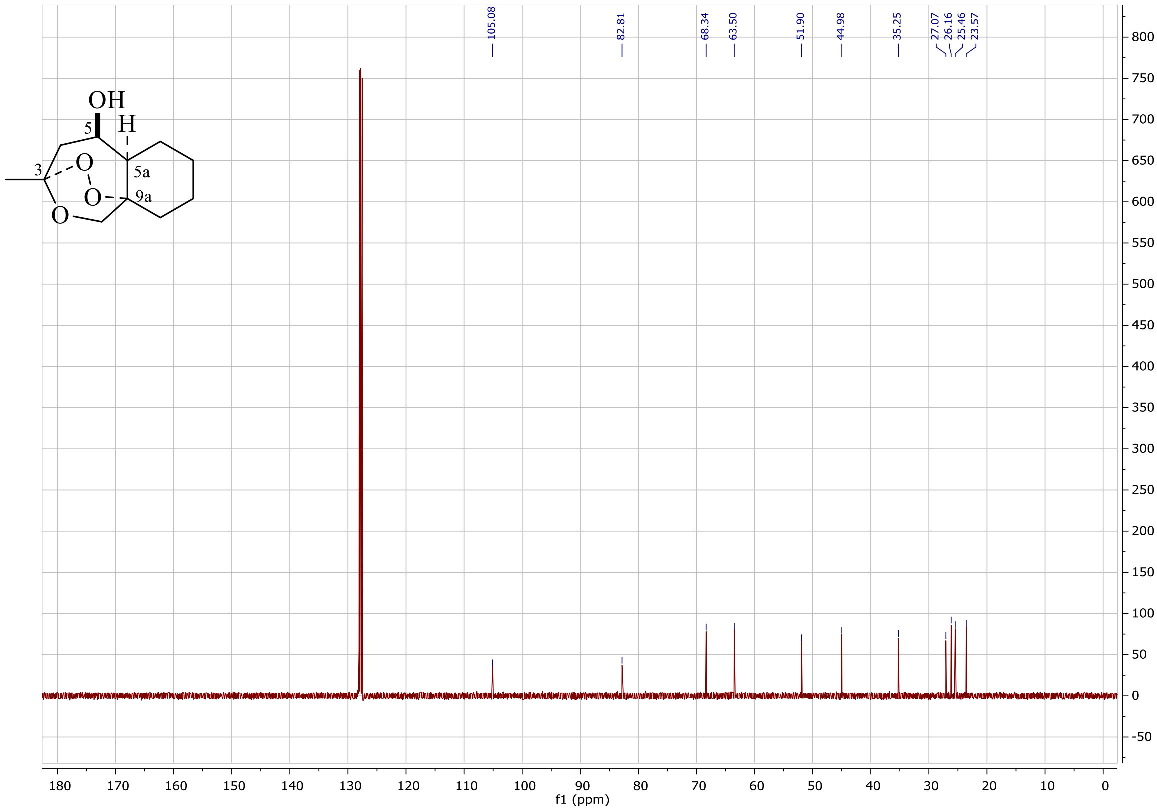


Figure S17. $^{13}$C-NMR spectrum of the compound *rac*-**2** (CDCl3, 100 MHz).

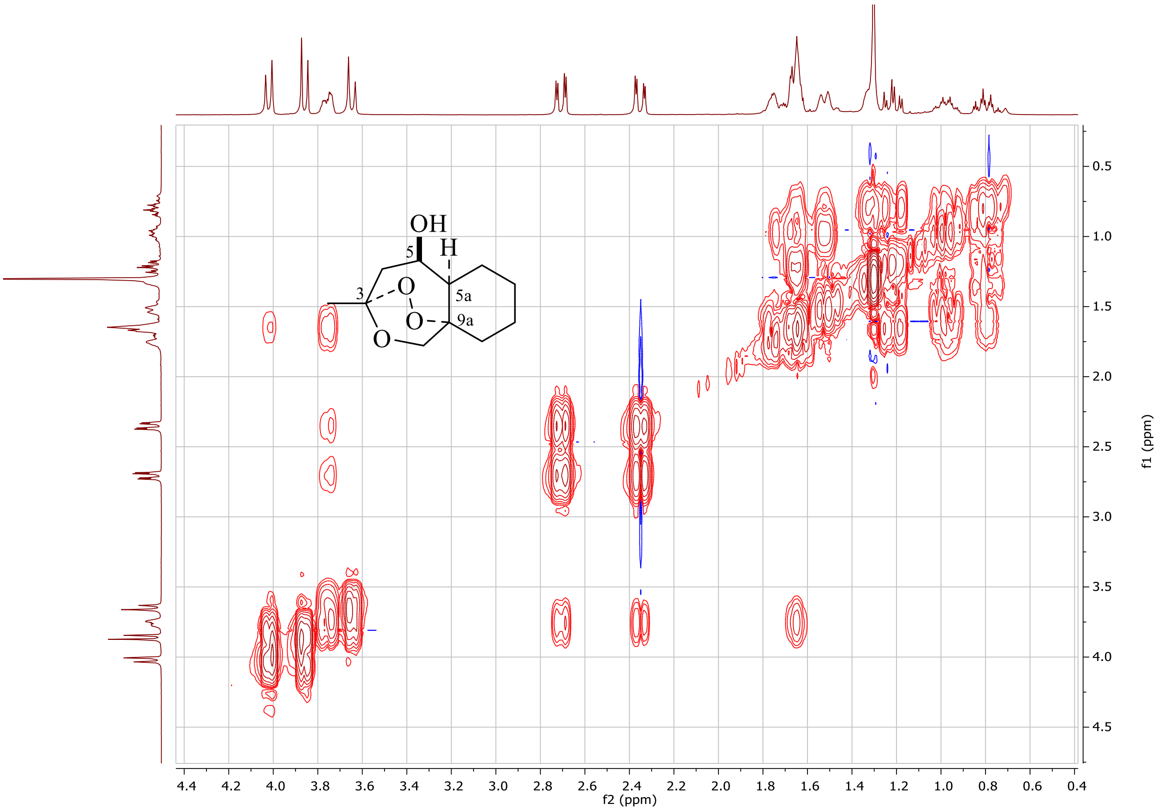


Figure S18. COSY spectrum of the compound *rac*-**2**

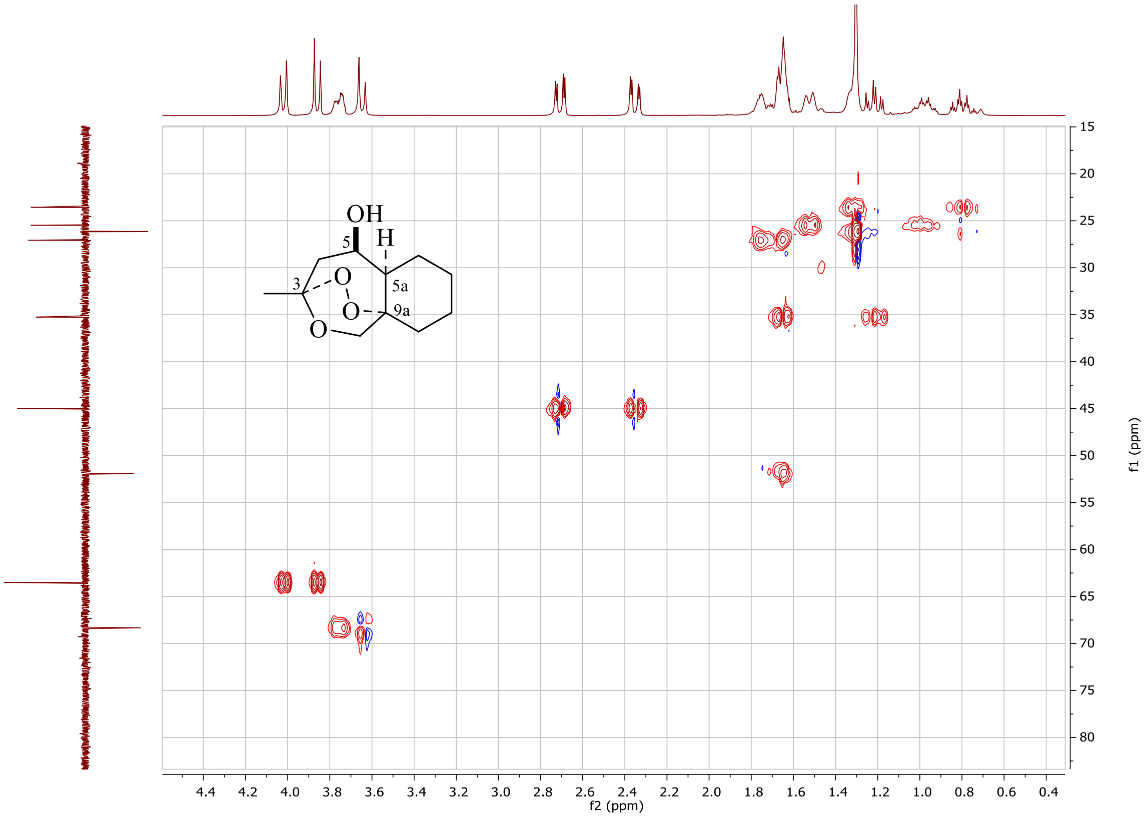


Figure S18. HSQC spectrum of the compound *rac*-**2**

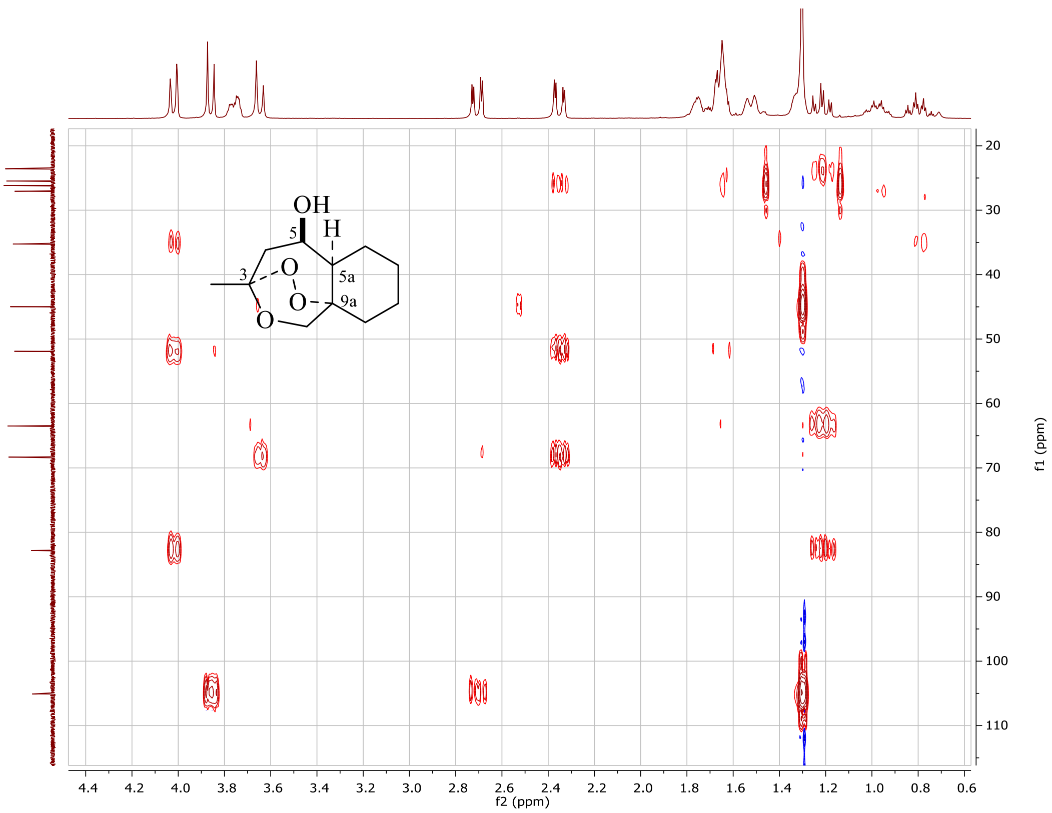


Figure S19. HMBC spectrum of the compound *rac*-**2**

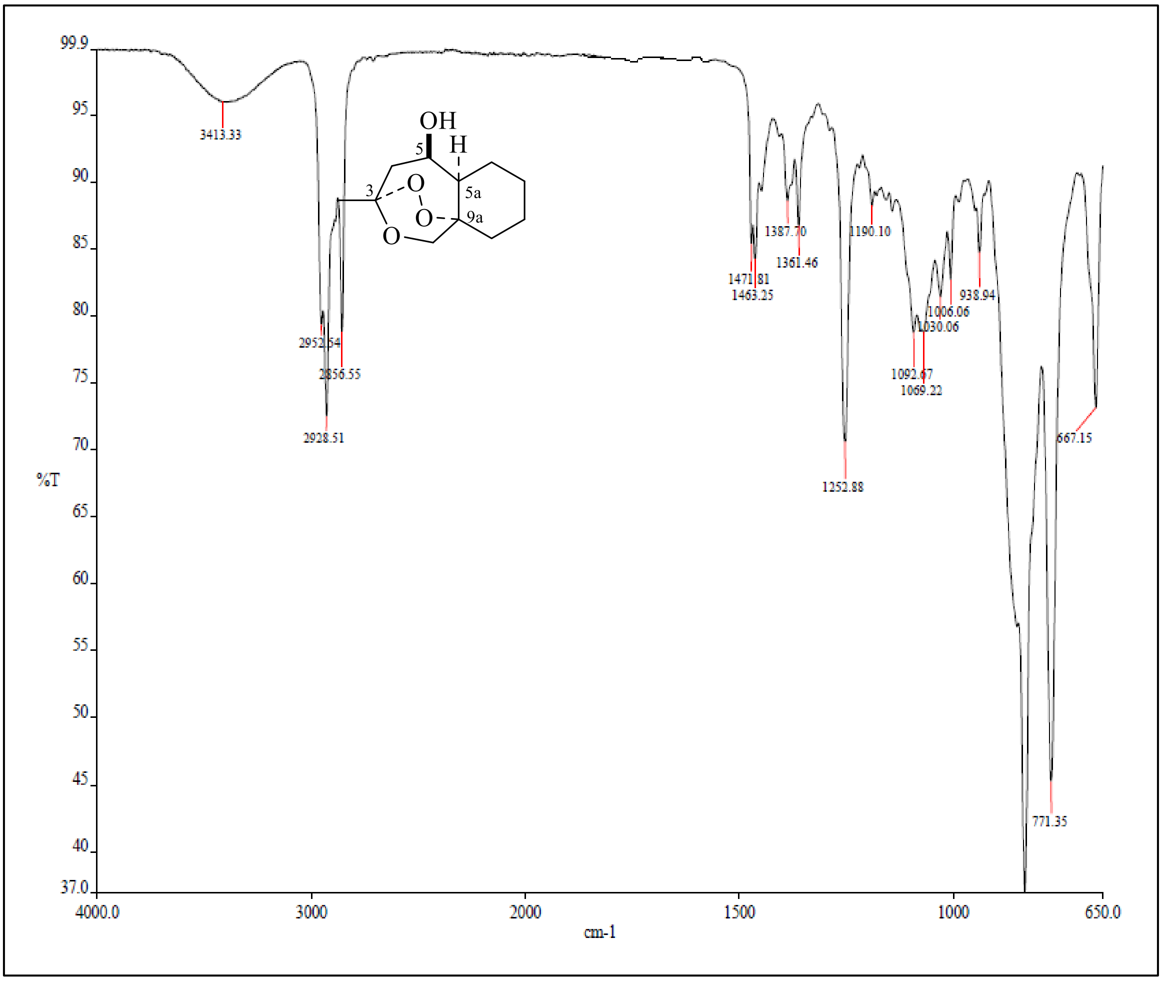


Figure S20. IR-spectrum of the compound *rac*-**2**

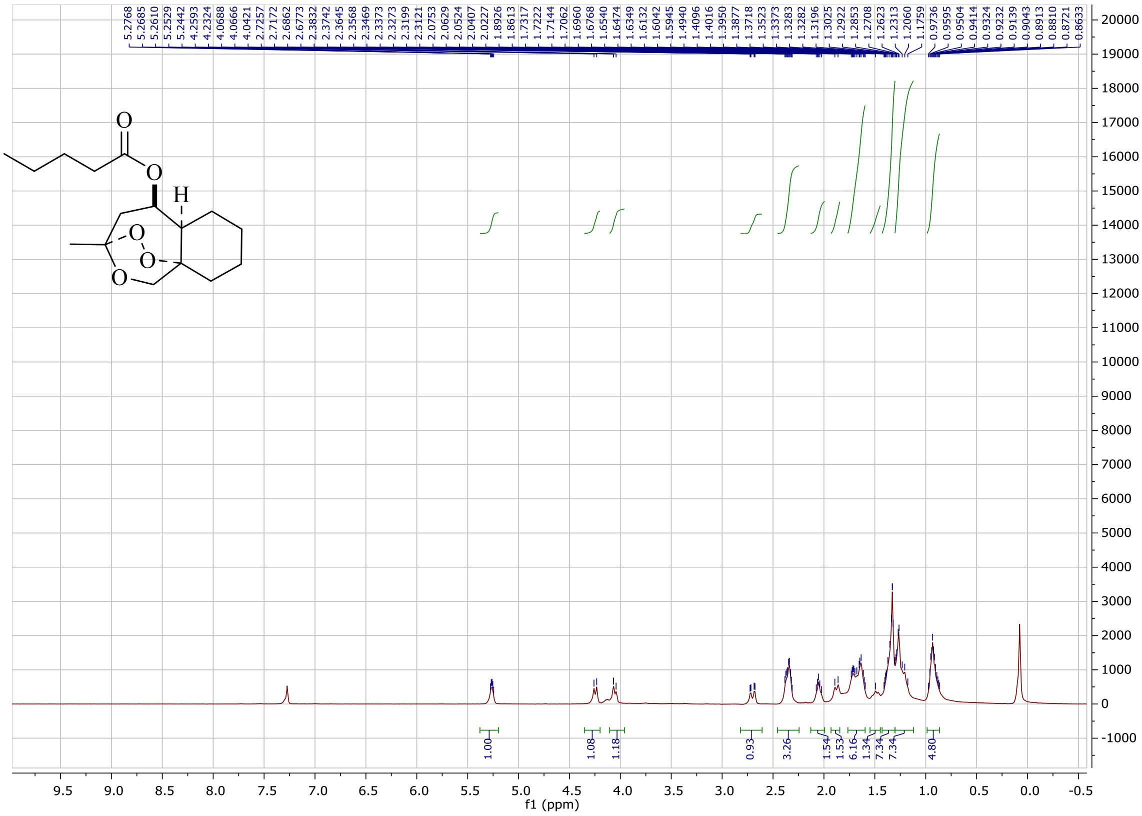

Figure S21. $^{1}$H spectrum of the compound **9** ($CDCl_3$, 400 MHz).

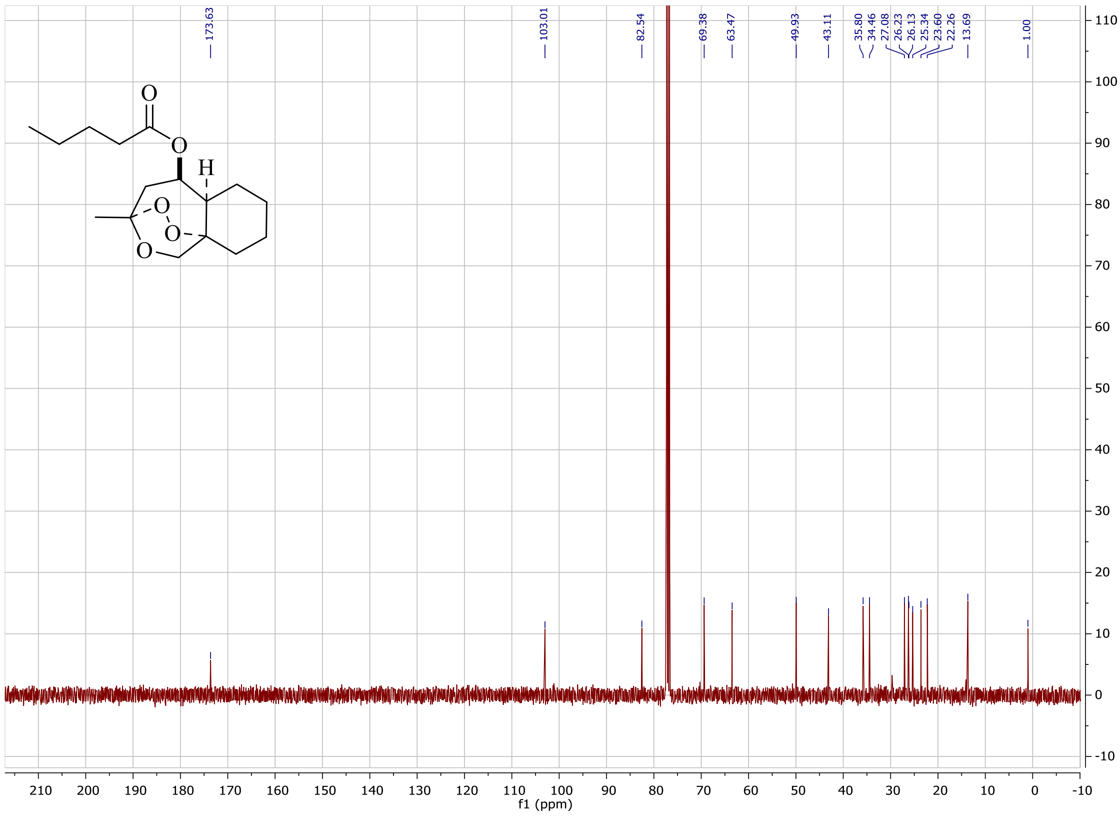


Figure S22. $^{13}$C-NMR spectrum of the compound **9** (CDCl3, 100 MHz).

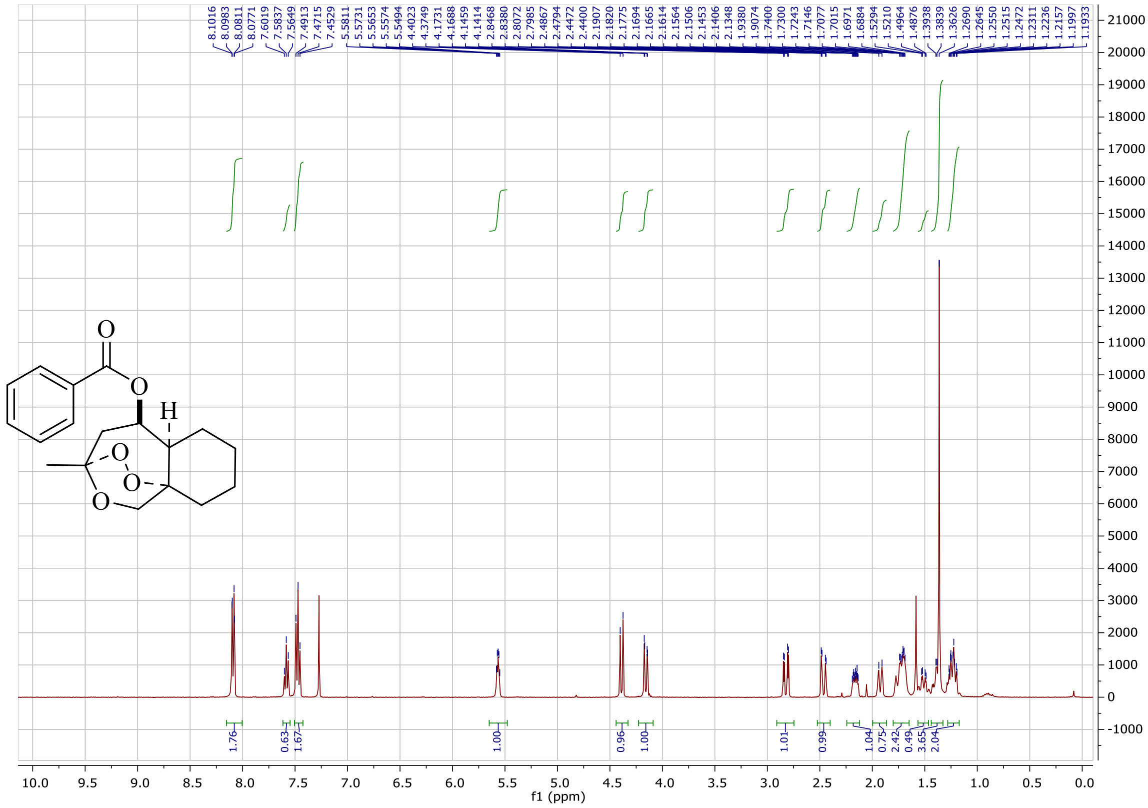

Figure S23. ¹H-spectrum of the compound **10** (CDCl3, 400 MHz).

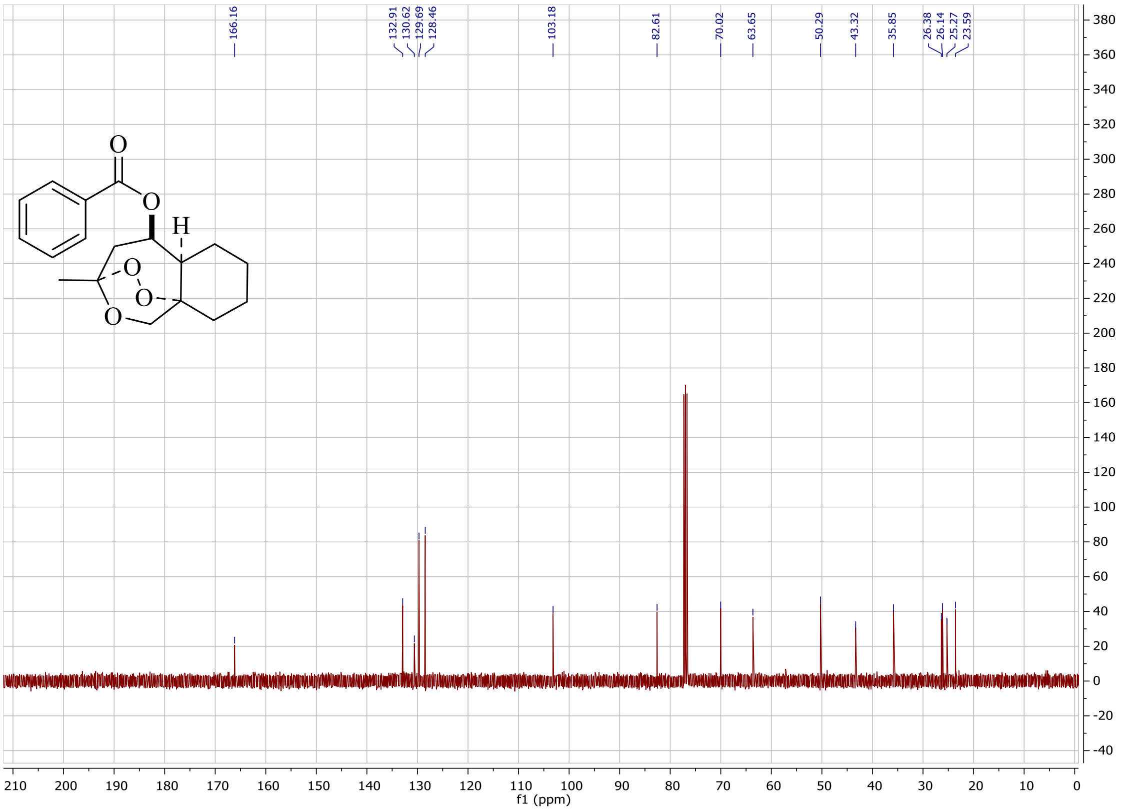


Figure S24. $^{13}$C-NMR spectrum of the compound **10** (CDCl3, 100 MHz).

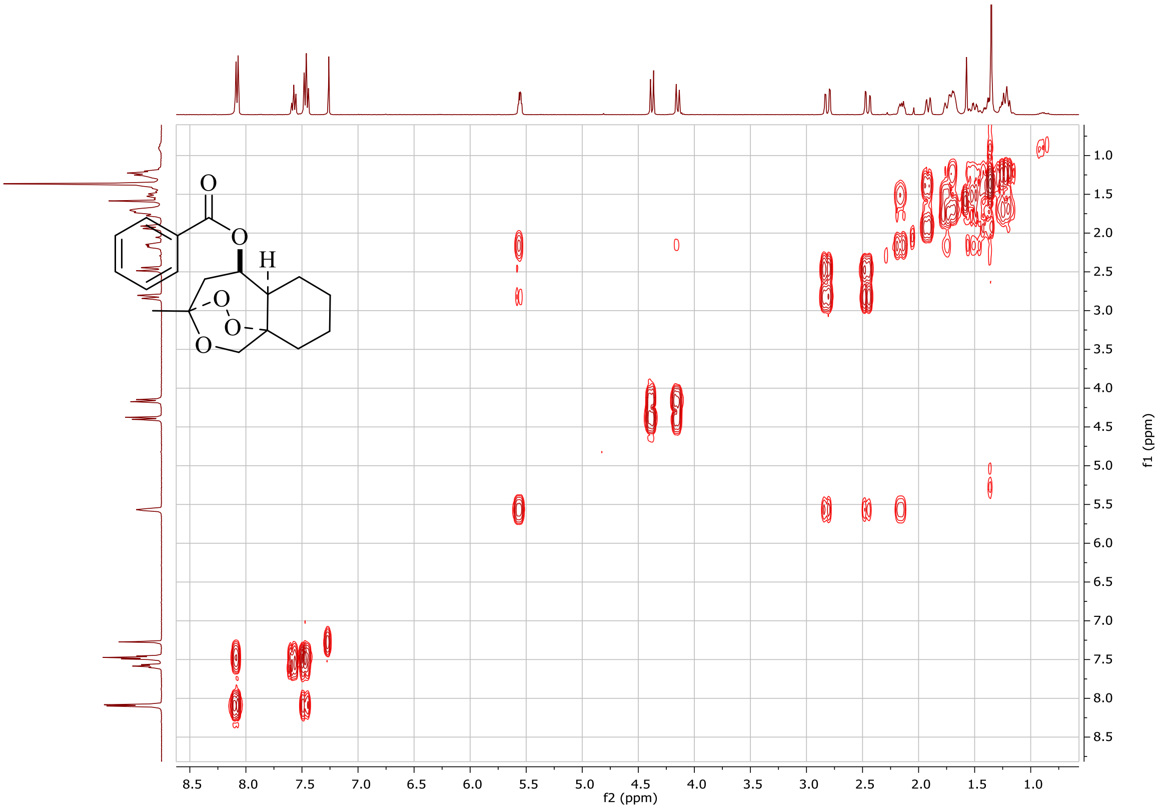


Figure S25. COSY spectrum of the compound **10**.

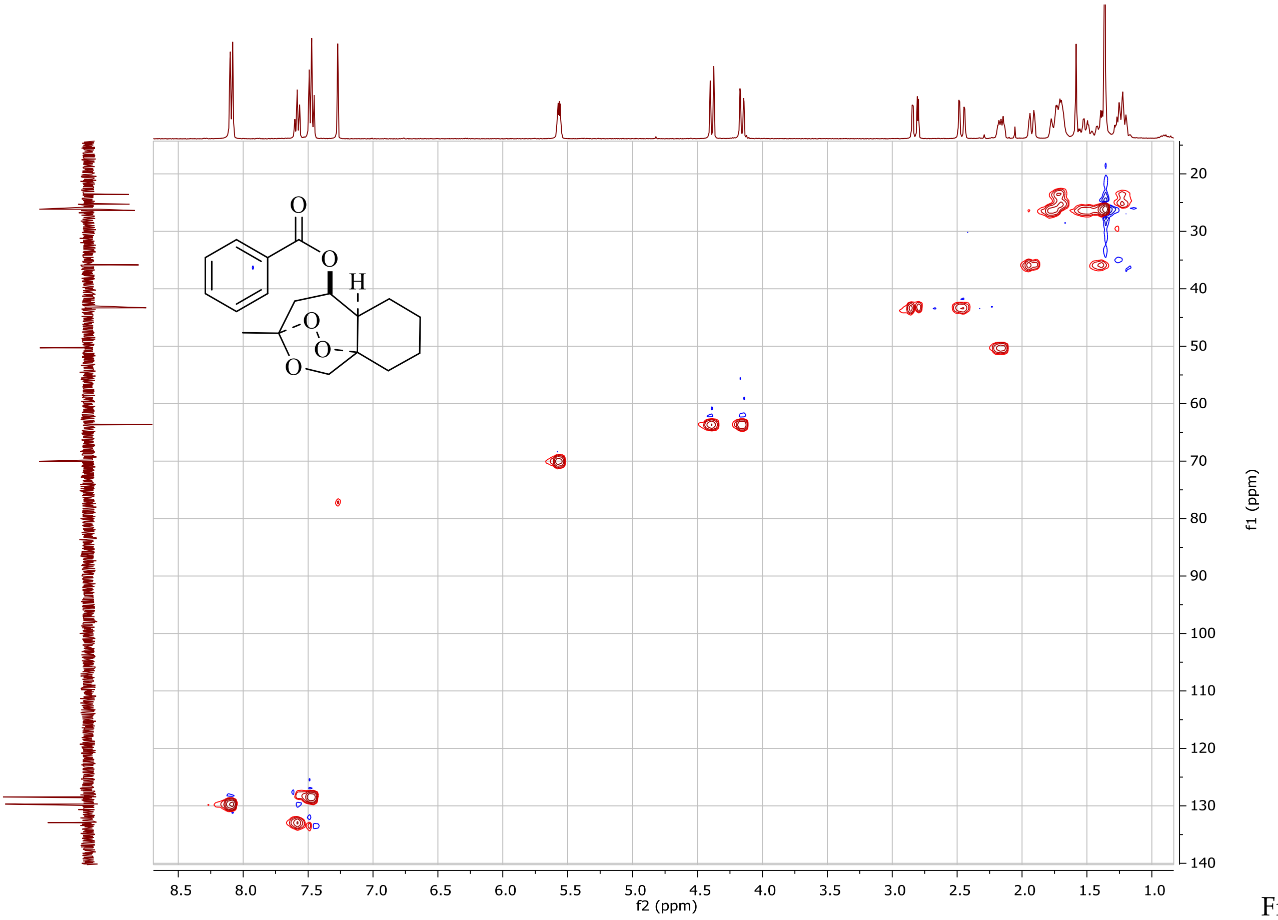


Figure S26. HSQC spectrum of the compound **10**.

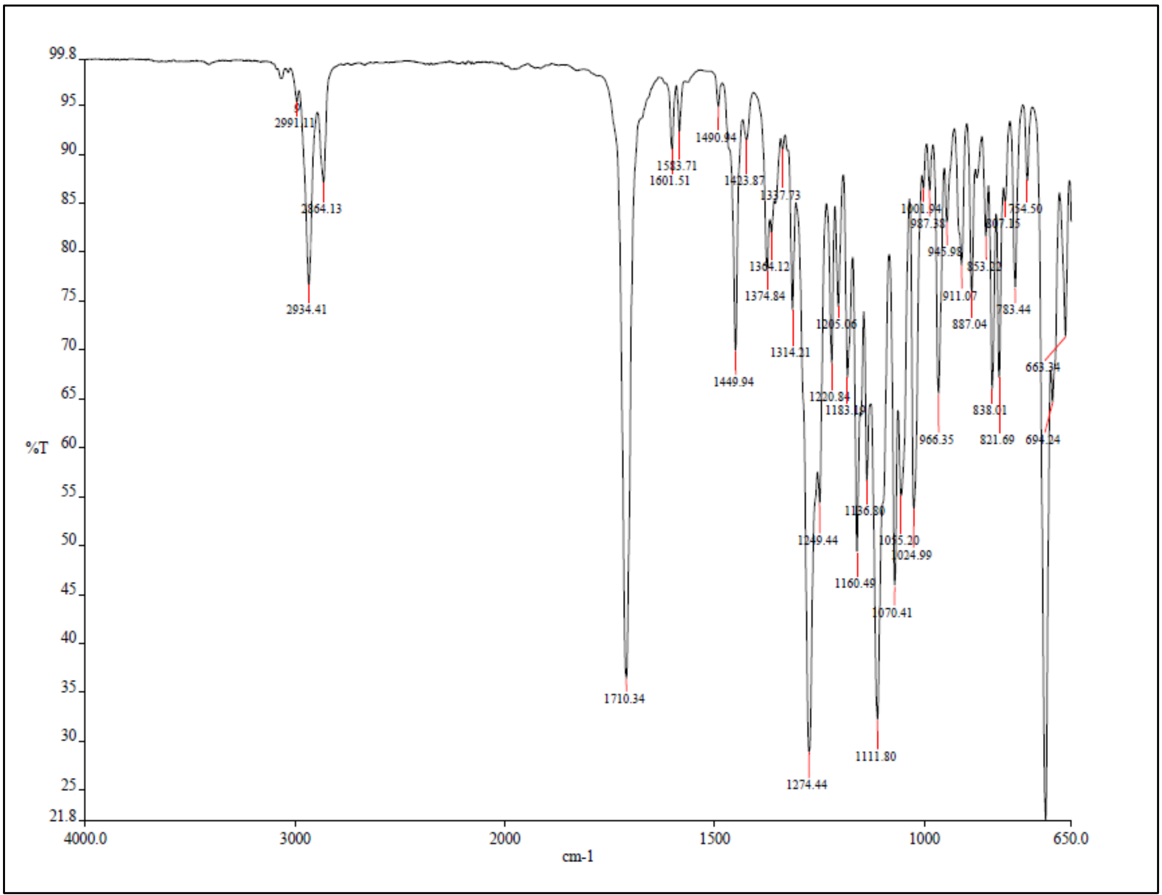


Figure S27. IR-spectrum of the compound **10**.

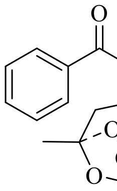

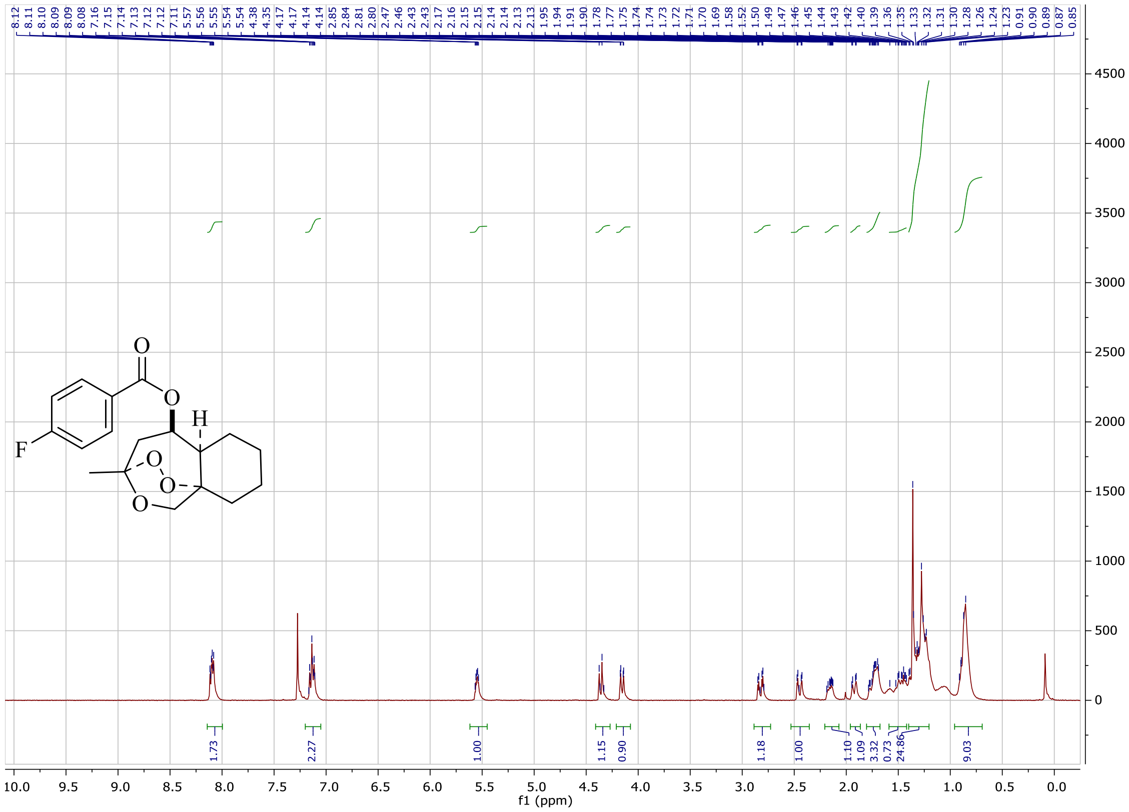

Figure S28. $^{1}$H-spectrum of the compound **11** (CDCl3, 400 MHz).

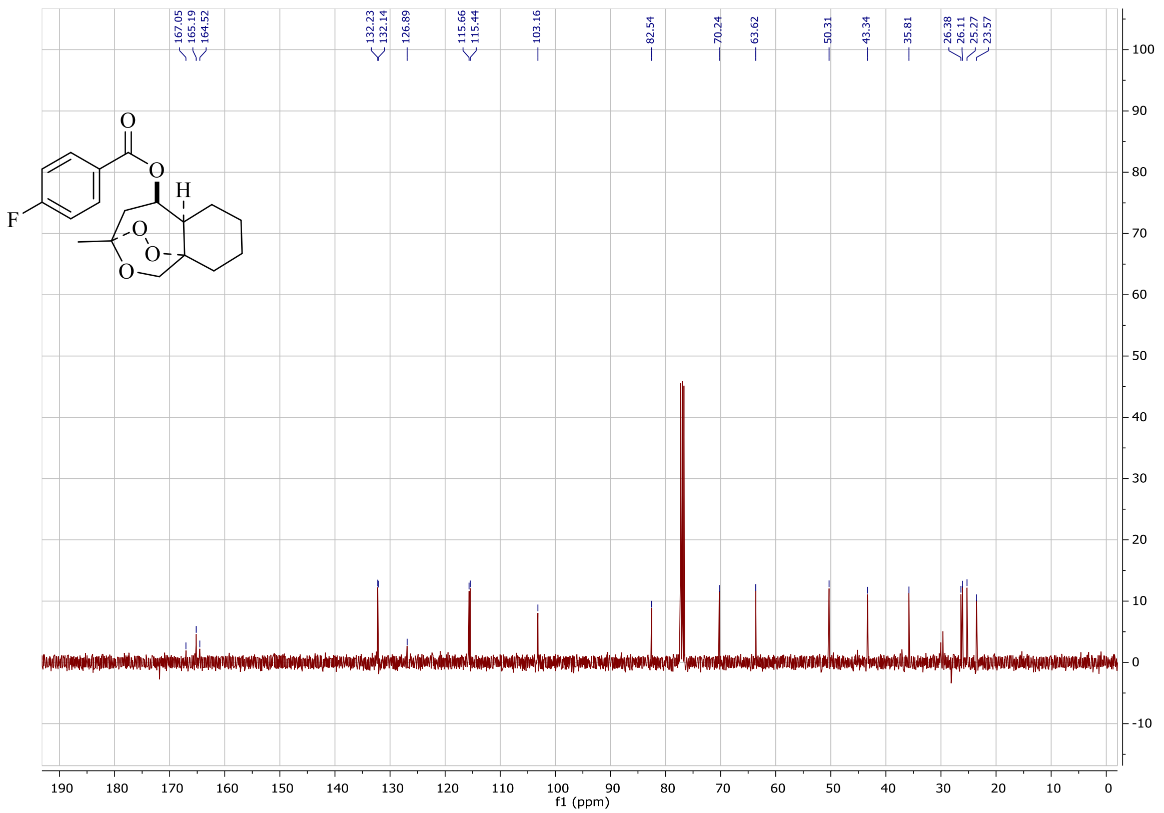


Figure S29. $^{13}$C-NMR spectrum of the compound **11** (CDCl3, 100 MHz).

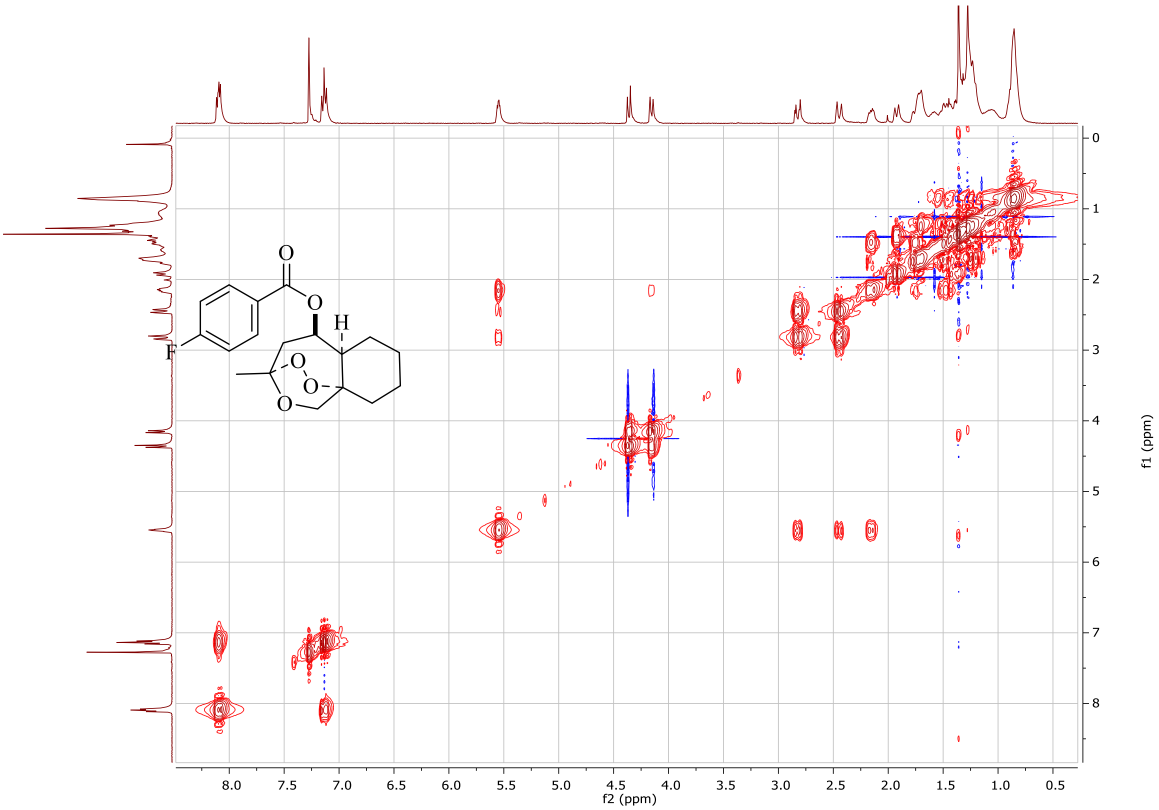


Figure S30. COSY spectrum of the compound **11**.

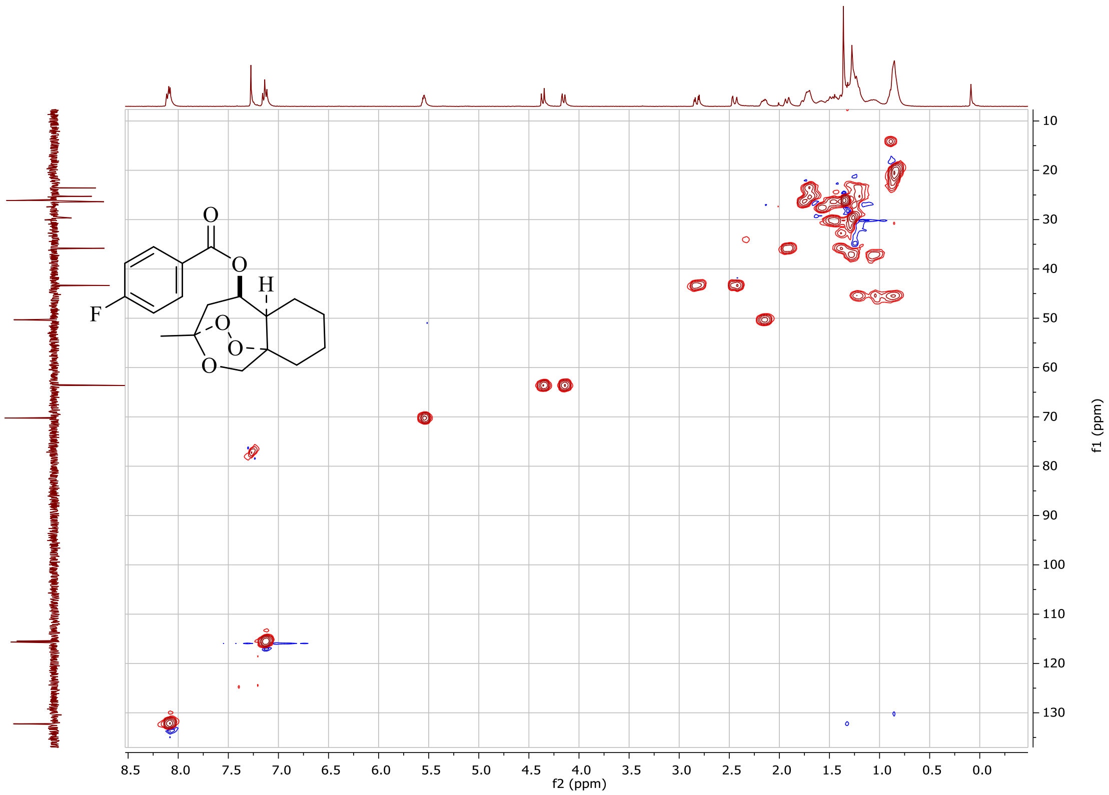


Figure S31. HSQC spectrum of the compound **11**.

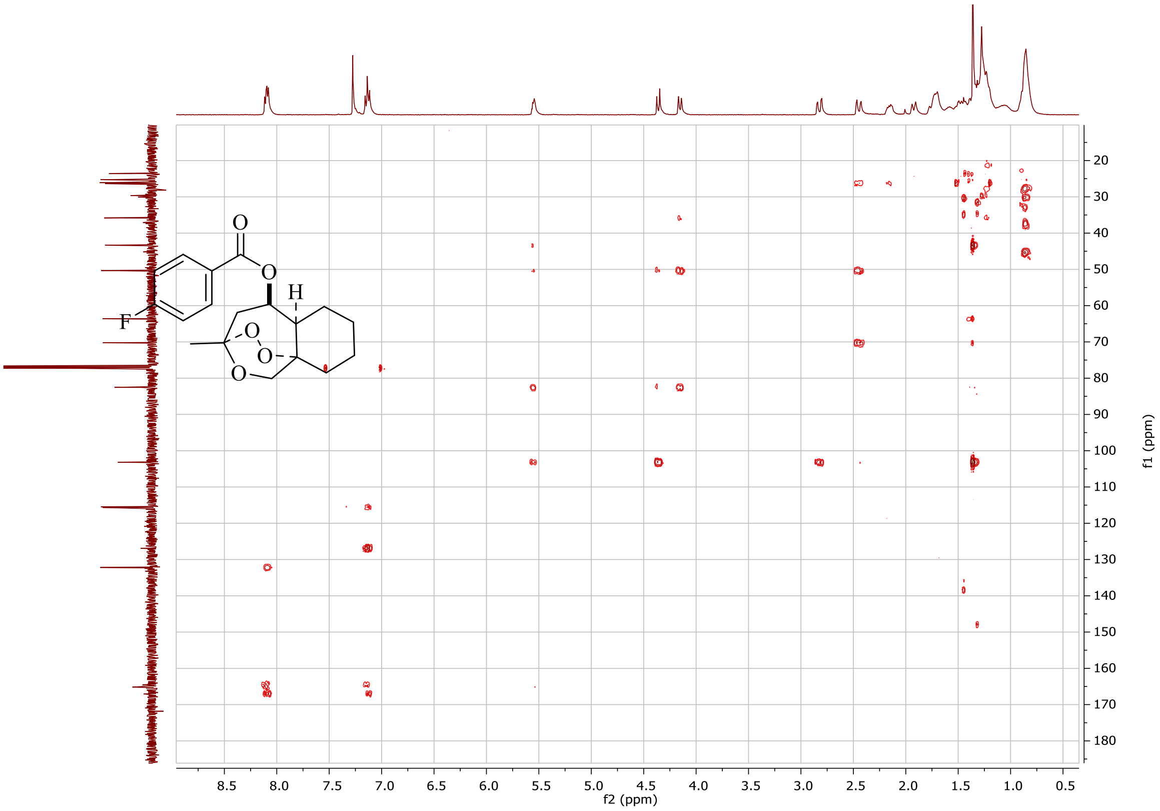


Figure S32. HMBC spectrum of the compound **11**.

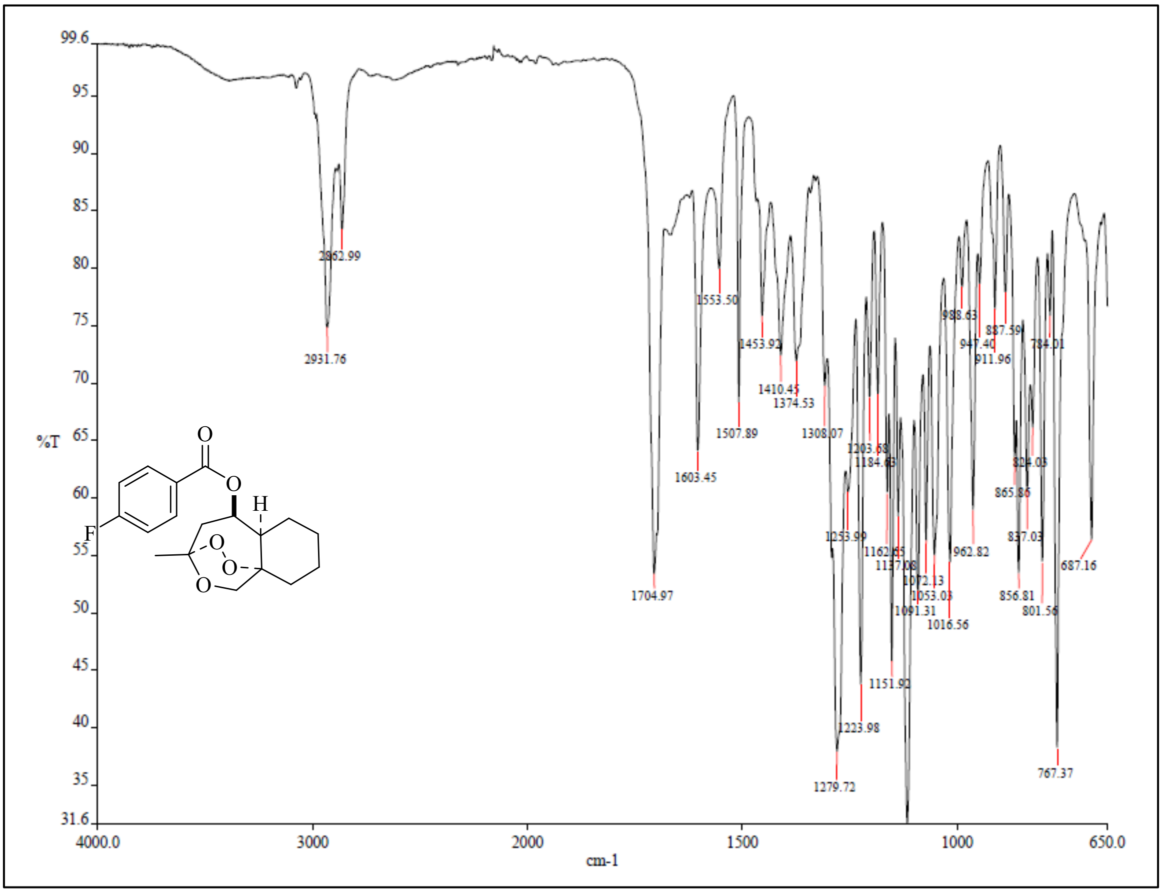


Figure S33. IR-spectrum of the compound **11**.

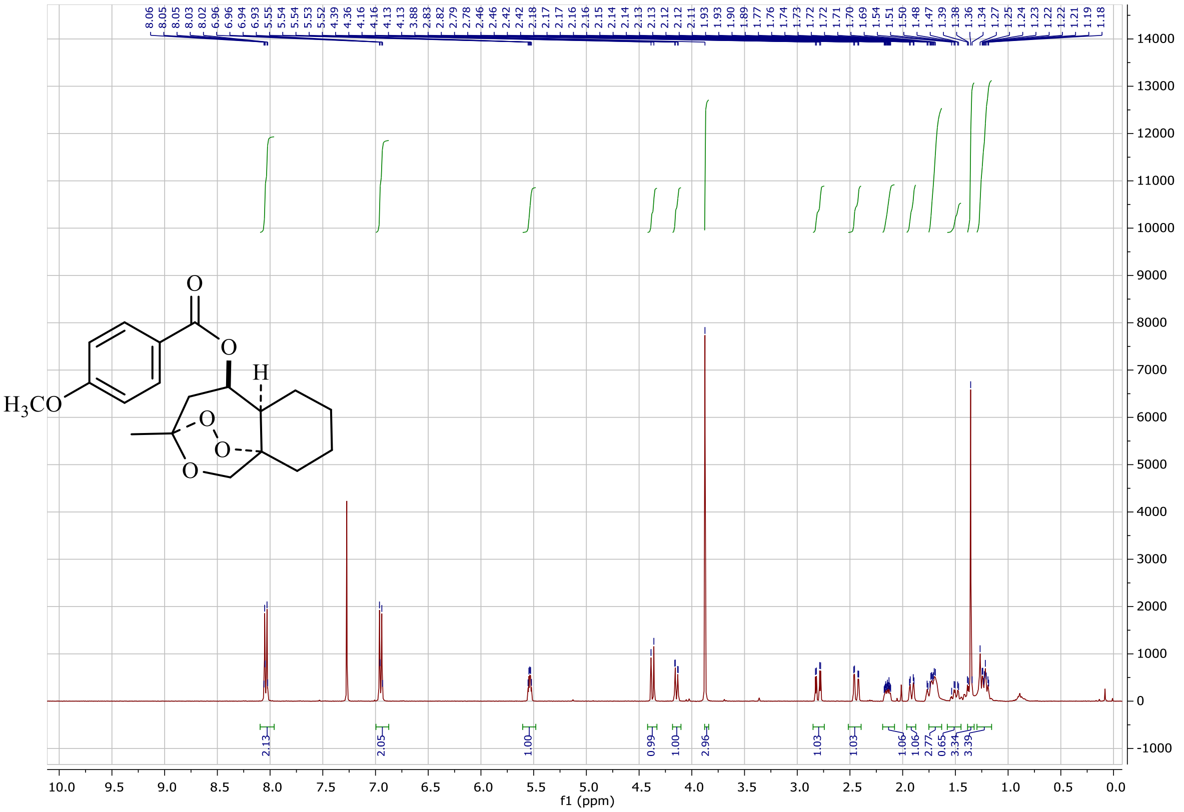

Figure S34. $^{1}$H-spectrum of the compound **12** (CDCl3, 400 MHz).

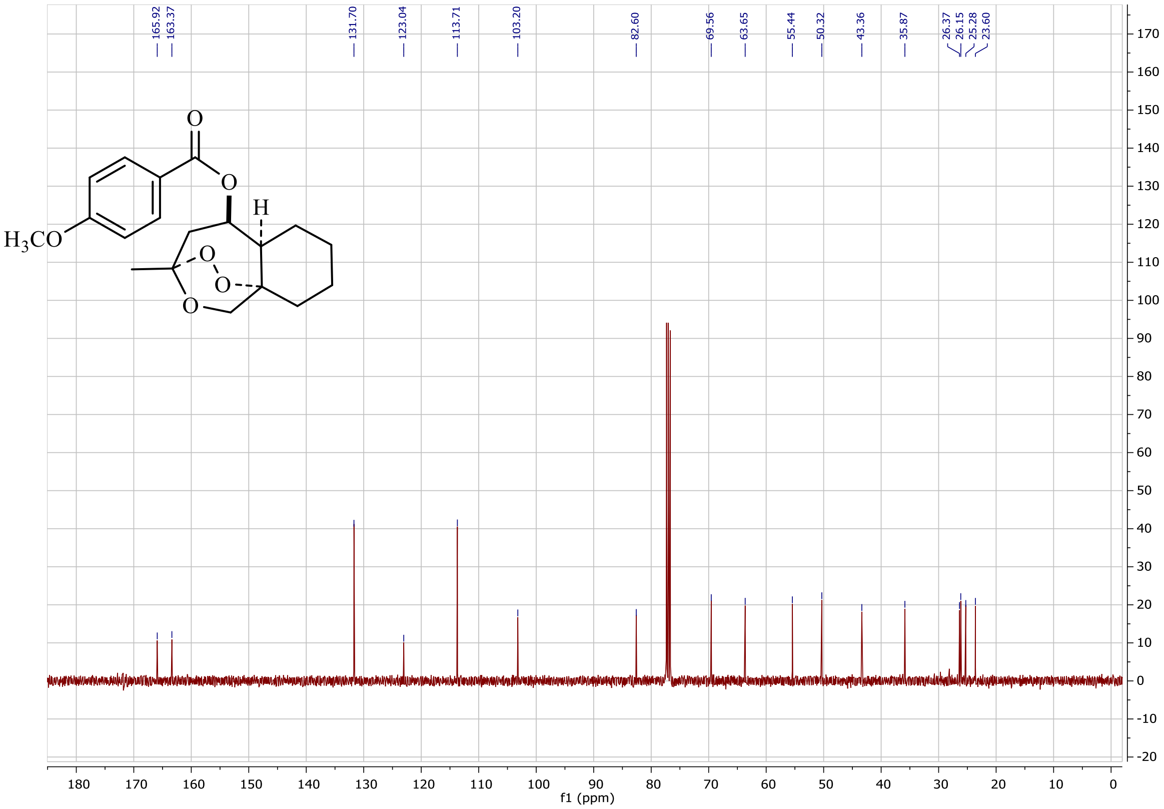


Figure S35. $^{13}$C-NMR spectrum of the compound **12** (CDCl3, 100 MHz).

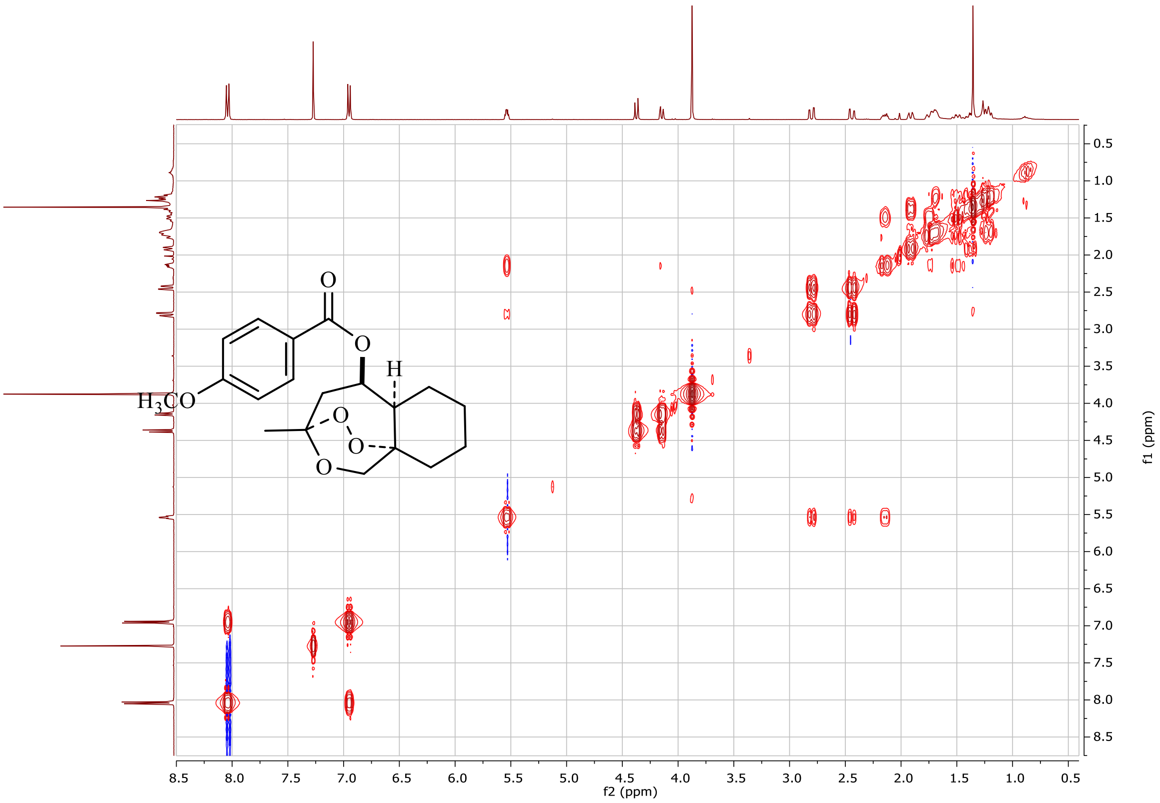


Figure S36. COSY spectrum of the compound **12**.

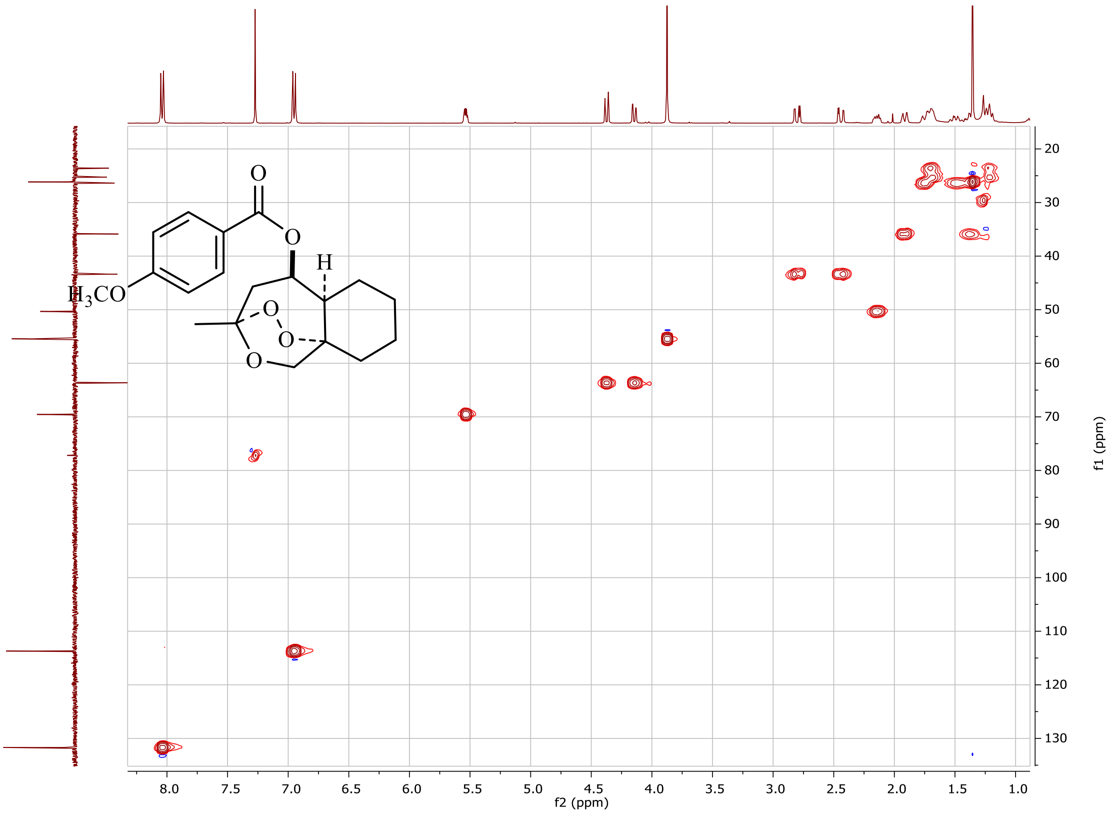


Figure S37. HSQC spectrum of the compound **12**.

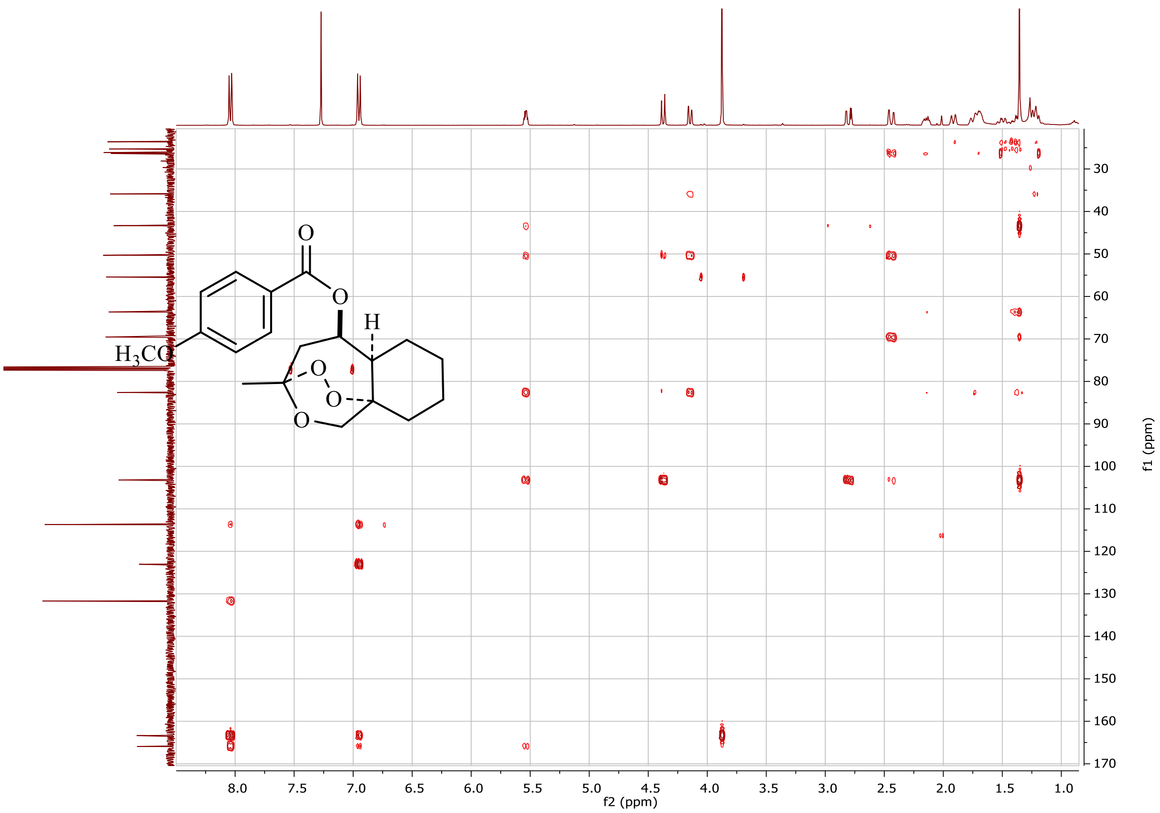


Figure S38. HMBC spectrum of the compound **12**.

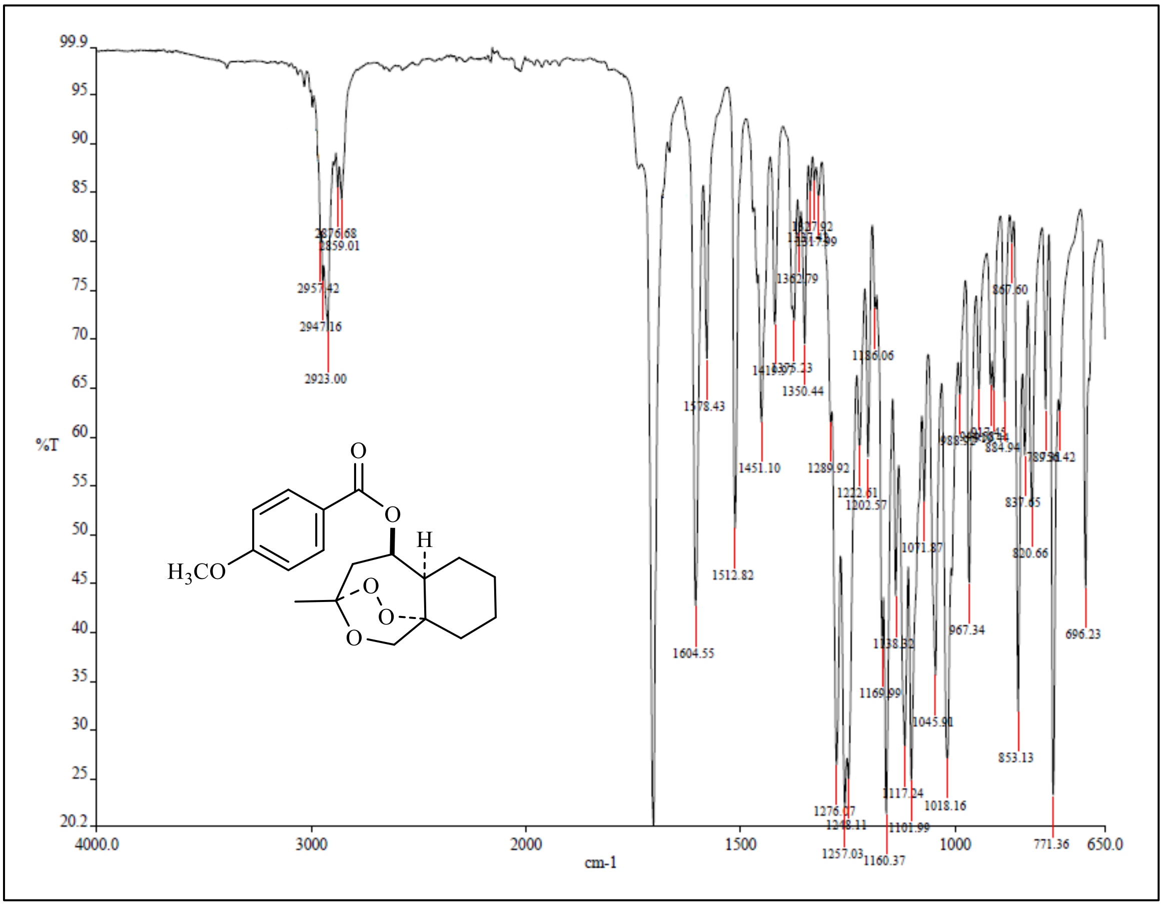


Figure S39. IR-spectrum of the compound **12**.

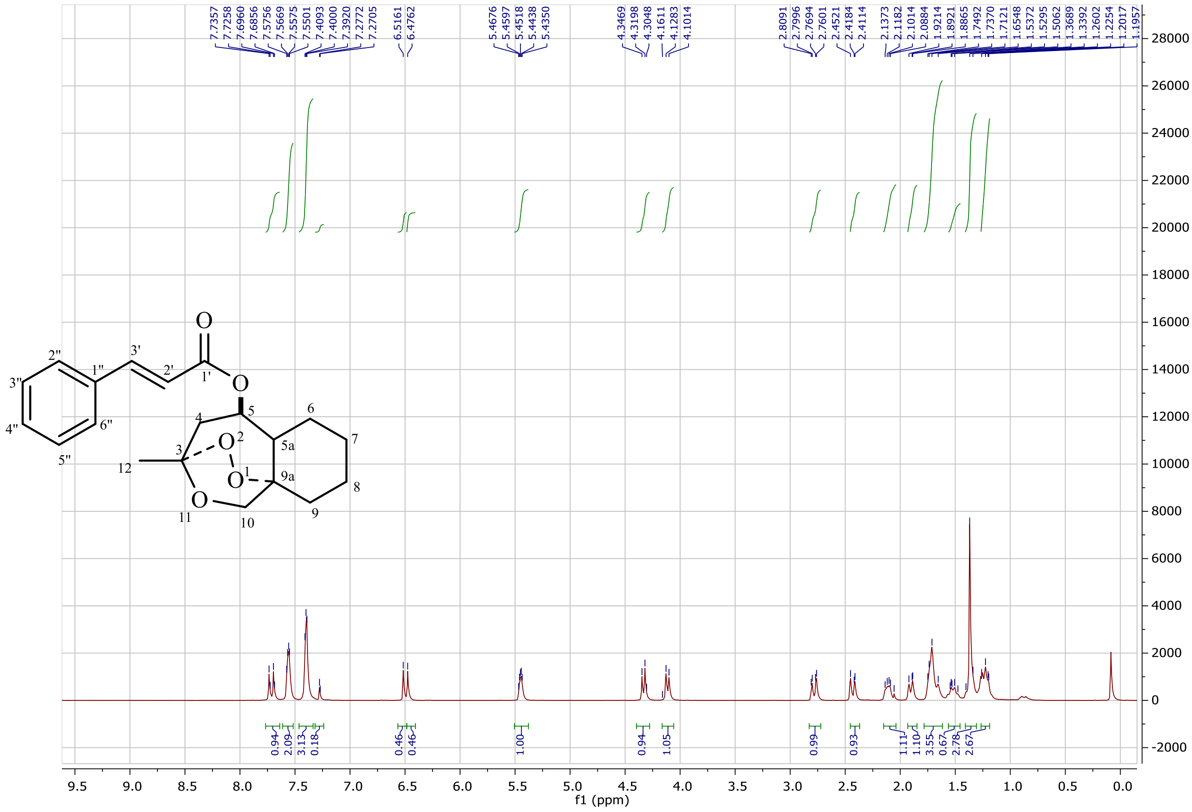


Figure S40. $^{1}$H-spectrum of the compound **13** (CDCl3, 400 MHz).

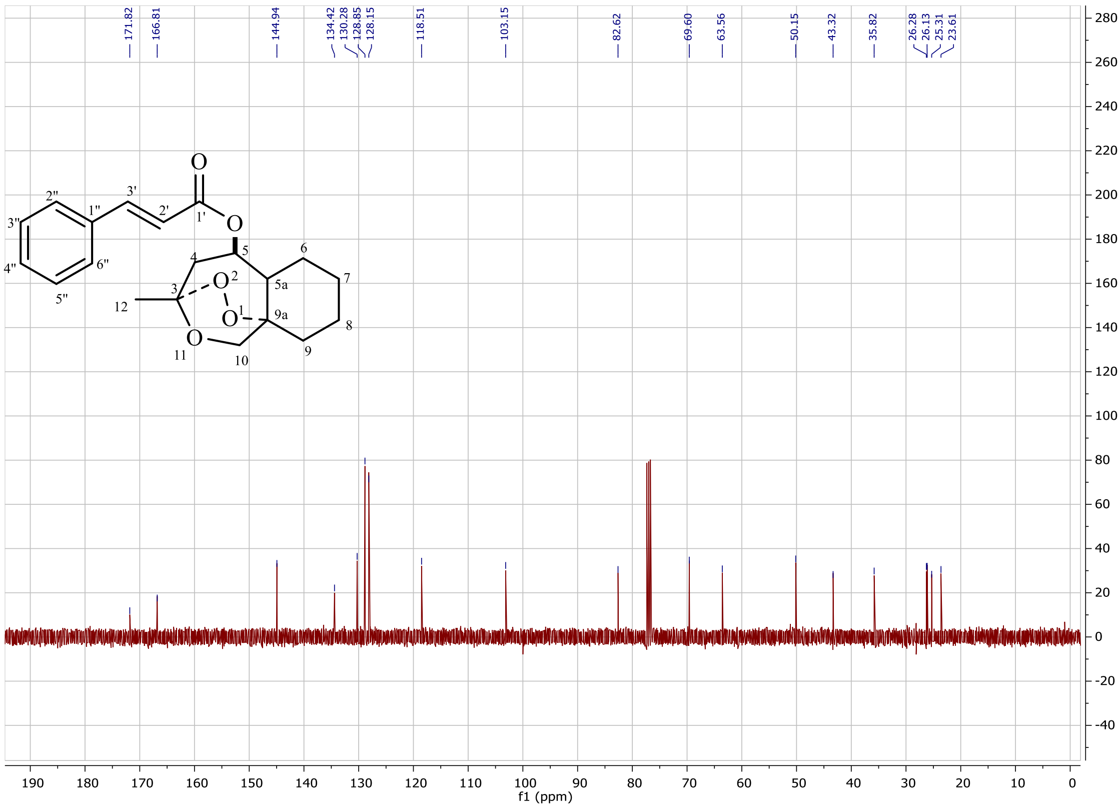


Figure S41. $^{13}$C-NMR spectrum of the compound **13** (CDCl3, 100 MHz).

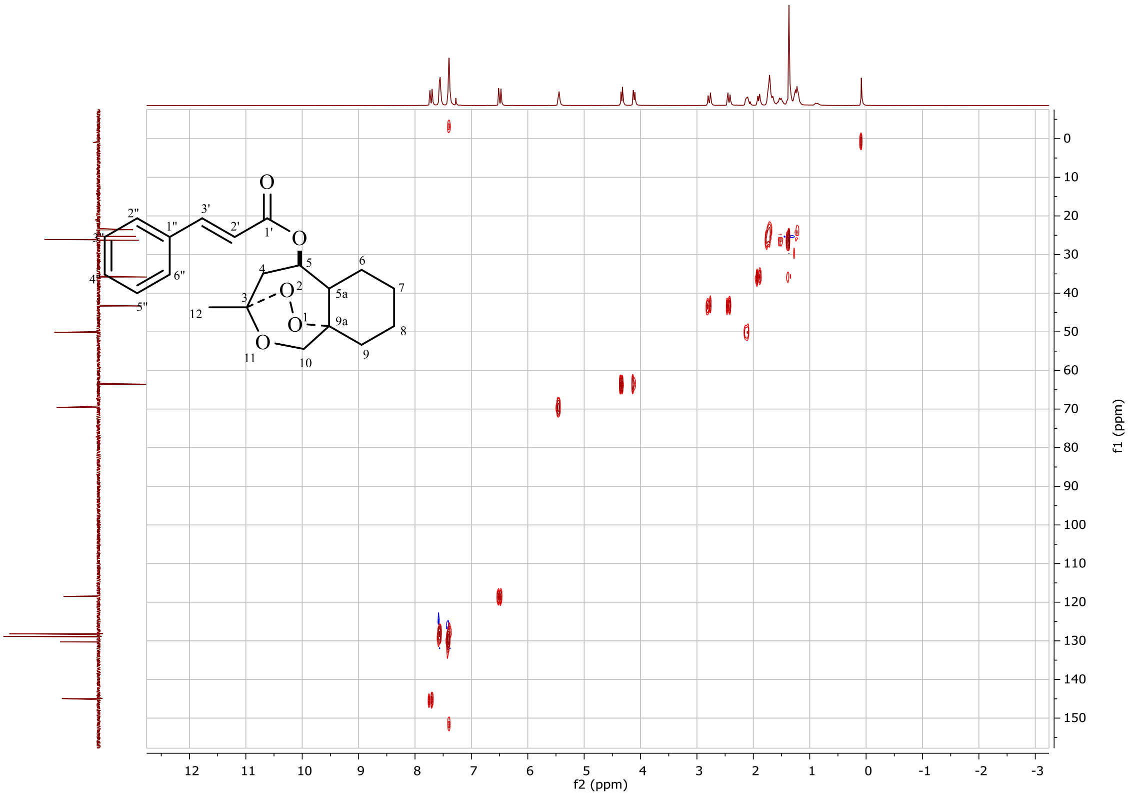


Figure S42. HSQC spectrum of the compound **13**.

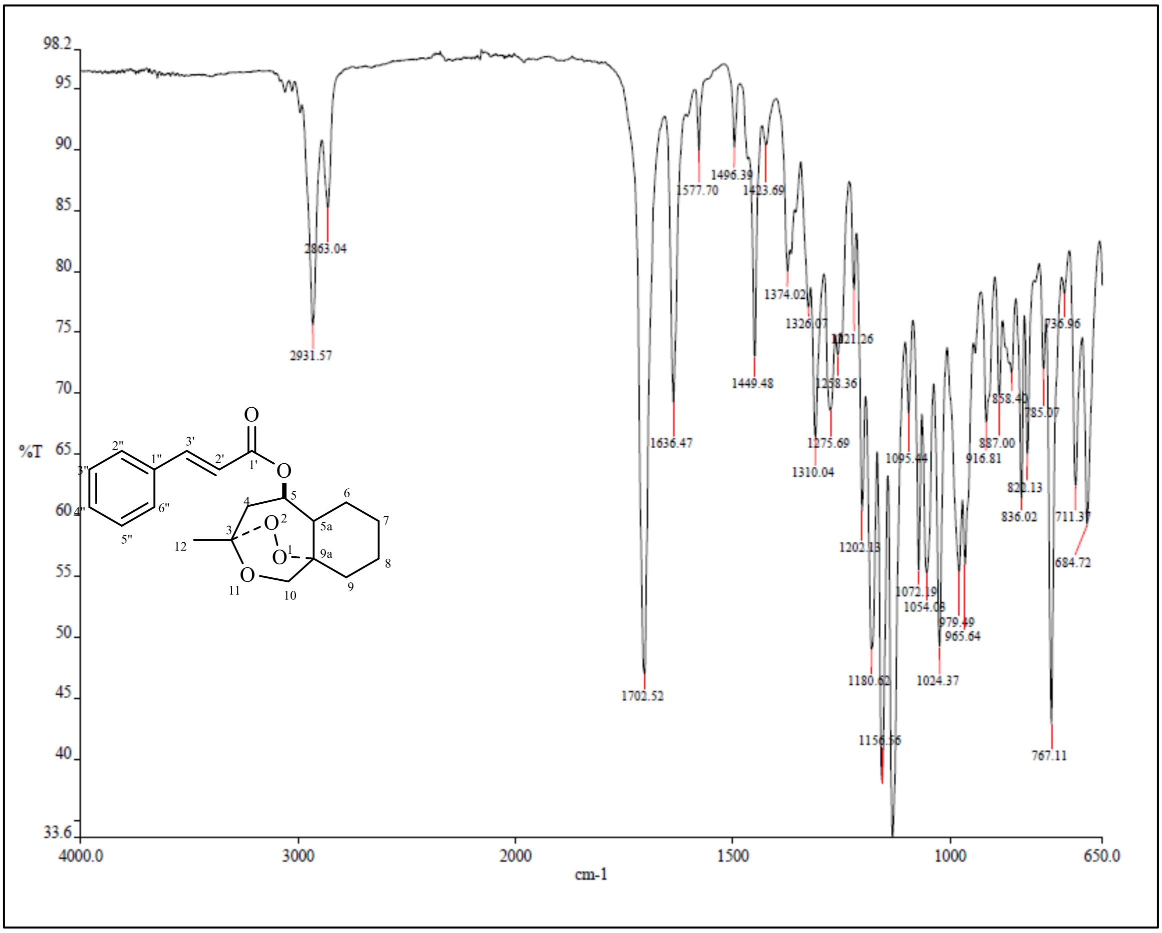


Figure S43. IR-spectrum of the compound **13**.

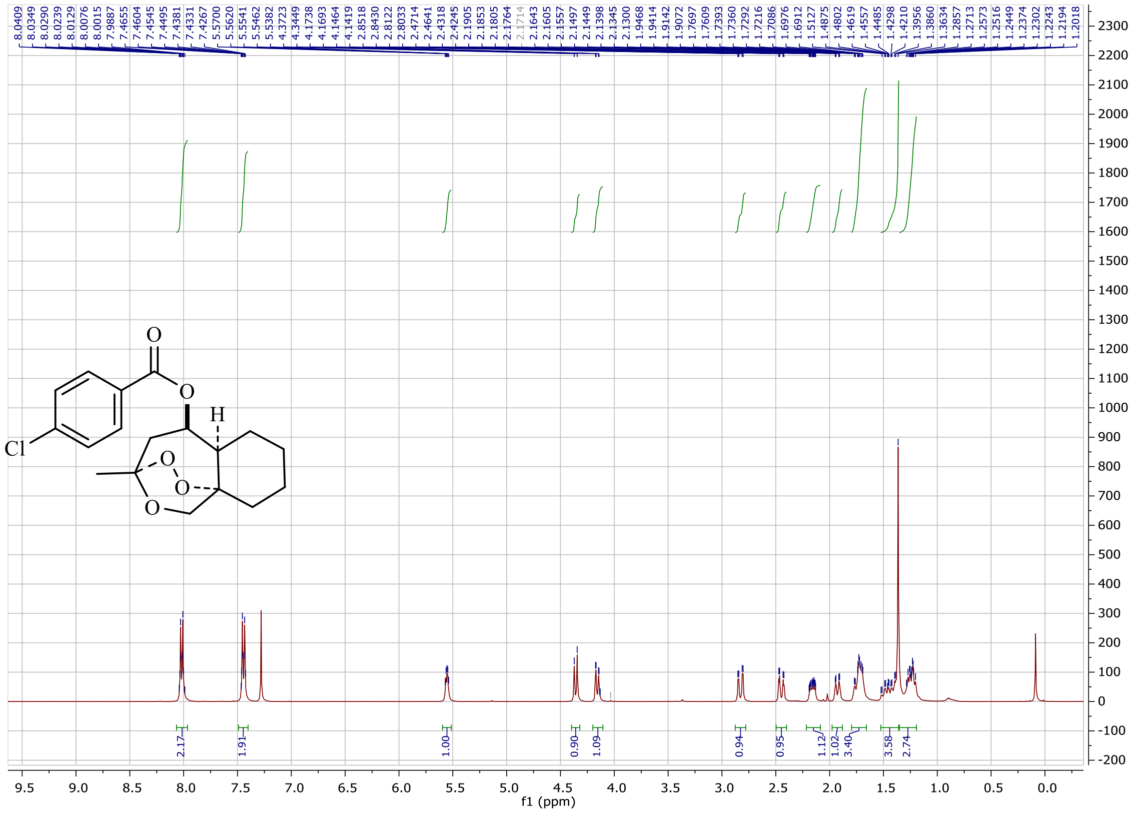

Figure S44. $^{1}$H-spectrum of the compound **14** (CDCl3, 400 MHz).

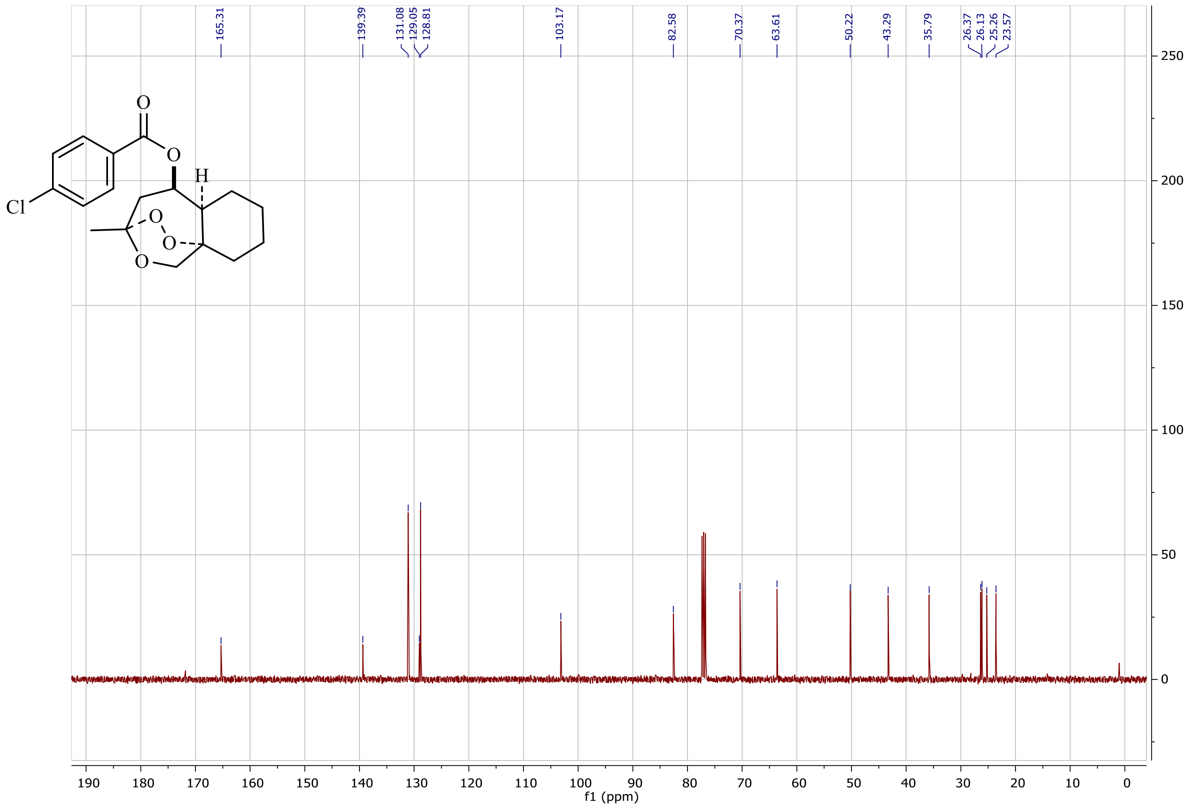


Figure S45. $^{13}$C-NMR spectrum of the compound **14** (CDCl3, 100 MHz).

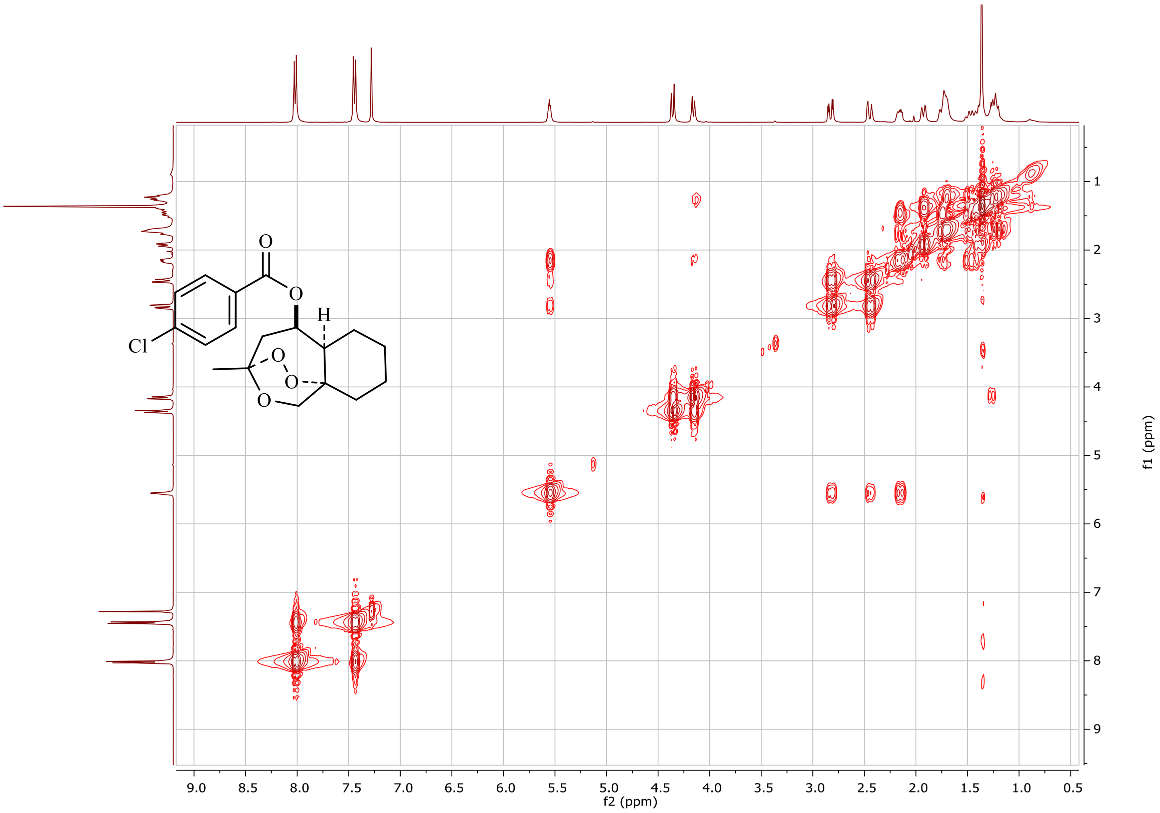


Figure S46. COSY spectrum of the compound **14**.

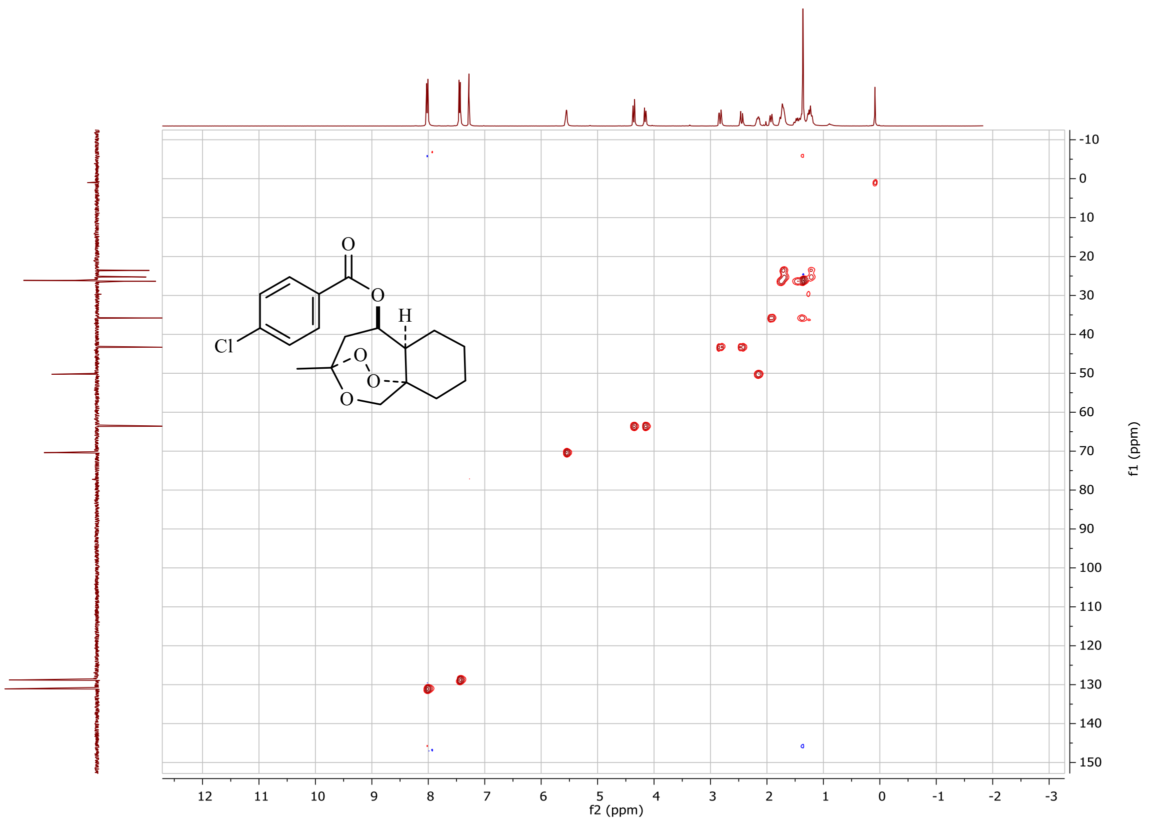


Figure S47. HSQC spectrum of the compound **14**.

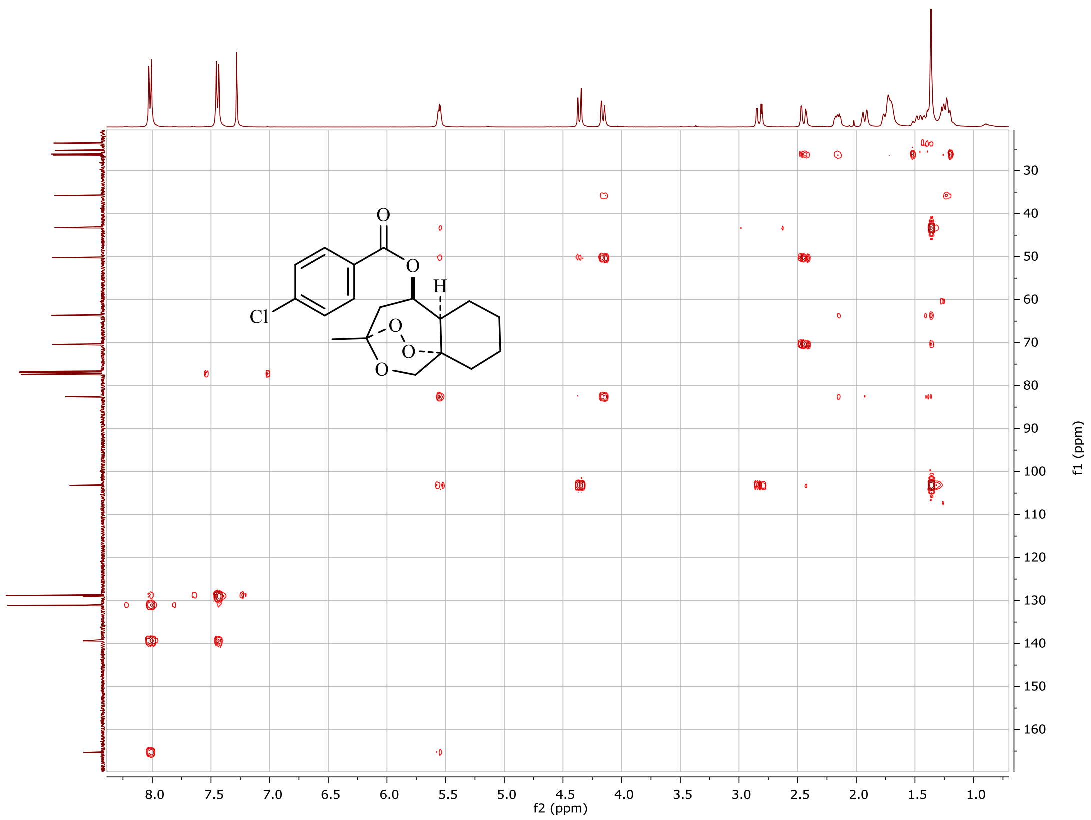


Figure S48.

HMBC spectrum of the compound **14**.

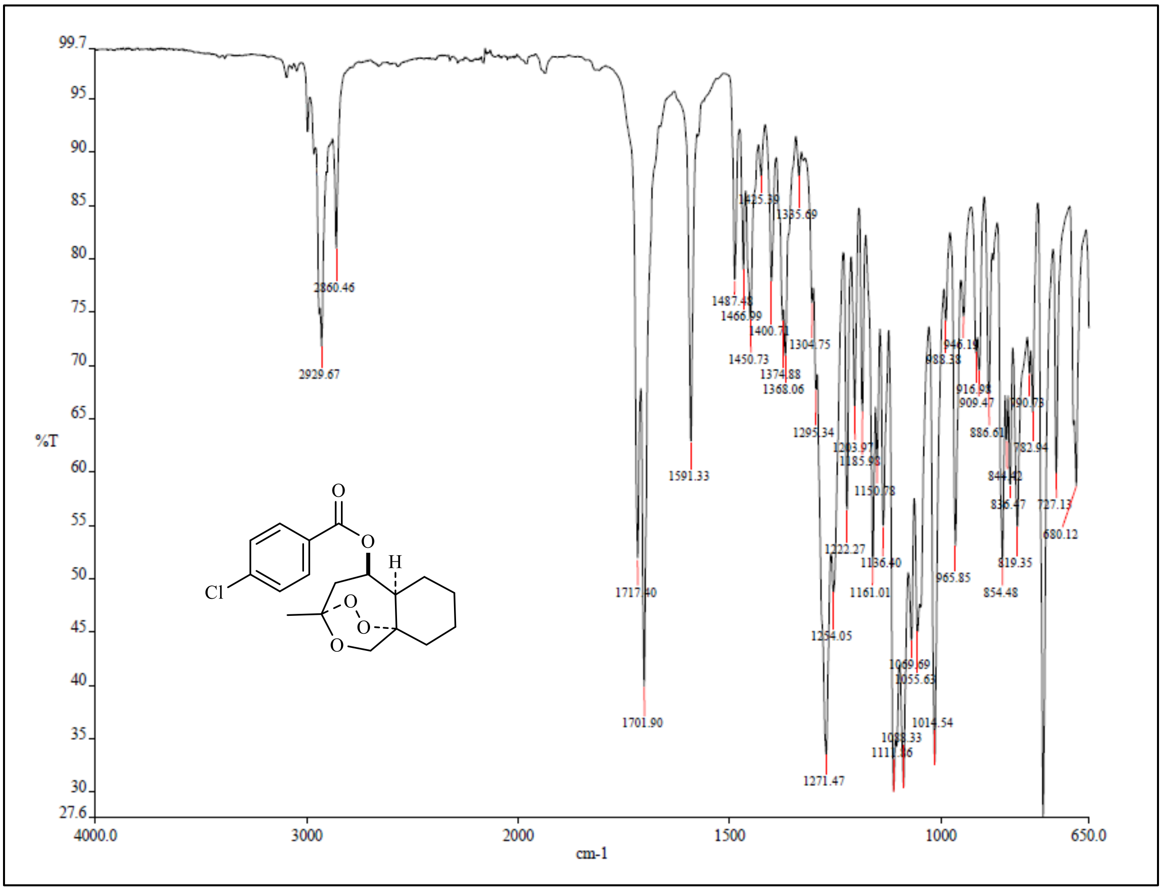

Figure S49. IR-spectrum of the compound **14**.

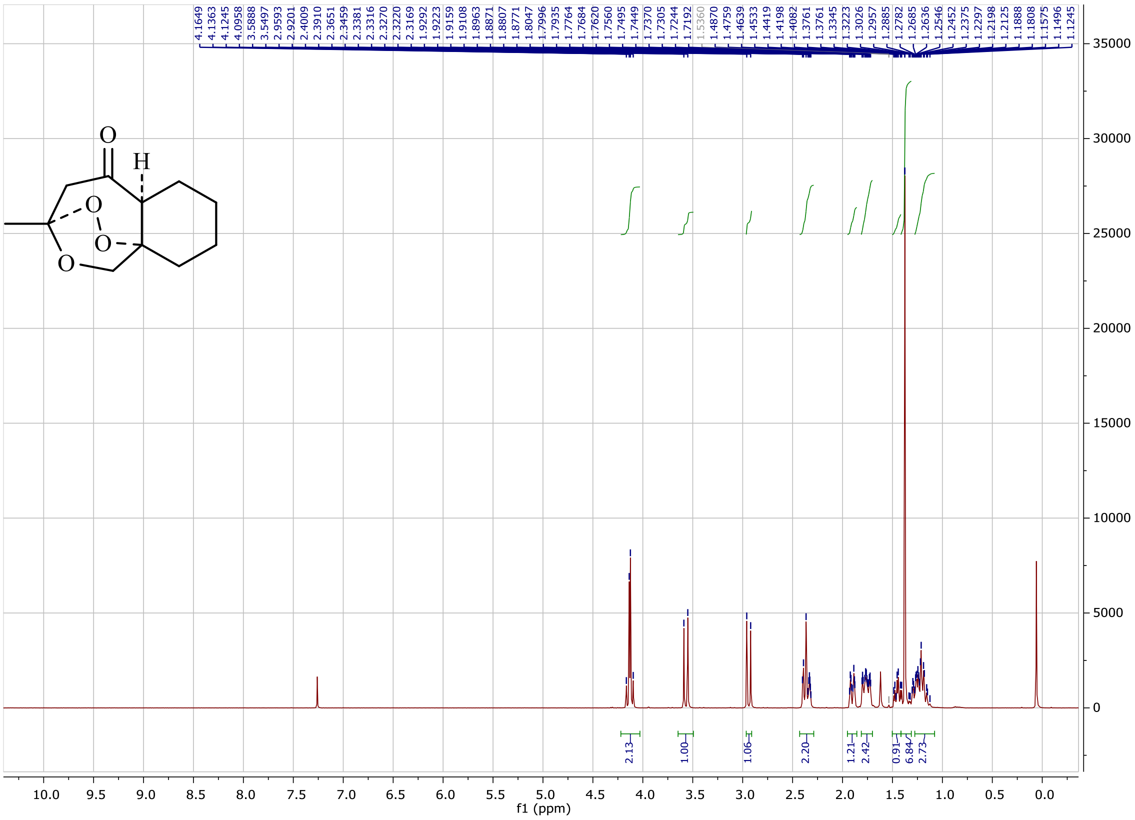

Figure S50. $^{1}$H-spectrum of the compound **15** ($CDCl_3$, 400 MHz).

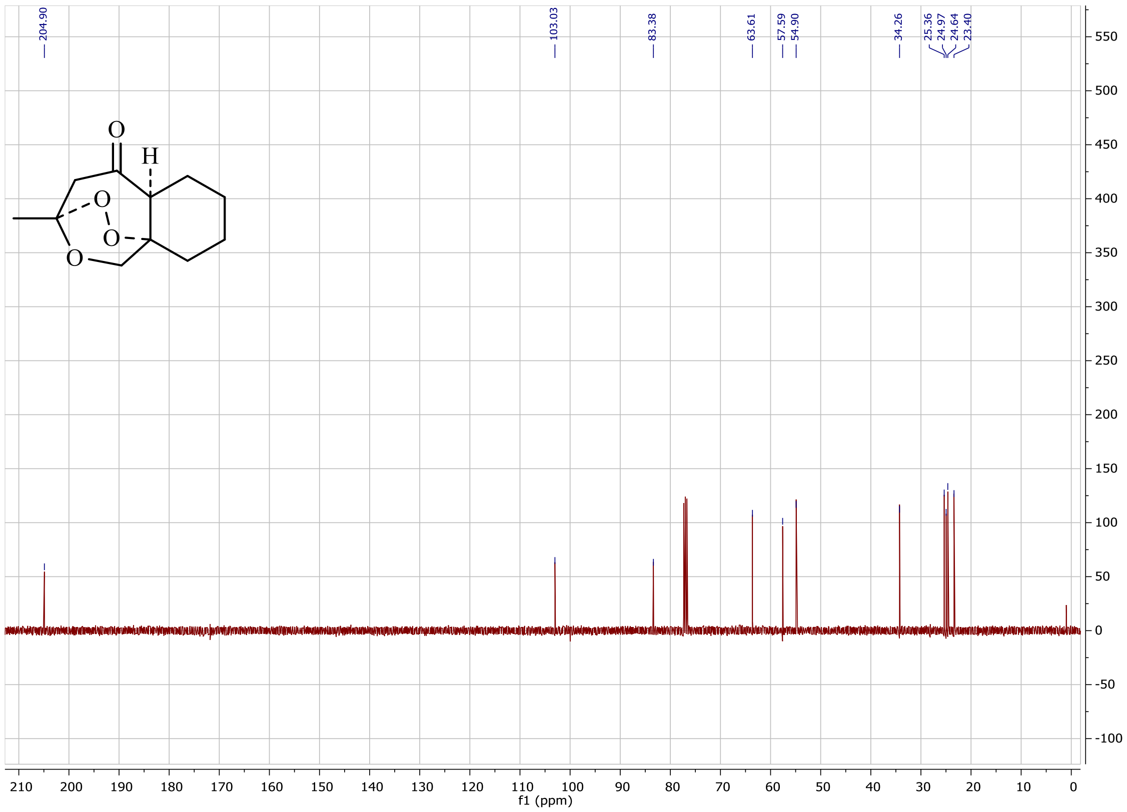


Figure S51. $^{13}$C-NMR spectrum of the compound **15** (CDCl3, 100 MHz).

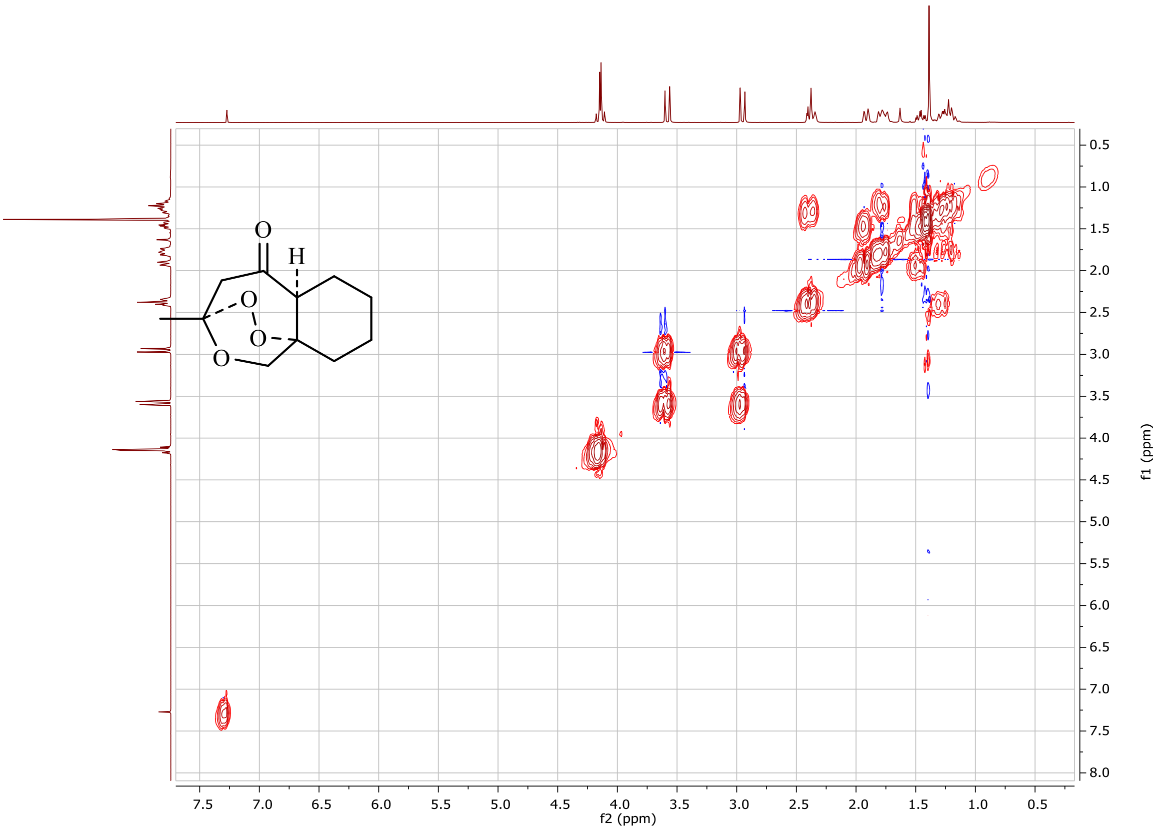


Figure S52. COSY spectrum of the compound **15**.

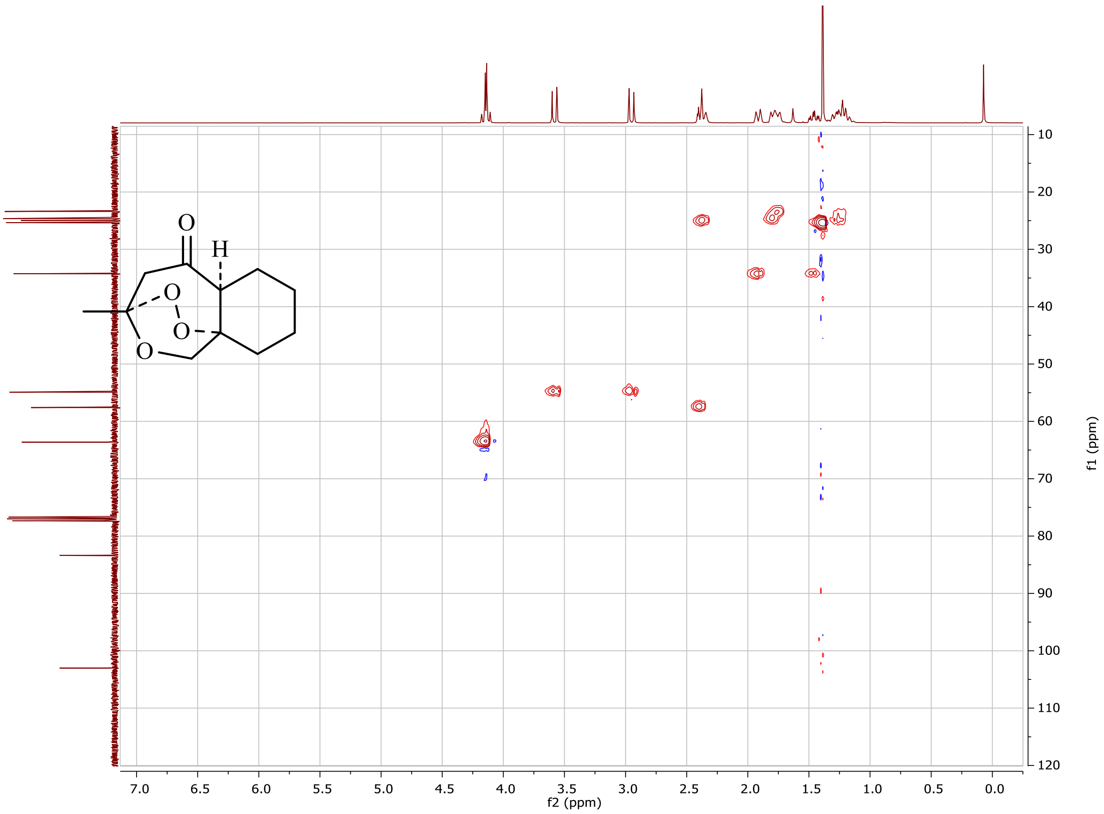


Figure S53. HSQC spectrum of the compound **15**.

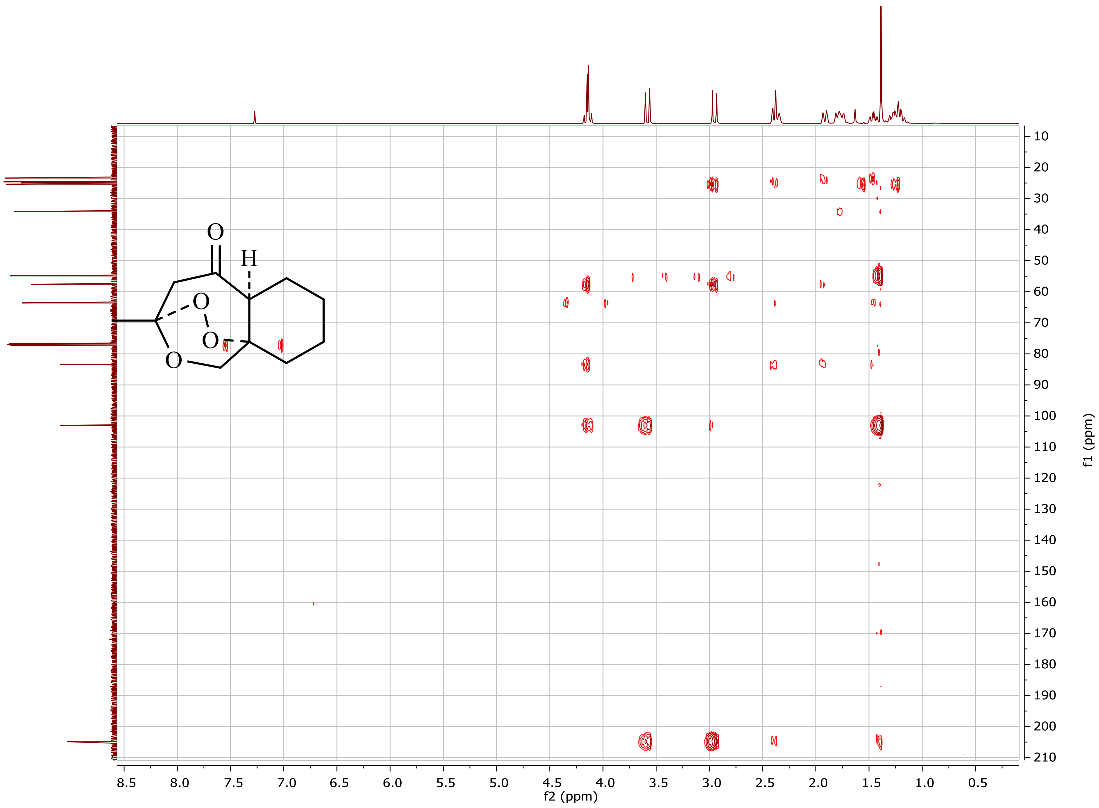


Figure S54. HMBC spectrum of the compound **15**.

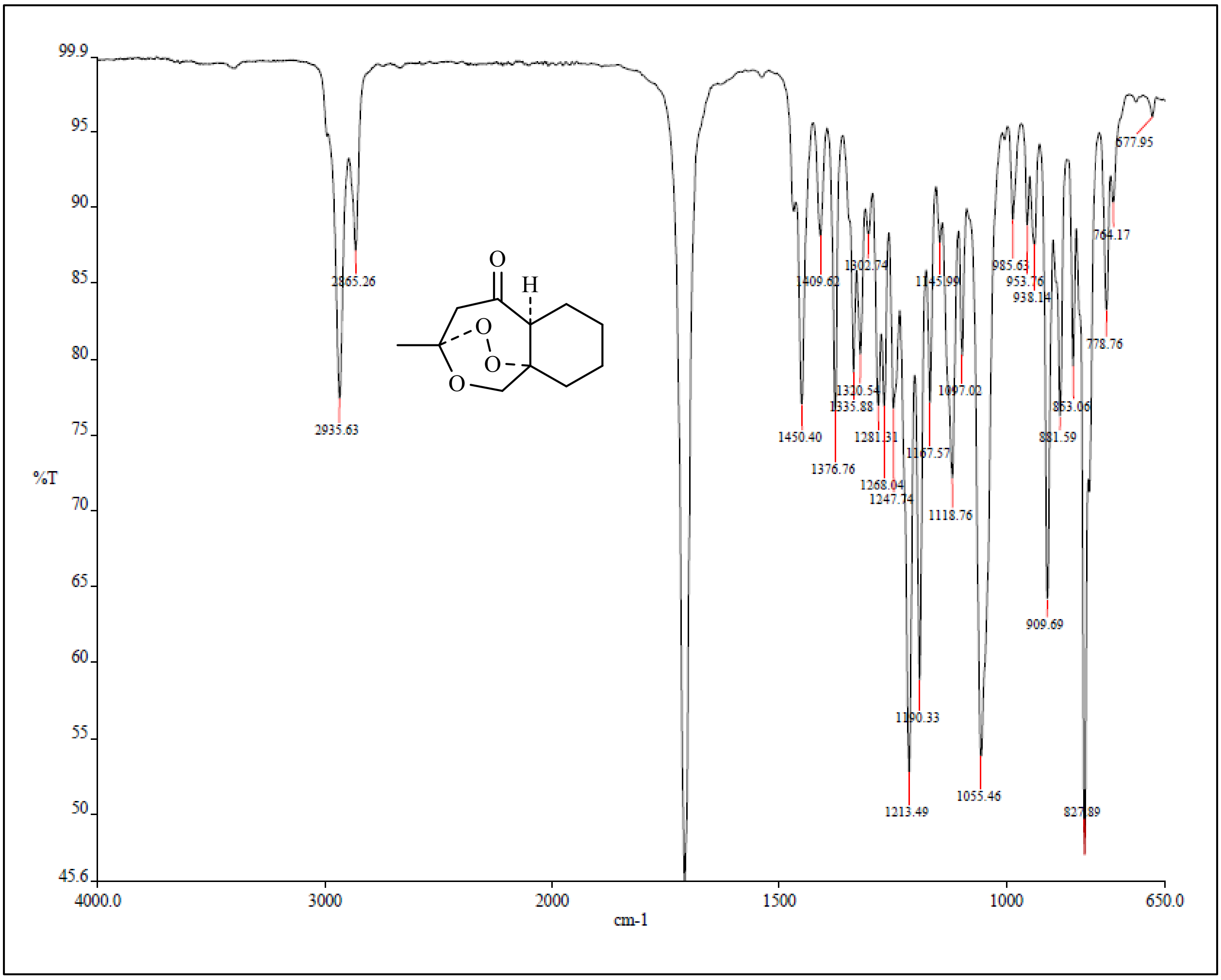


Figure S55. IR-spectrum of the compound **15**.

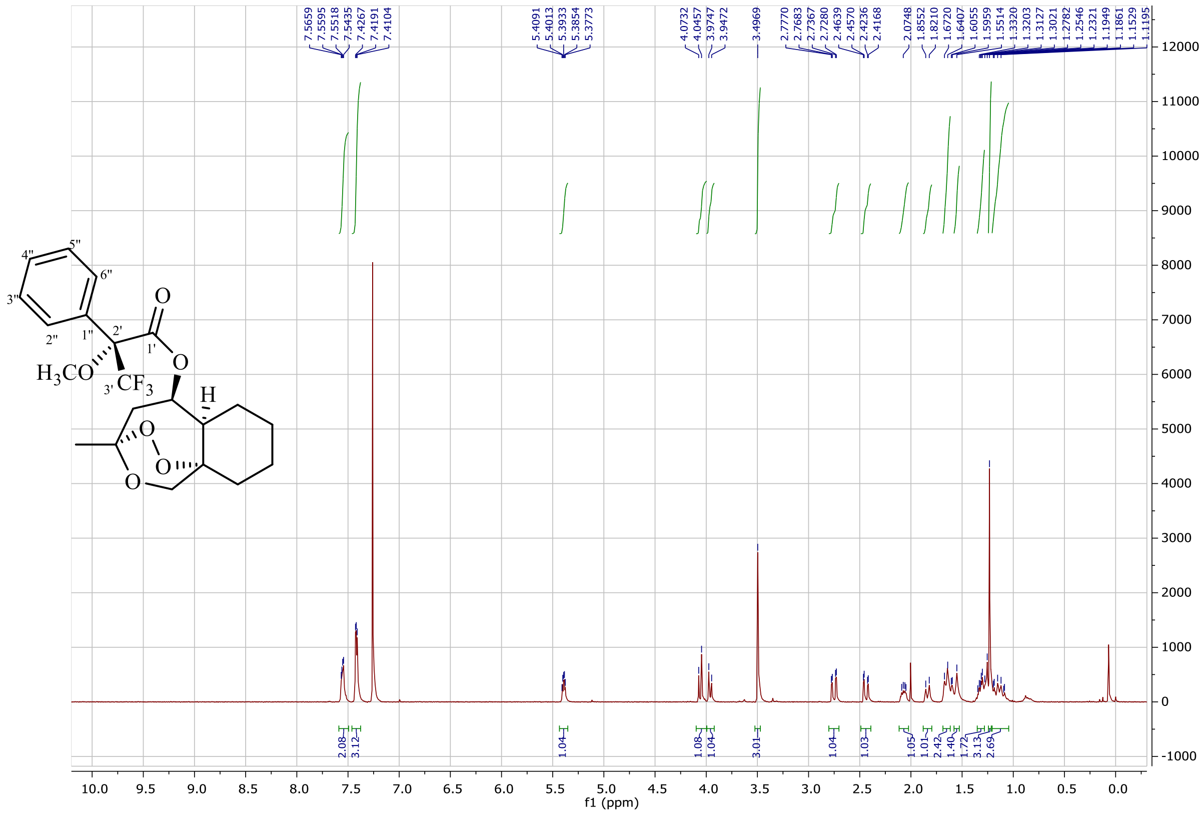

Figure S56. $^{1}$H-spectrum of the compound **19a** (CDCl3, 400 MHz).

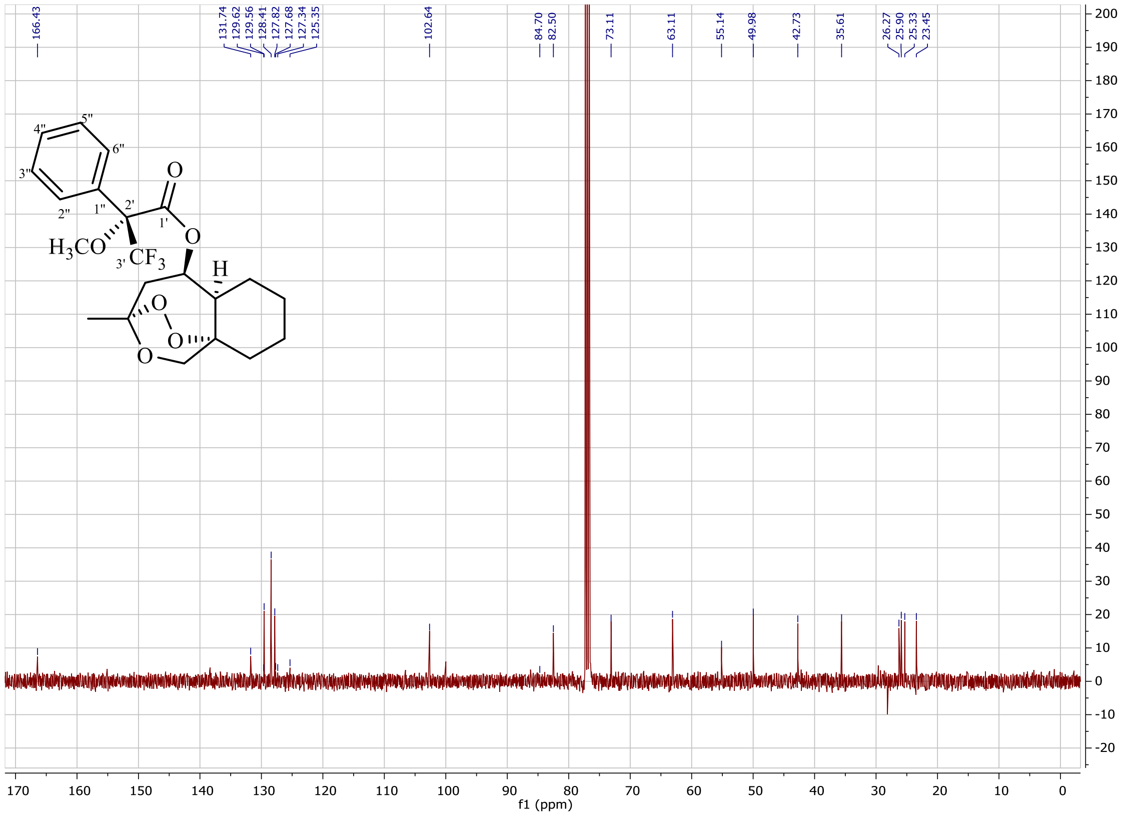


Figure S57. $^{13}$C-NMR spectrum of the compound **19a** (CDCl3, 100 MHz).

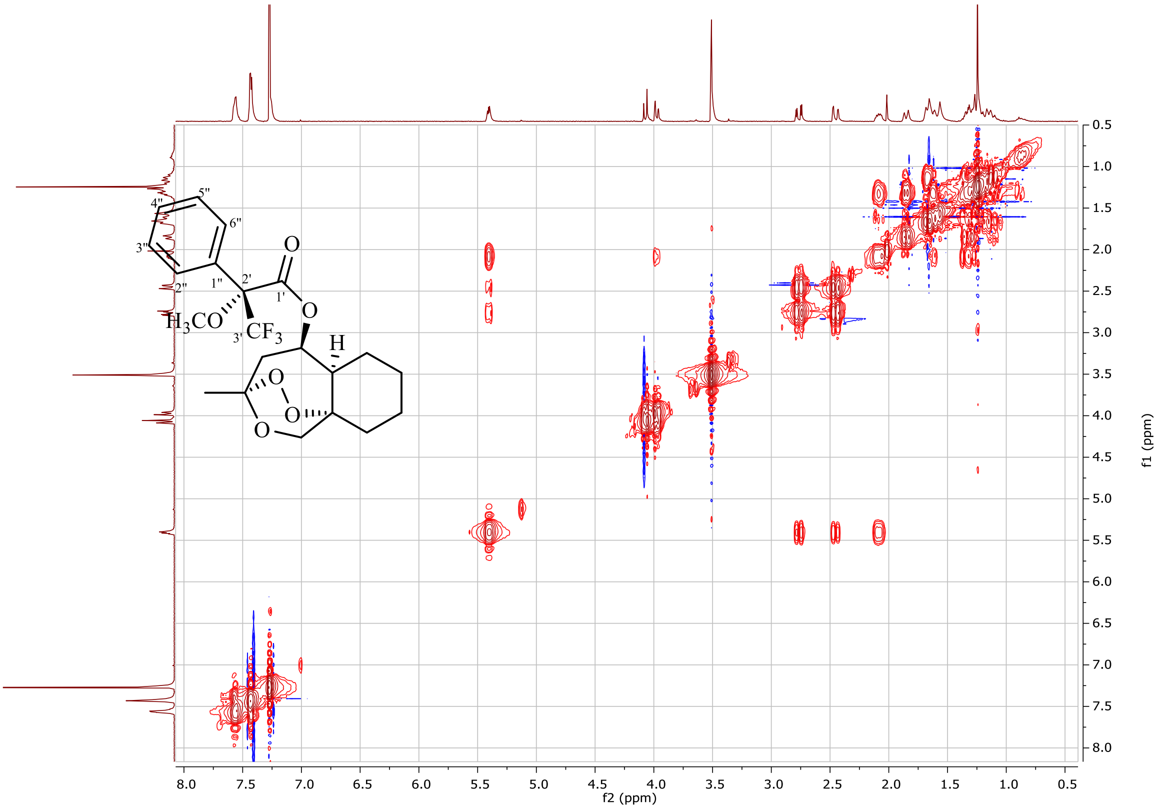


Figure S58. COSY spectrum of the compound **19a**.

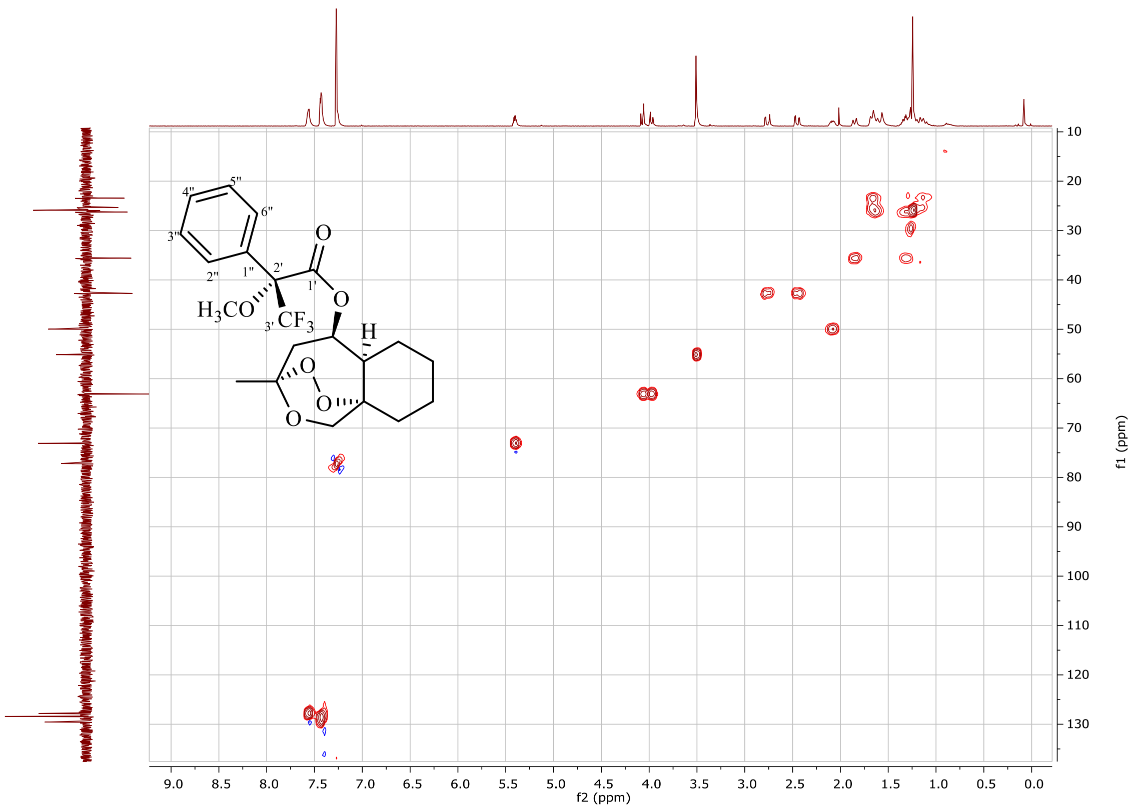


Figure S59. HSQC spectrum of the compound **19a**.

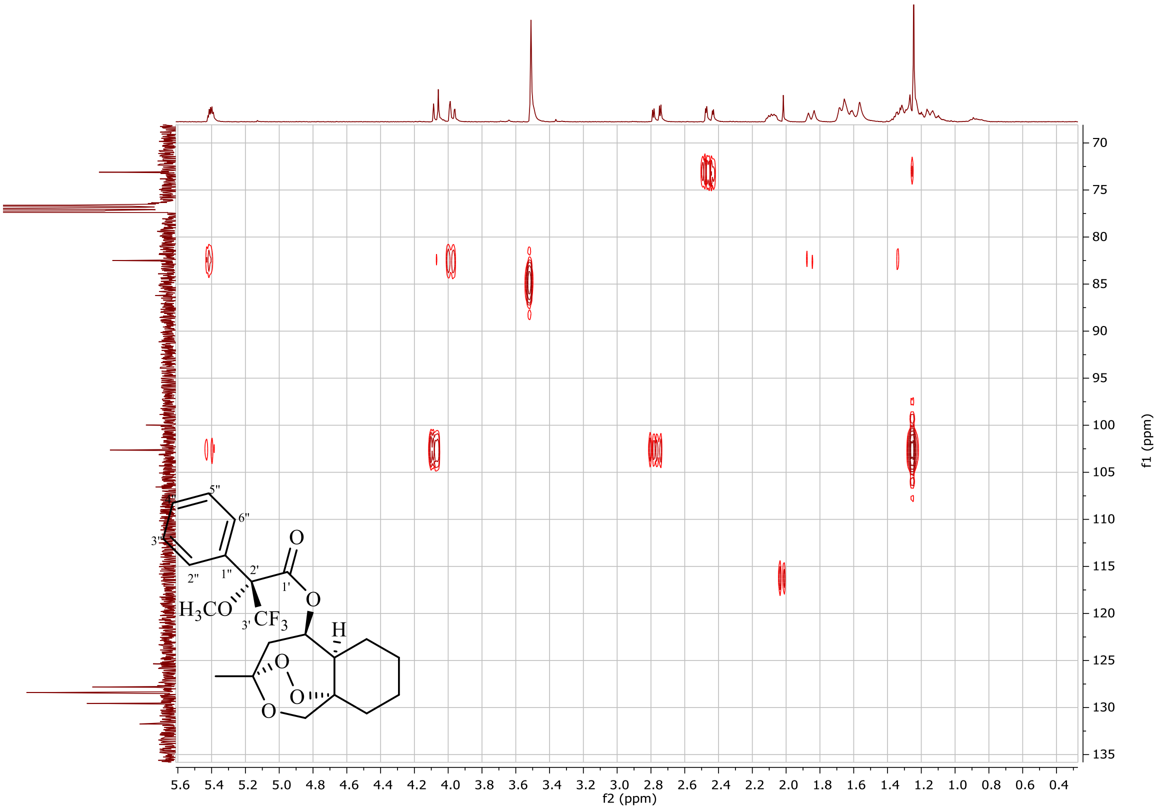


Figure S60. HMBC spectrum of the compound **19a**.

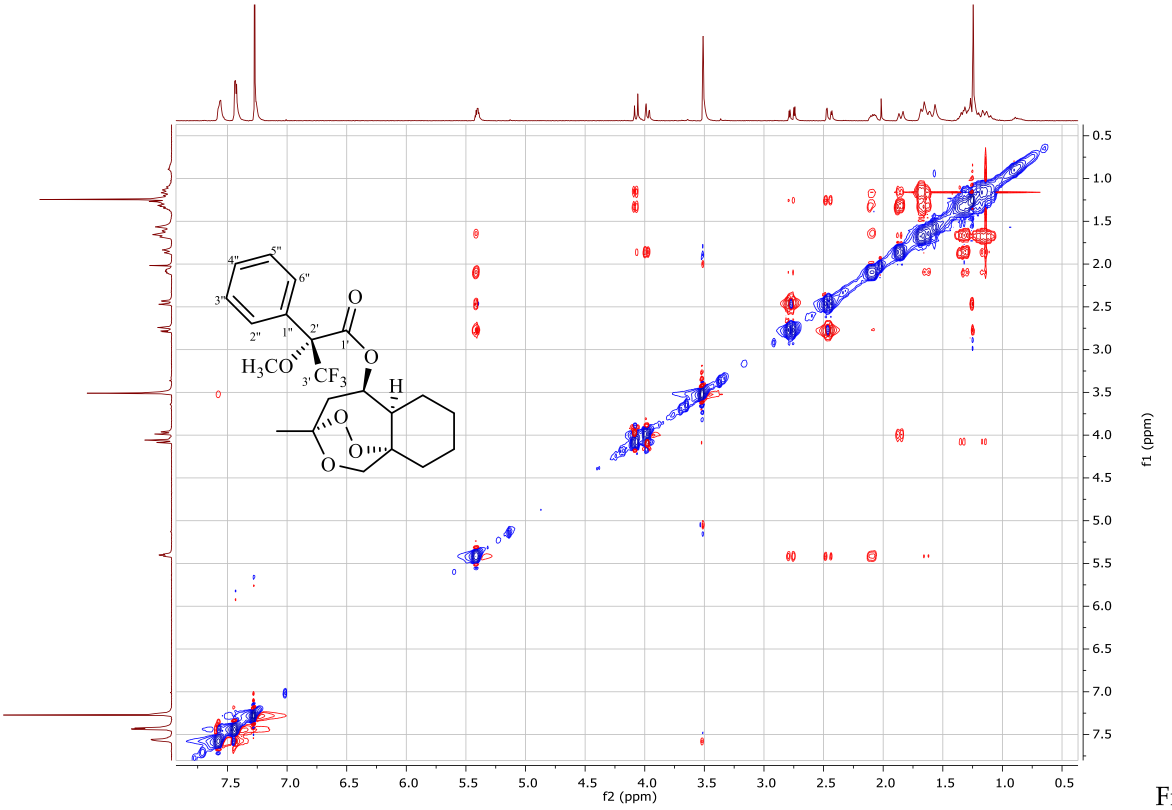


Figure S61. NOESY spectrum of the compound **19a**.

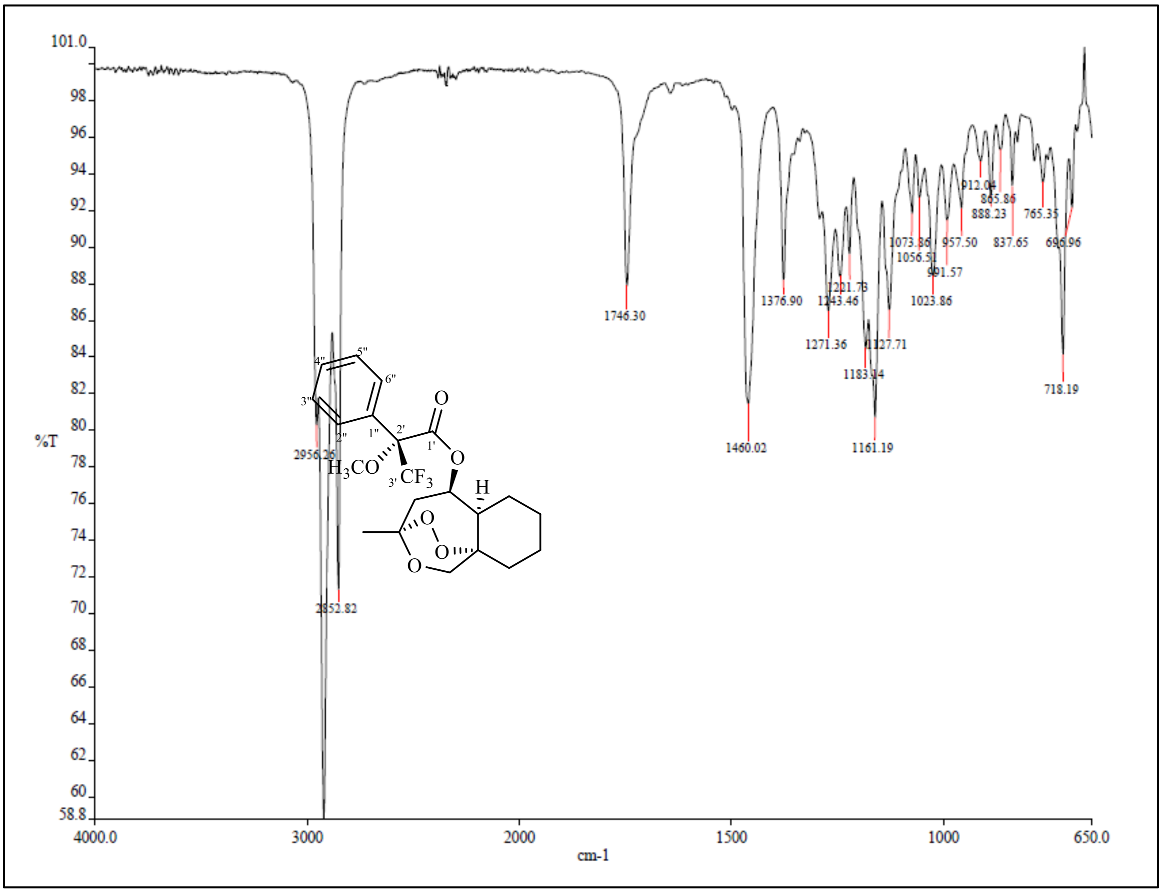


Figure S62. IR-spectrum of the compound **19a**.

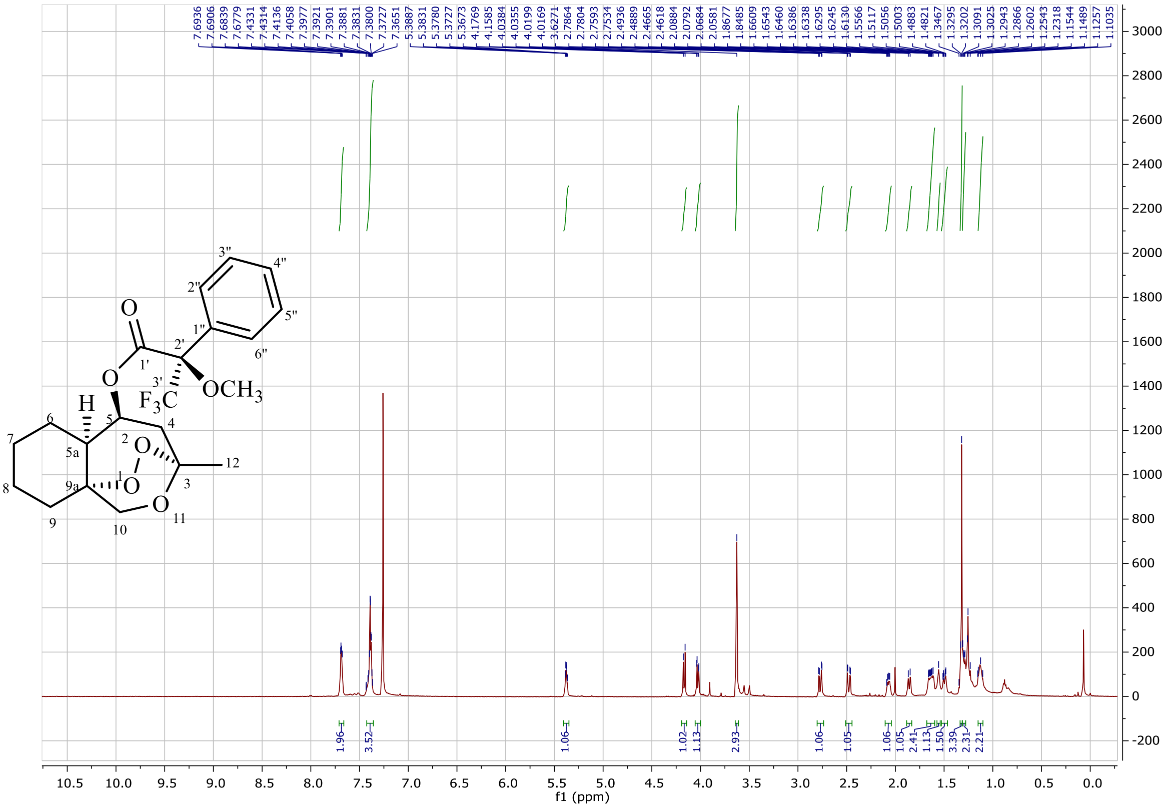

Figure S63. $^{1}$H-spectrum of the compound **19b** (CDCl3, 400 MHz).

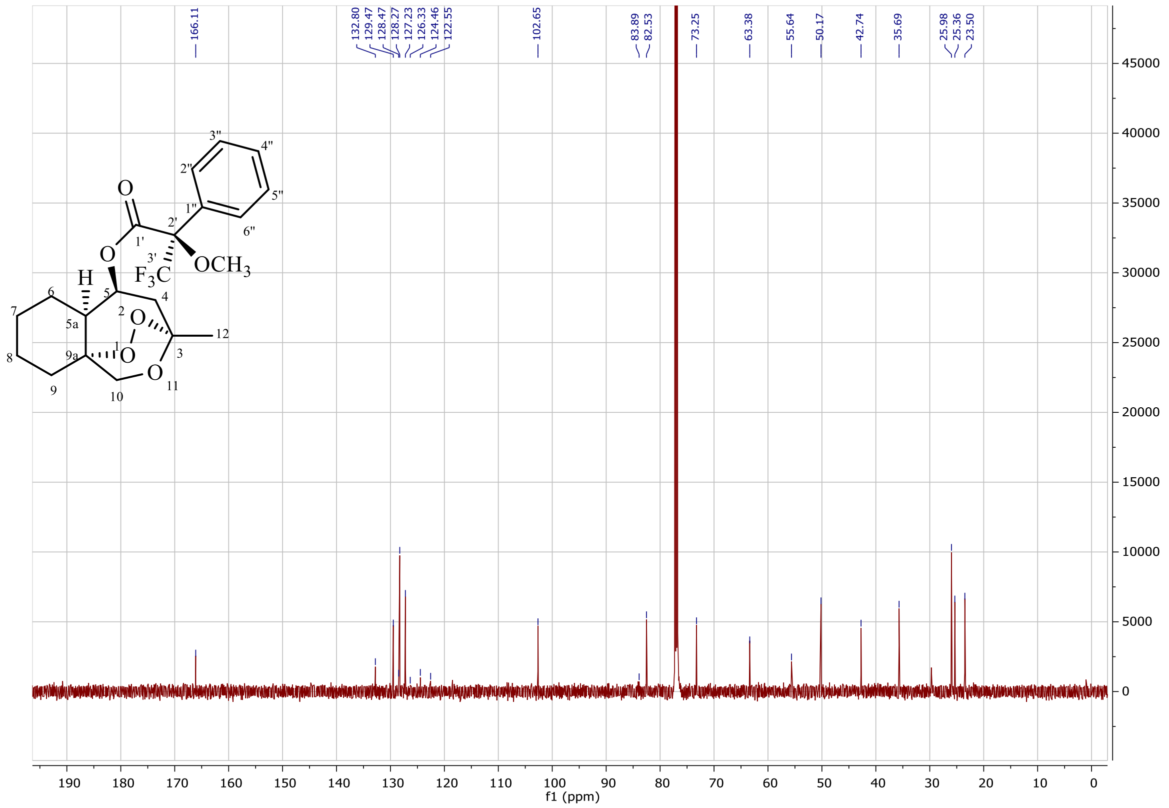


Figure S64. $^{13}$C-NMR spectrum of the compound **19b** (CDCl3, 100 MHz).

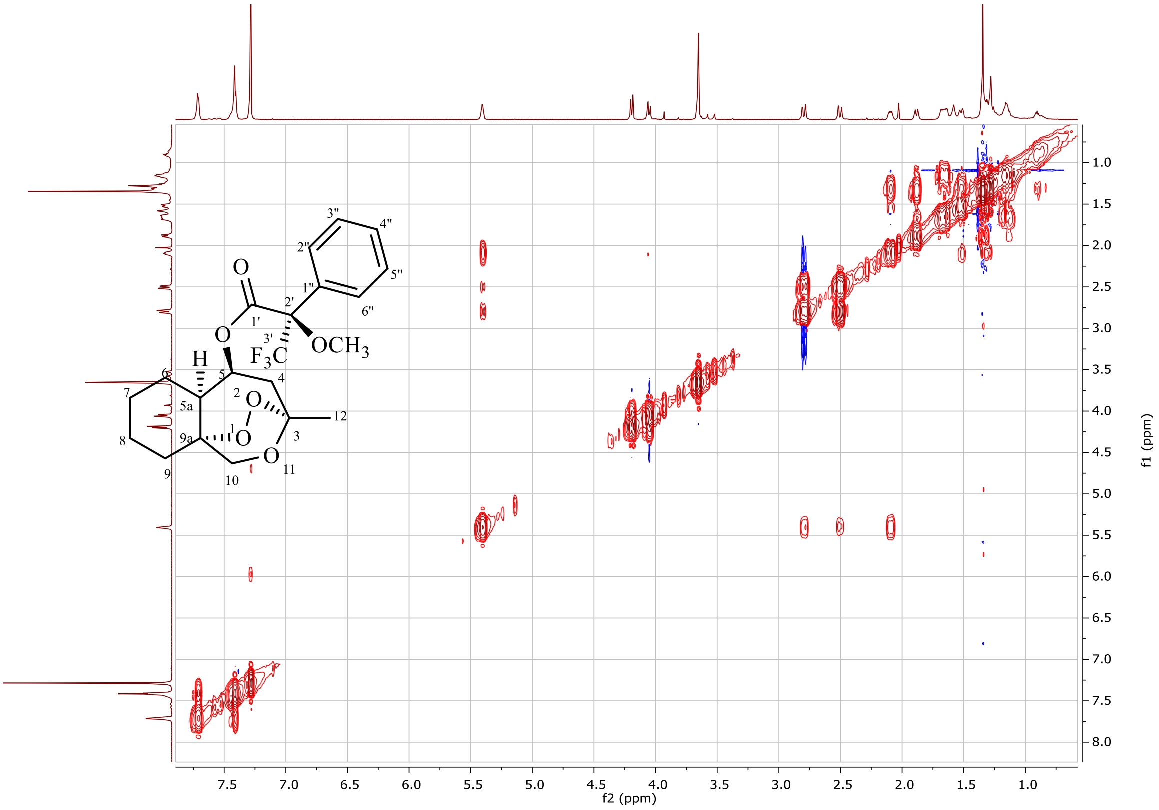

Figure S65. COSY spectrum of the compound **19b**.

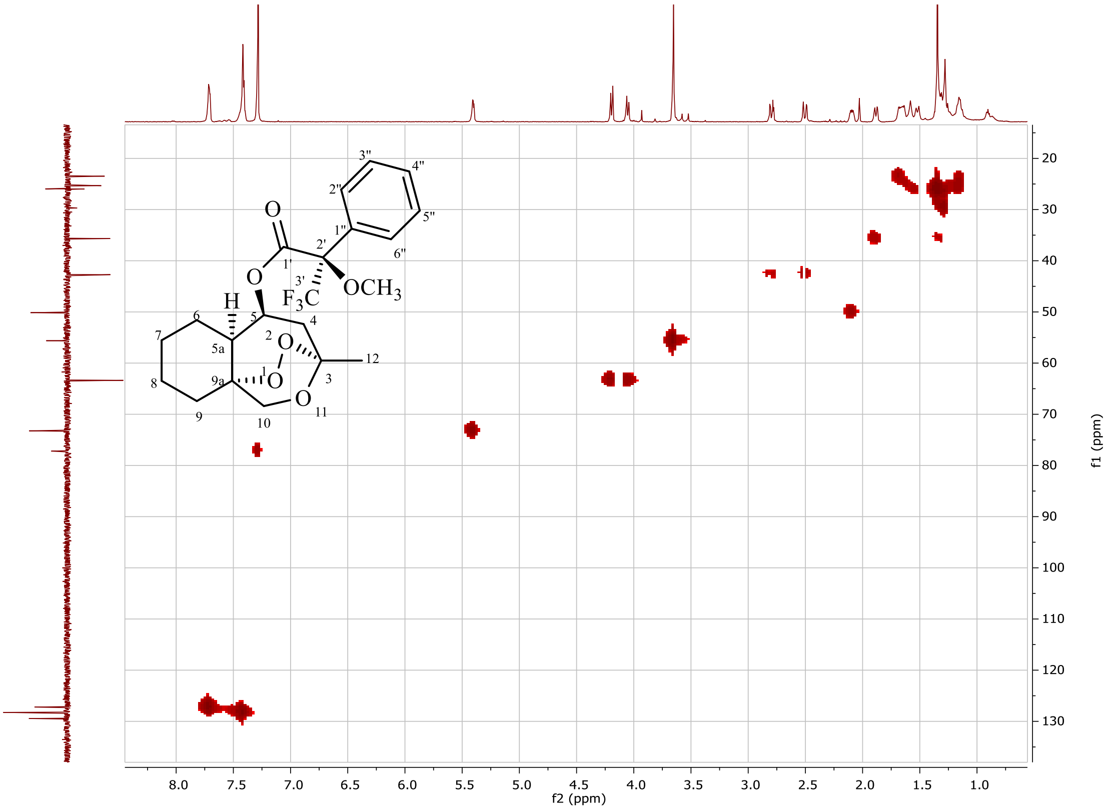

Figure S66. HSQC spectrum of the compound **19b**.

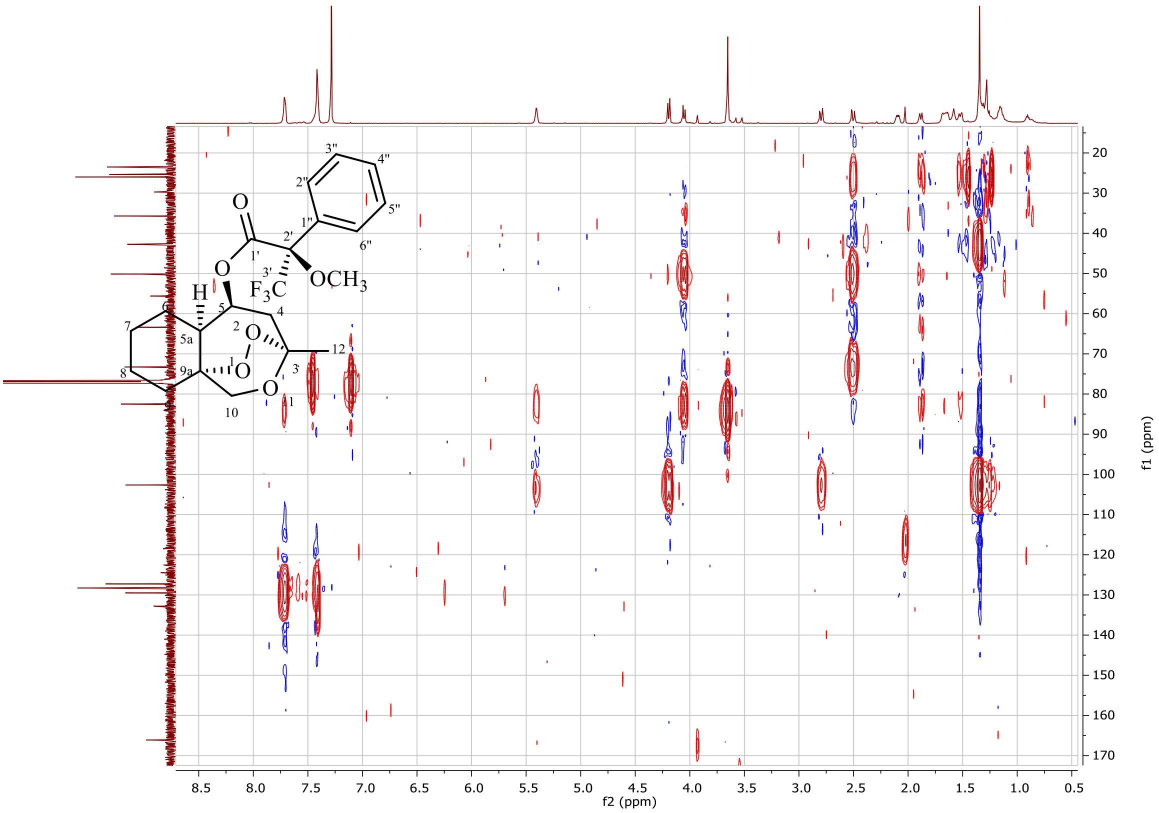


Figure S67. HMBC spectrum of the compound **19b**.

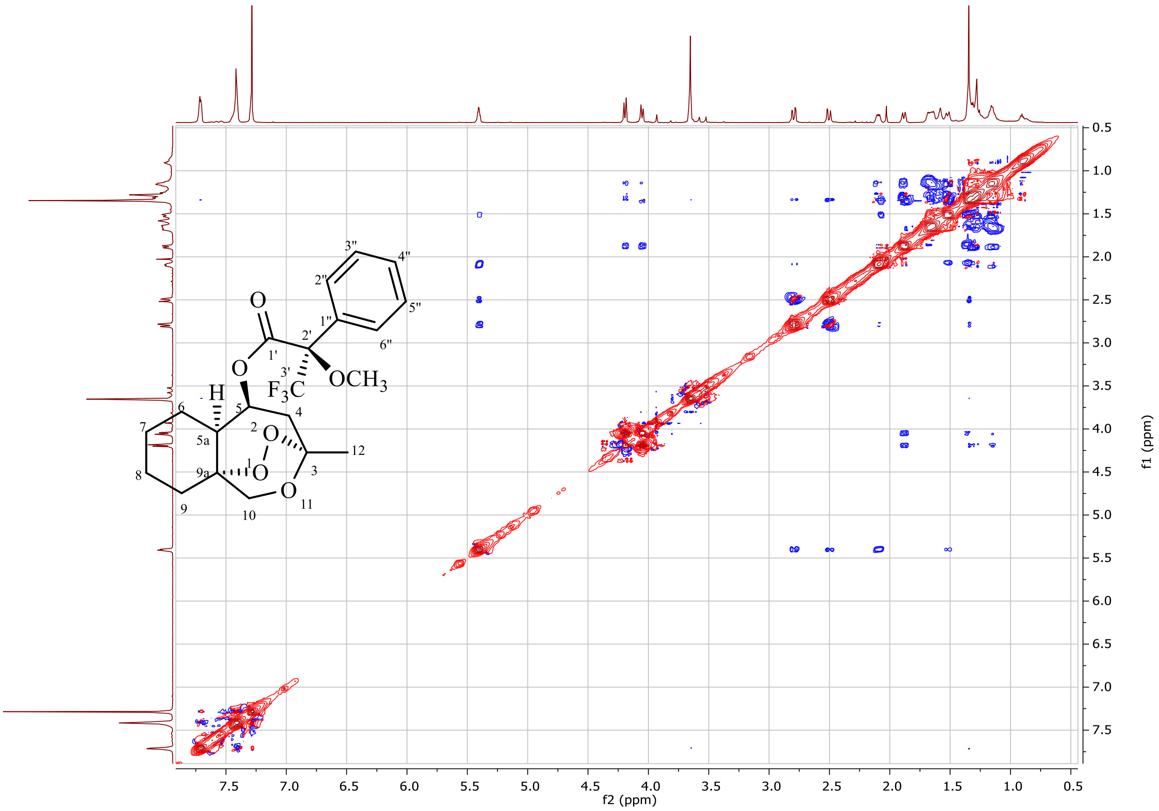


Figure S68. NOESY spectrum of the compound **19b**.

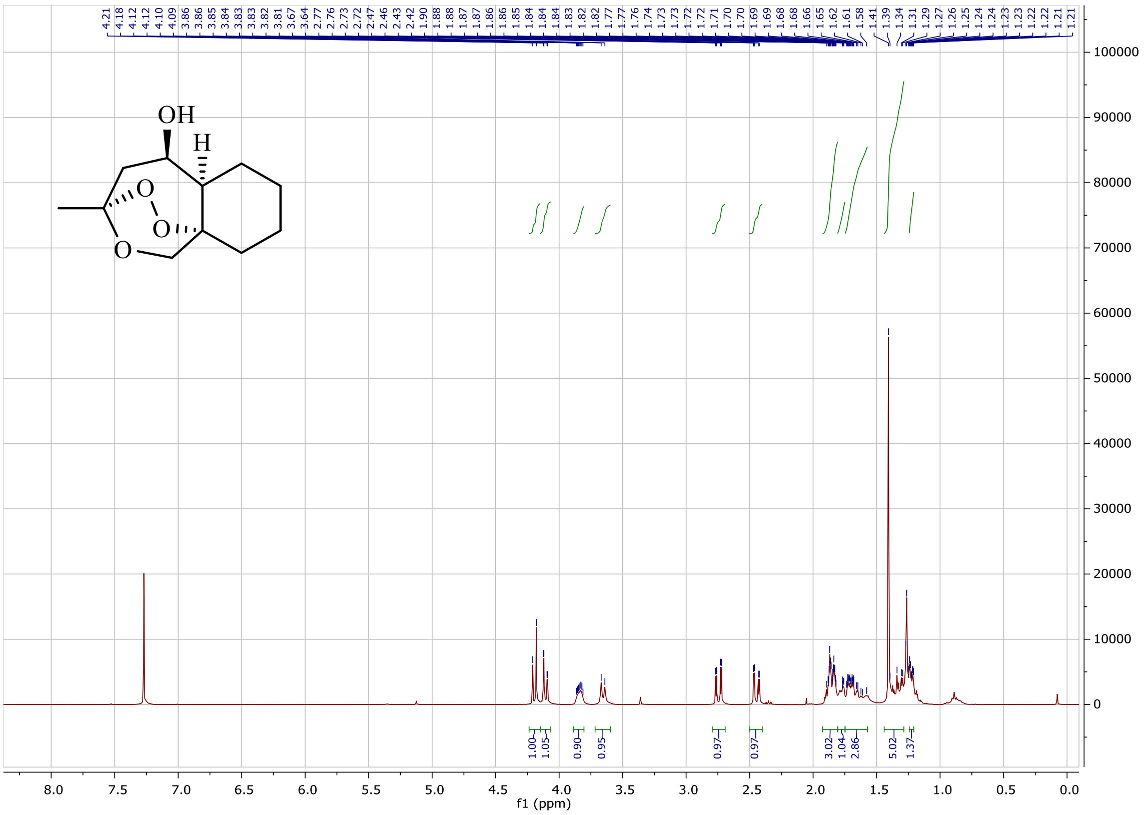

Figure S69. $^{1}$H-spectrum of the compound **2a** (CDCl3, 400 MHz).

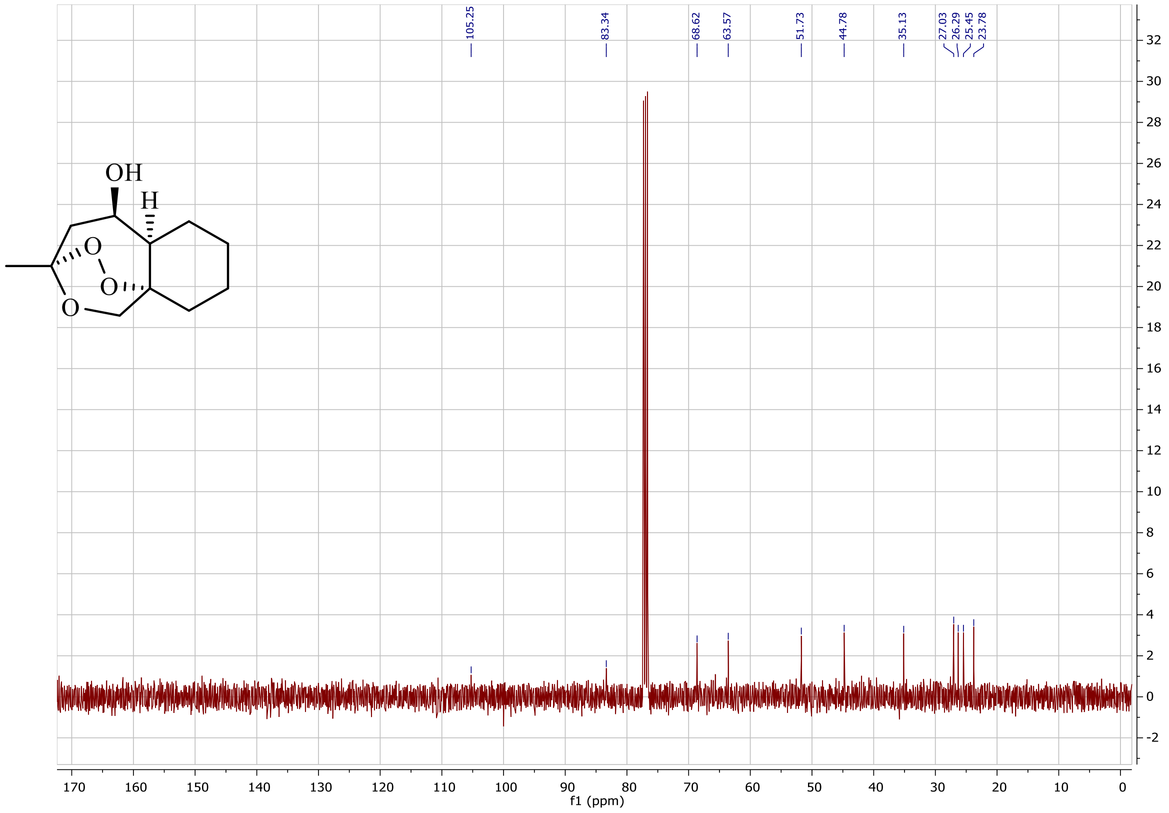


Figure S70. $^{13}$C-NMR spectrum of the compound **2a** (CDCl3, 100 MHz).

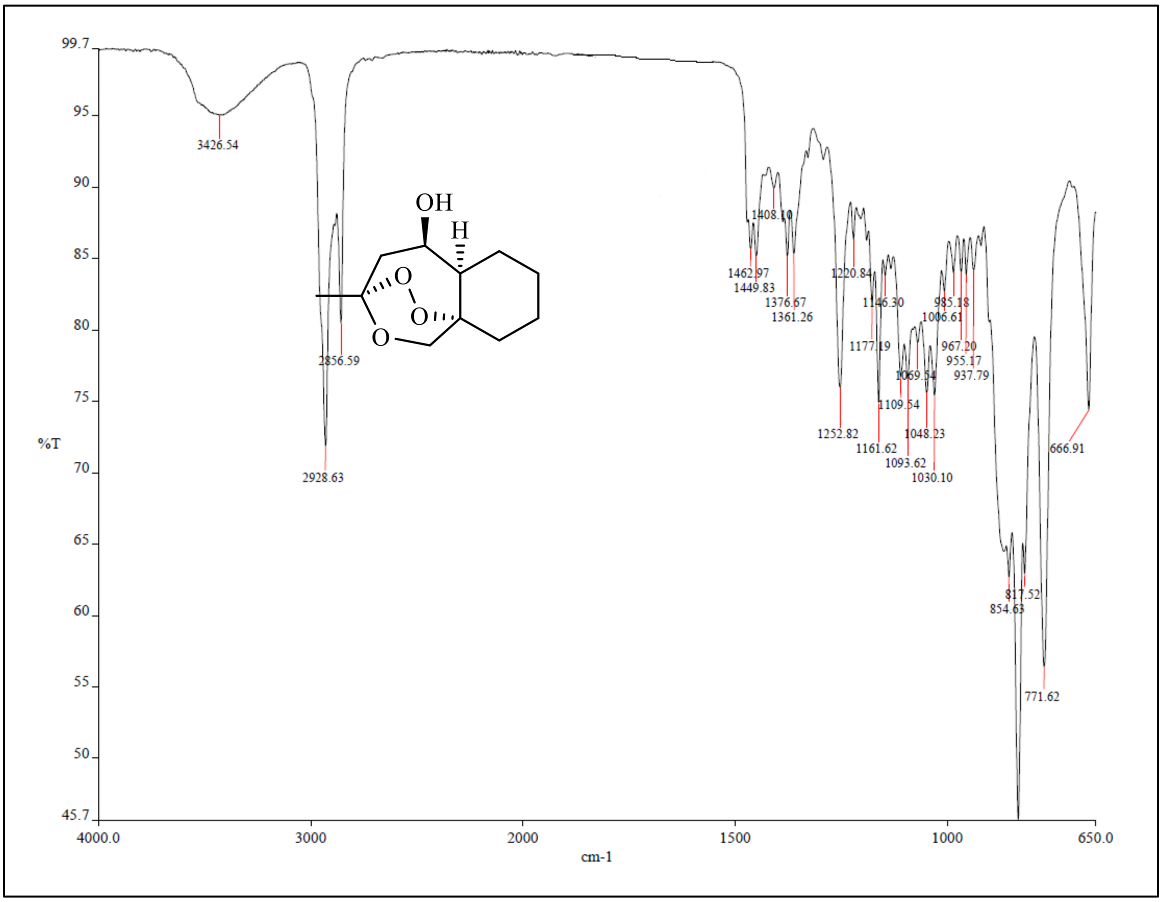


Figure S71. IR-spectrum of the compound **2a**.

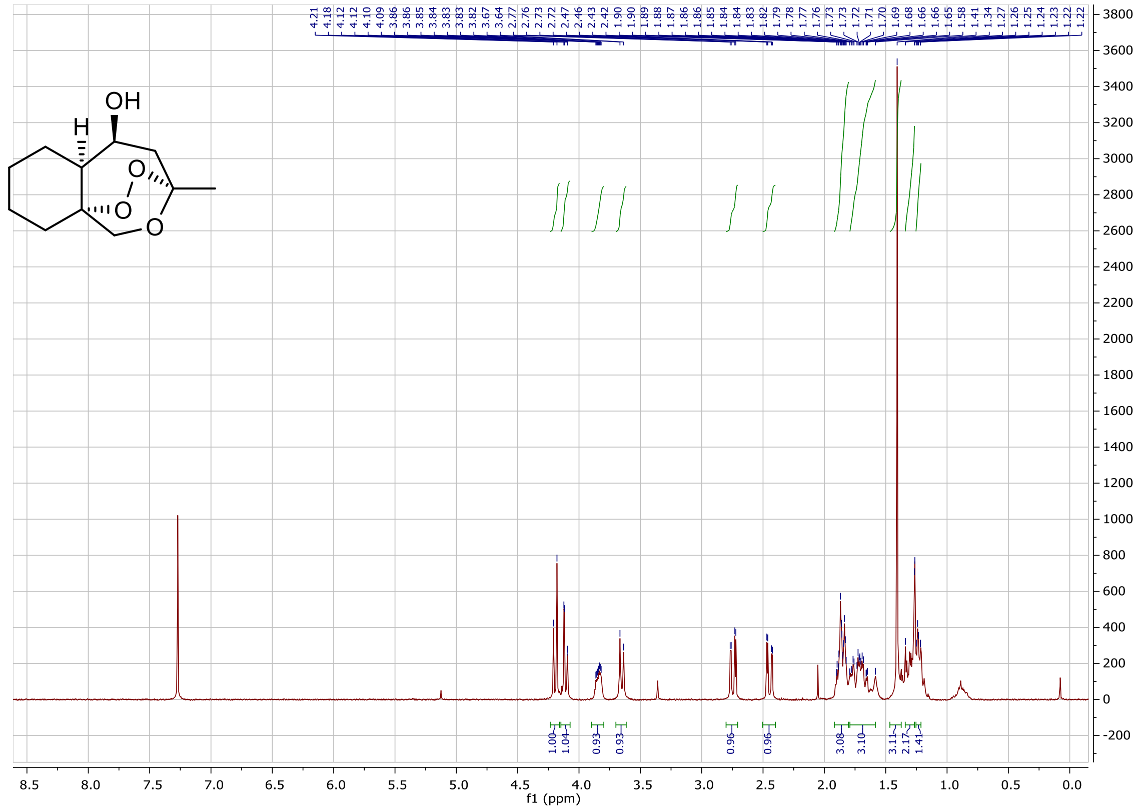

Figure S72. $^{1}$H-spectrum of the compound **2b** (CDCl3, 400 MHz).

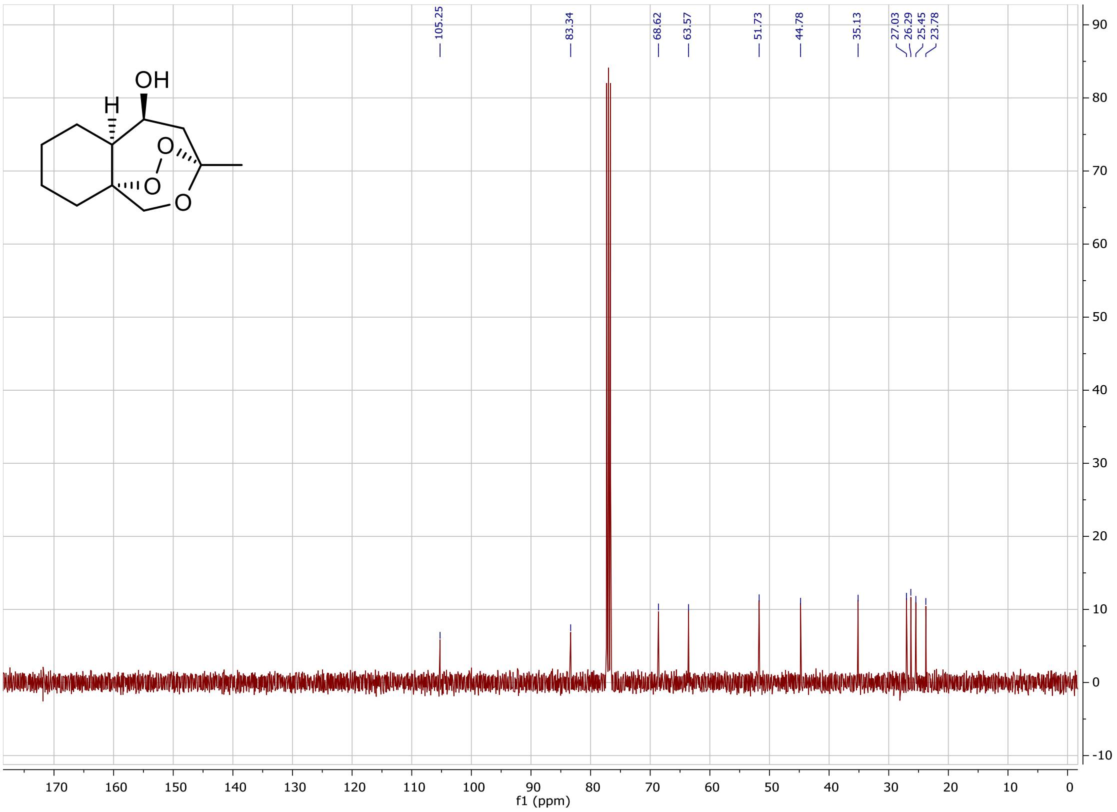


Figure S73. $^{13}$C-NMR spectrum of the compound **2b** (CDCl3, 100 MHz).

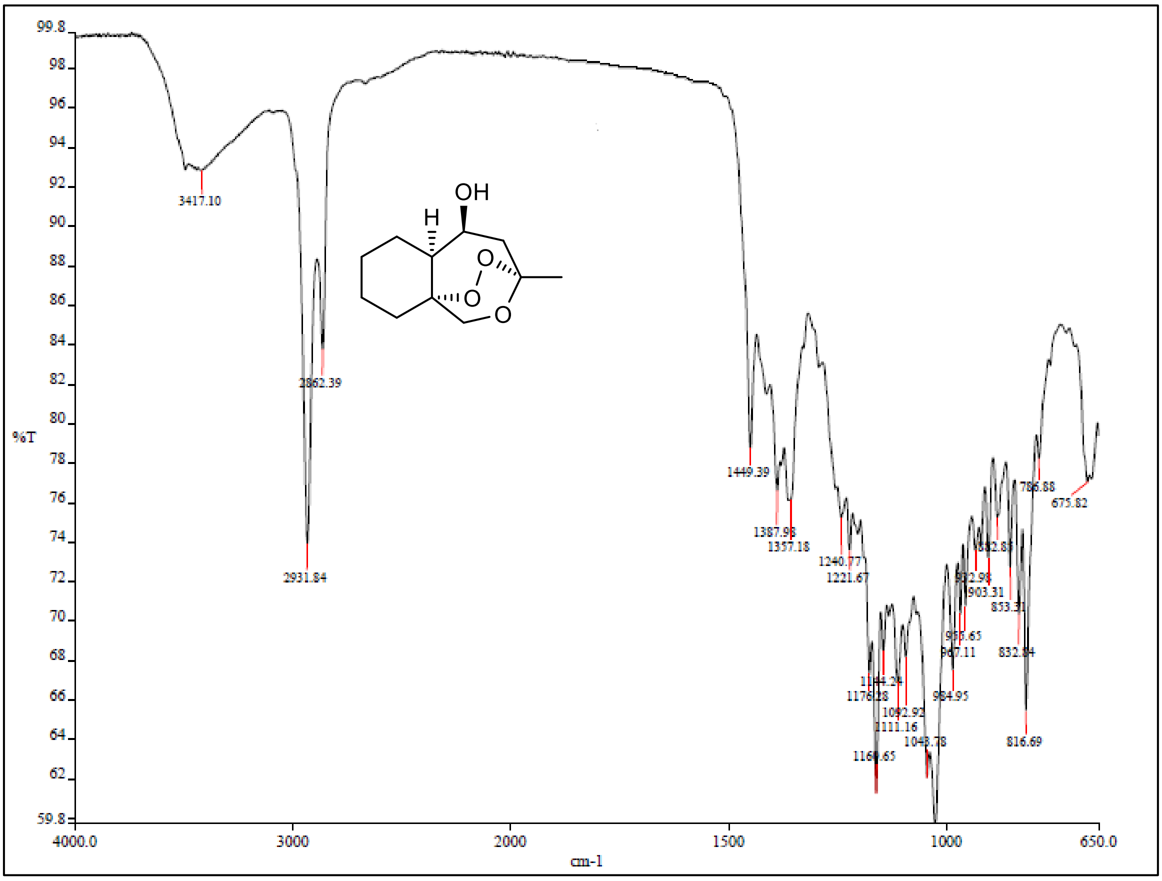


Figure S74. IR-spectrum of the compound **2b**.

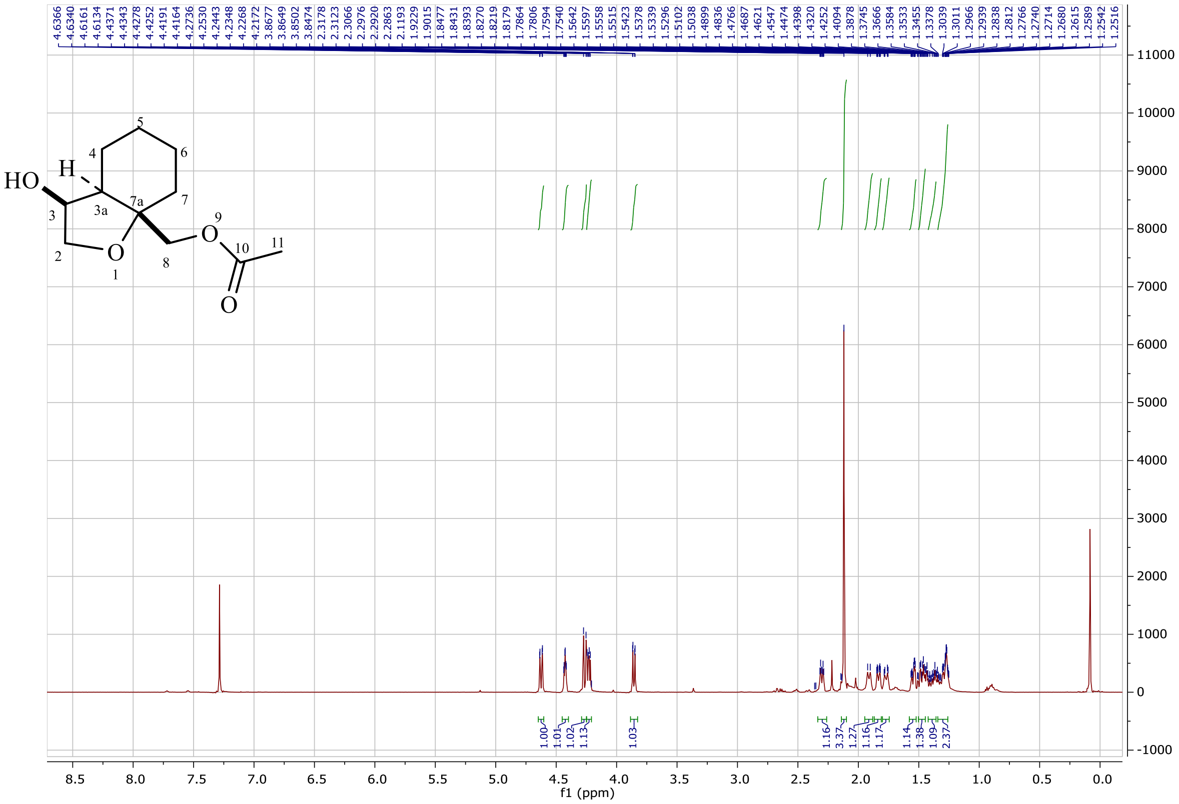

Figure S75. $^{1}$H-spectrum of the compound **22** (CDCl3, 400 MHz).

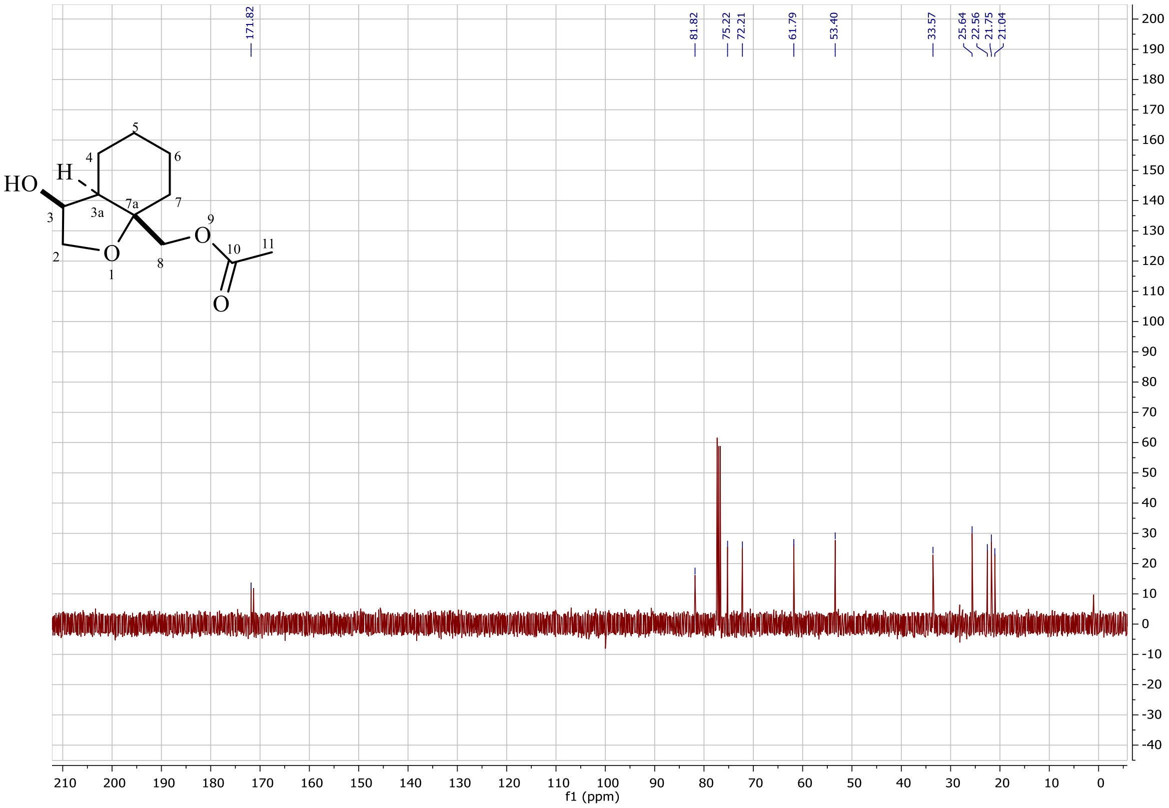


Figure S76. $^{13}$C-NMR spectrum of the compound **22** (CDCl3, 100 MHz).

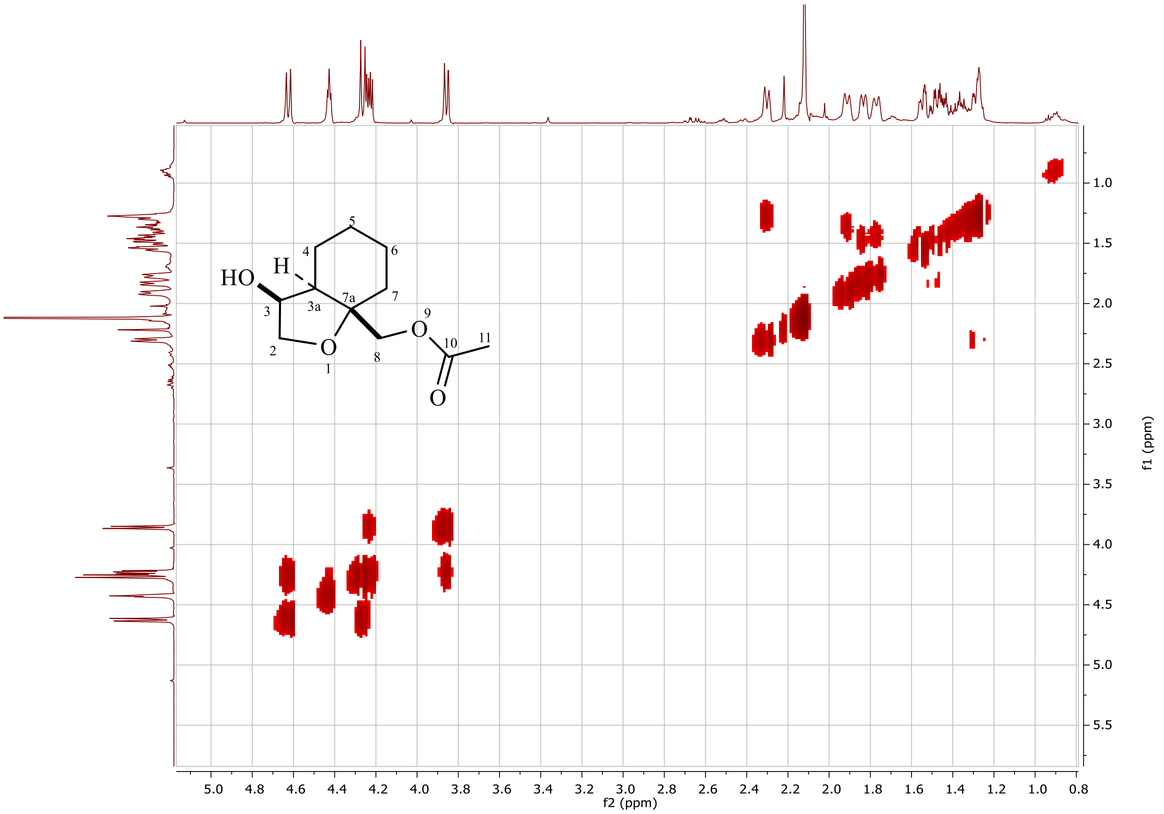


Figure S77. COSY spectrum of the compound **22**.

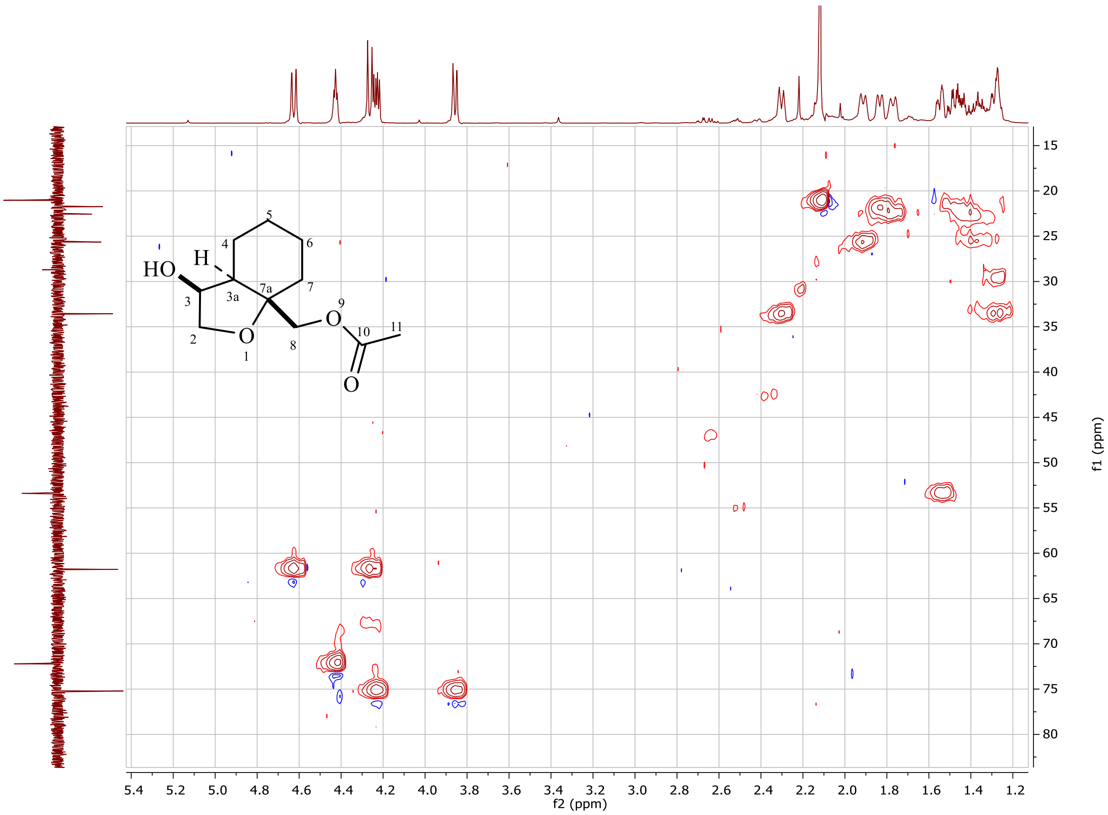


Figure S78. HSQC spectrum of the compound **22**.

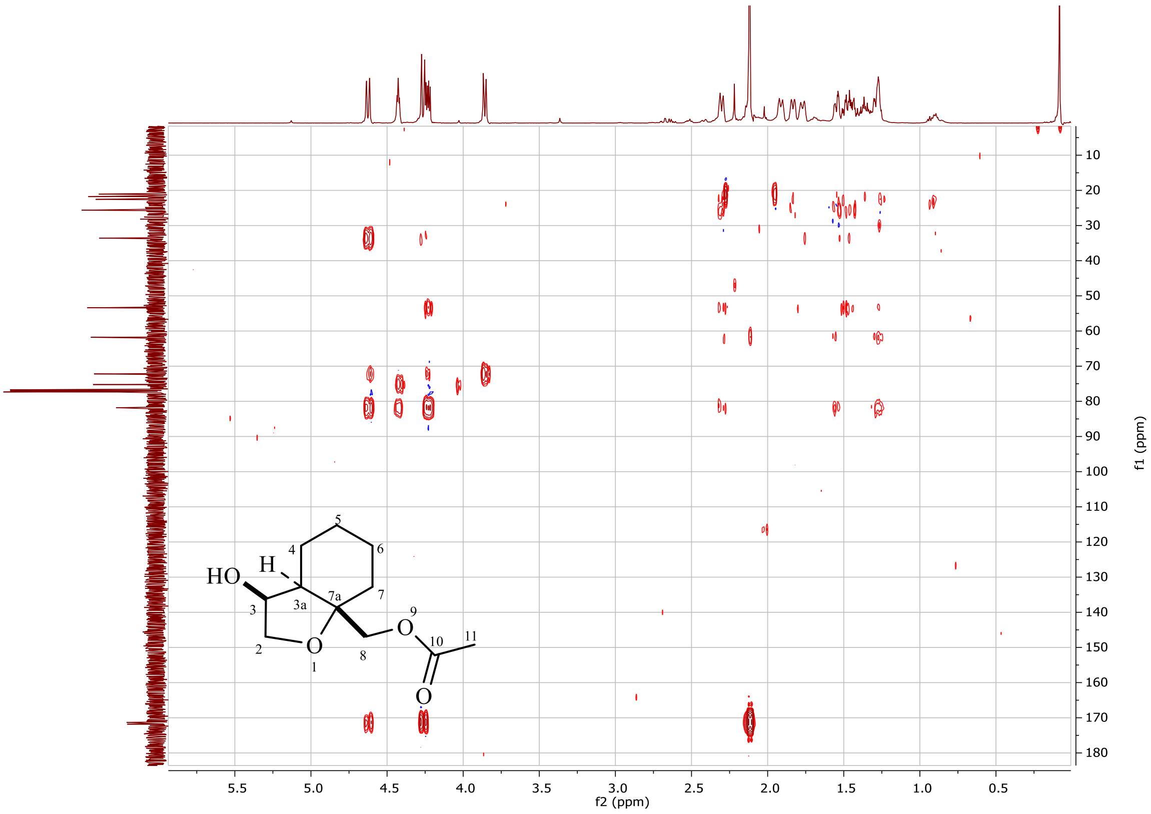


Figure S79. HMBC spectrum of the compound **22**.

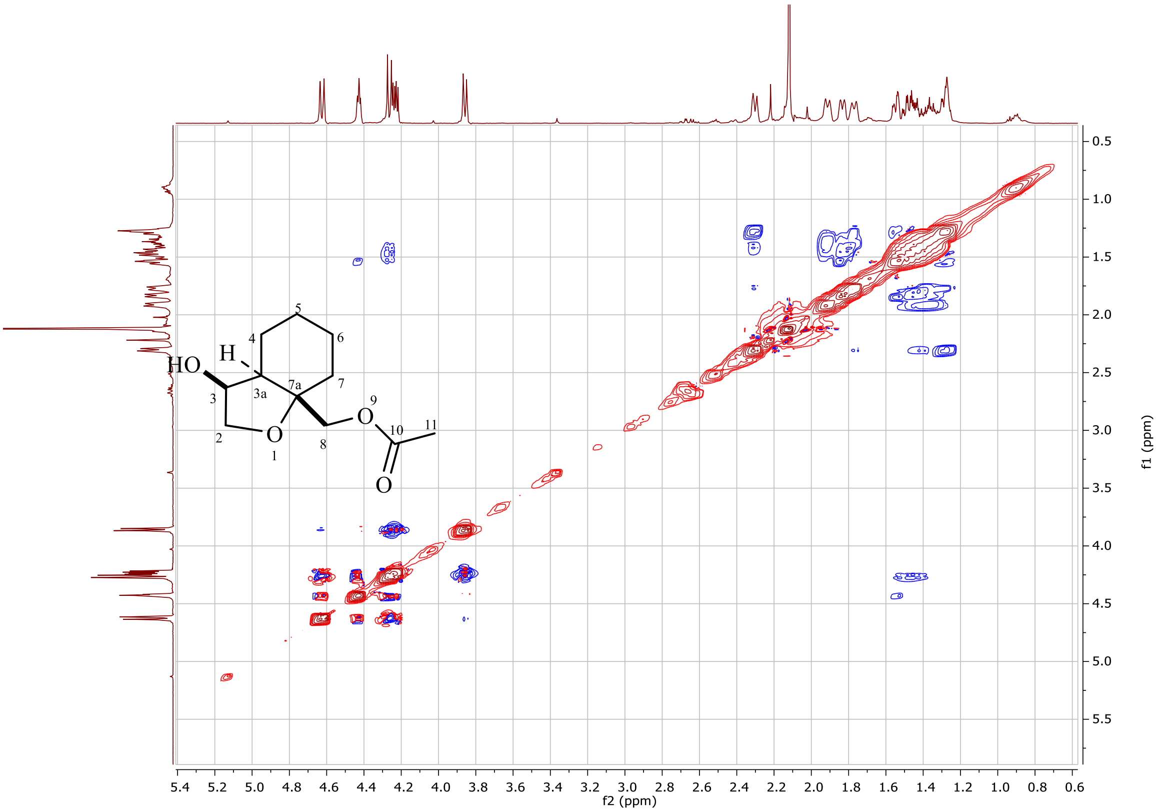


Figure S80. NOESY spectrum of the compound **22**.

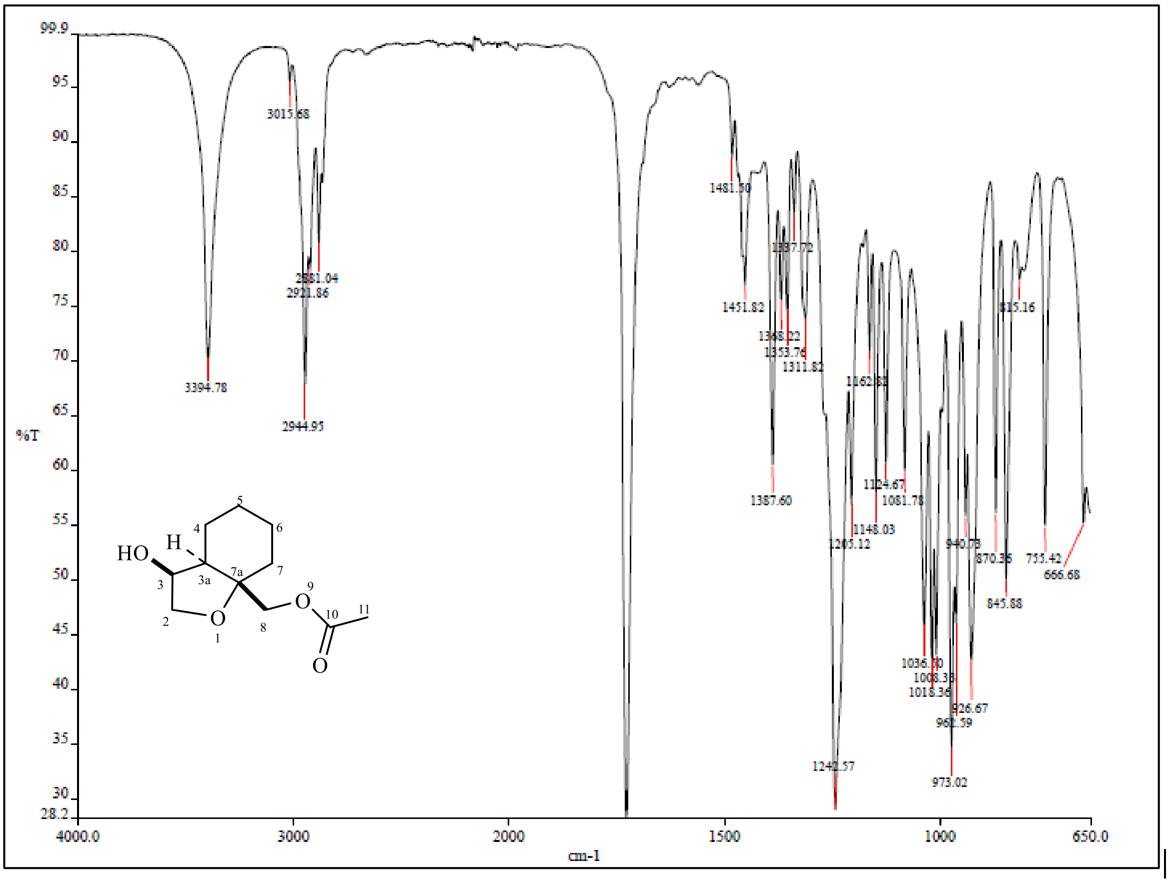


Figure S81. IR-spectrum of the compound **22**.

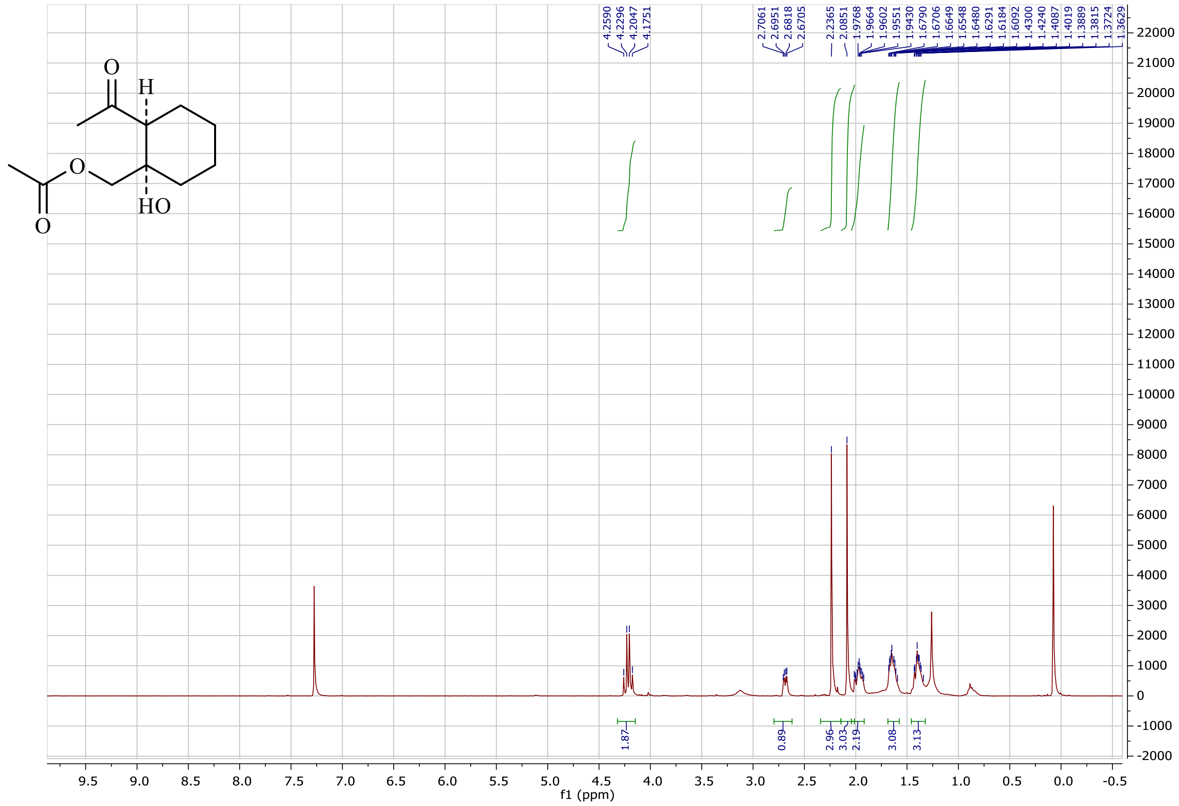

Figure S82. $^{1}$H-spectrum of the compound **29** (CDCl3, 400 MHz).

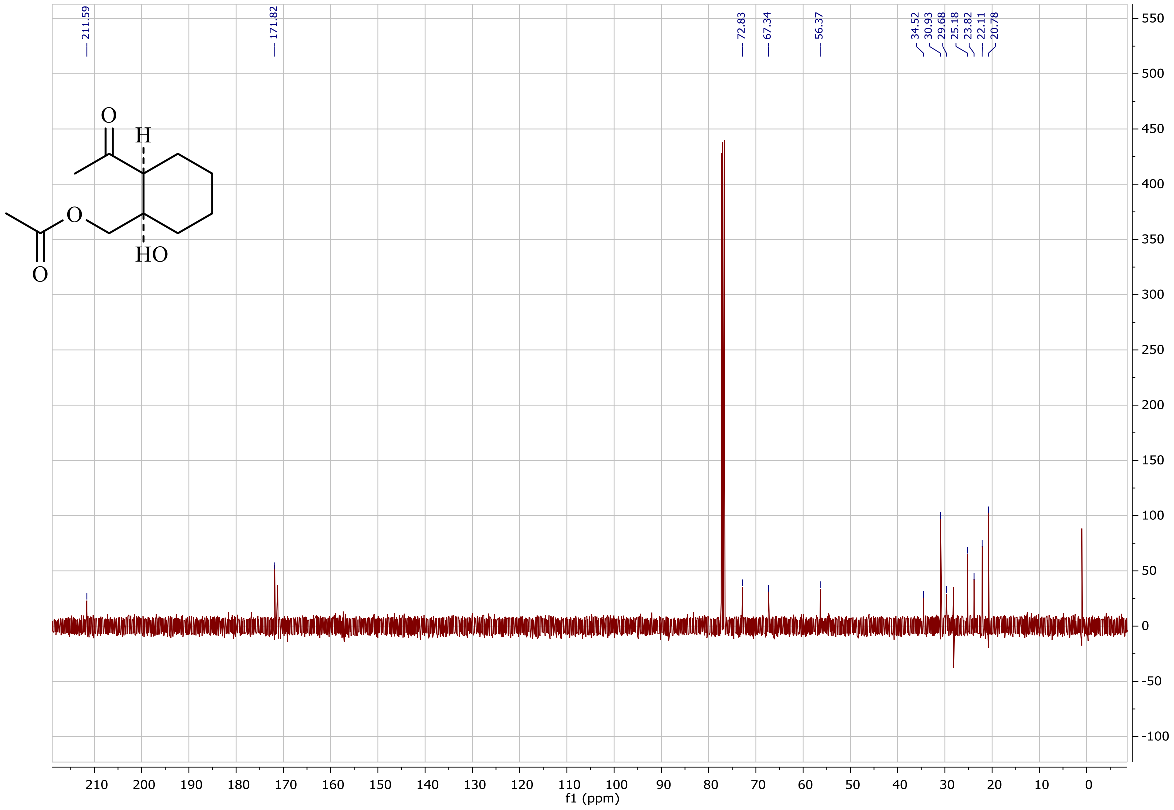


Figure S83. $^{13}$C-NMR spectrum of the compound **29** (CDCl3, 100 MHz).

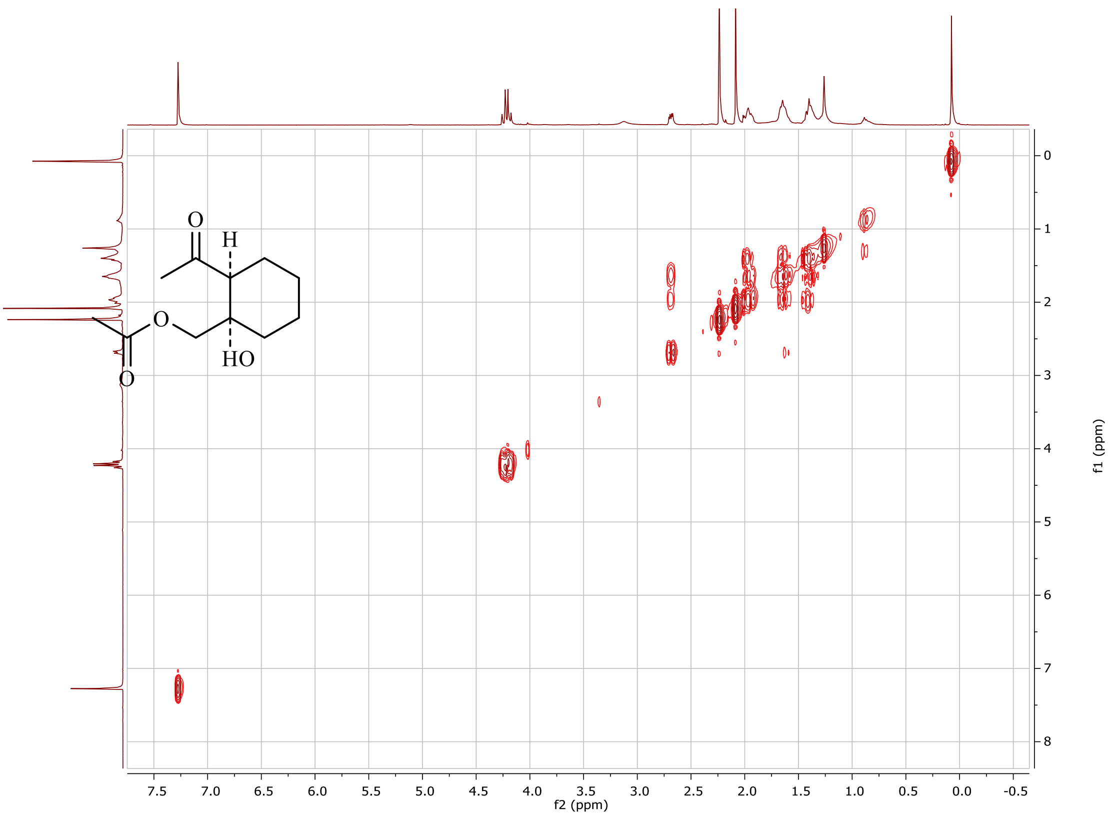


Figure S84. COSY spectrum of the compound **29**.

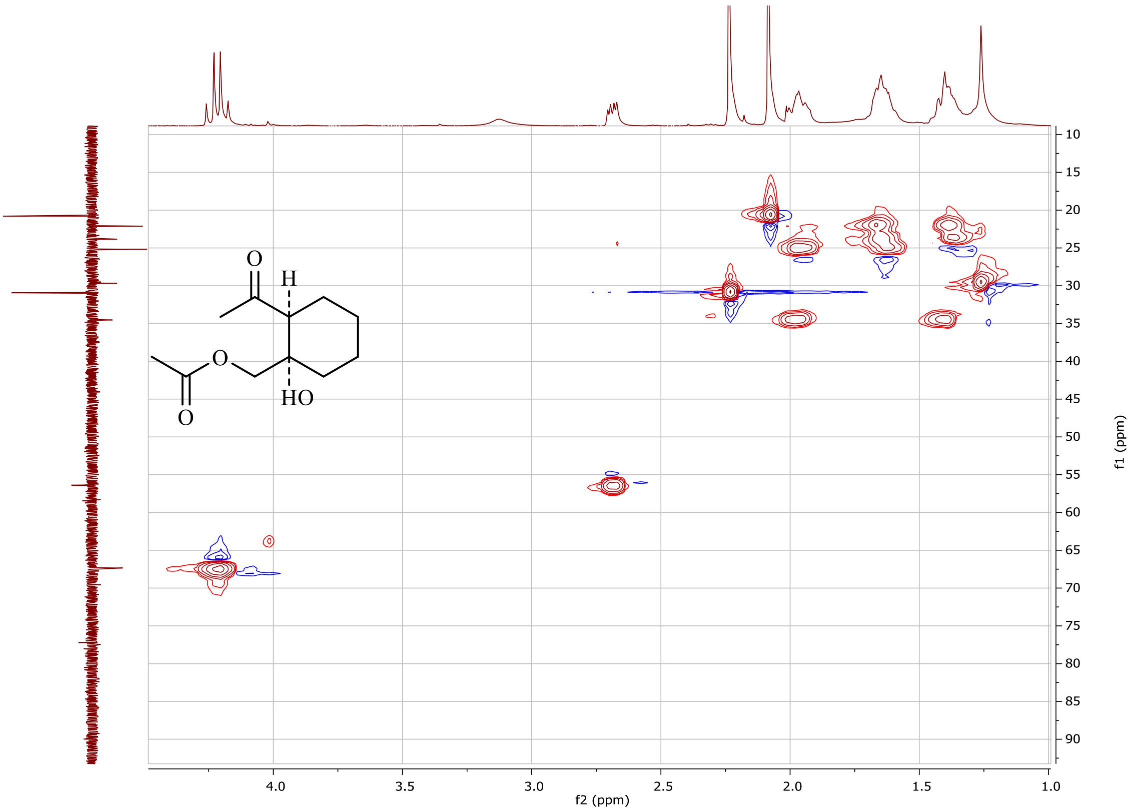


Figure S85. HSQC spectrum of the compound **29**.

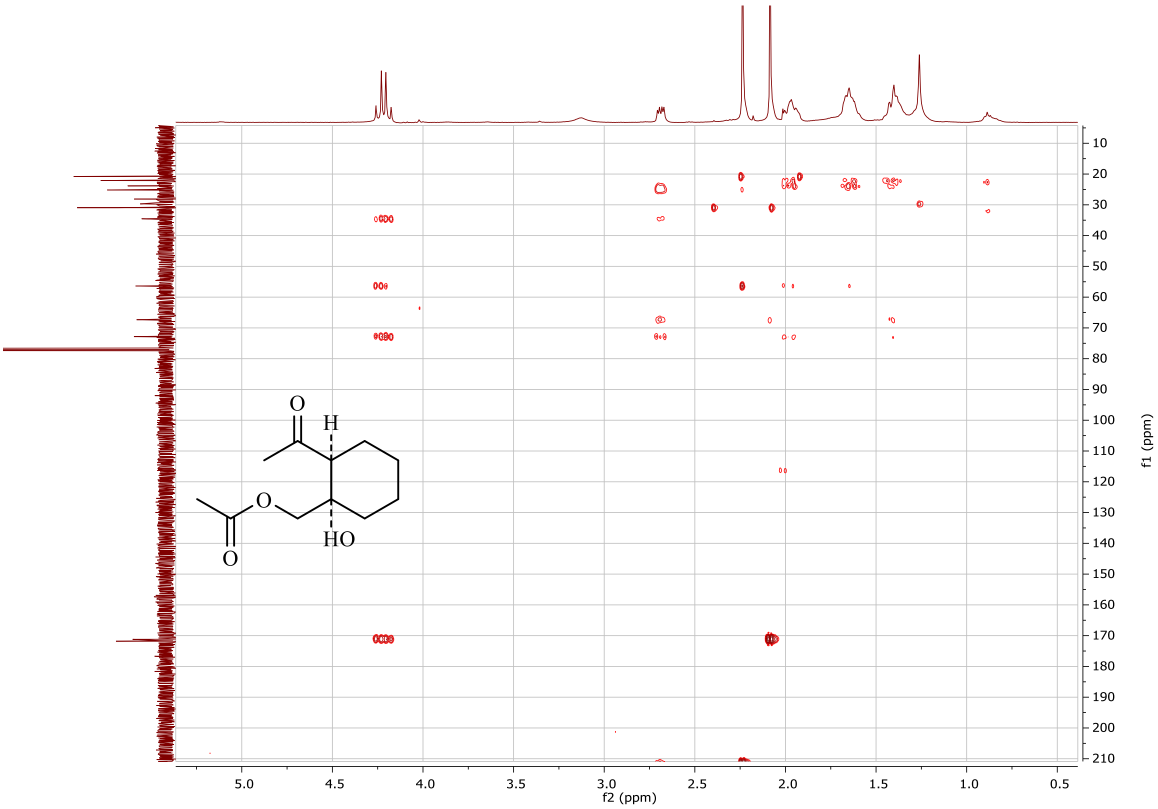


Figure S86. HMBC spectrum of the compound **29**.

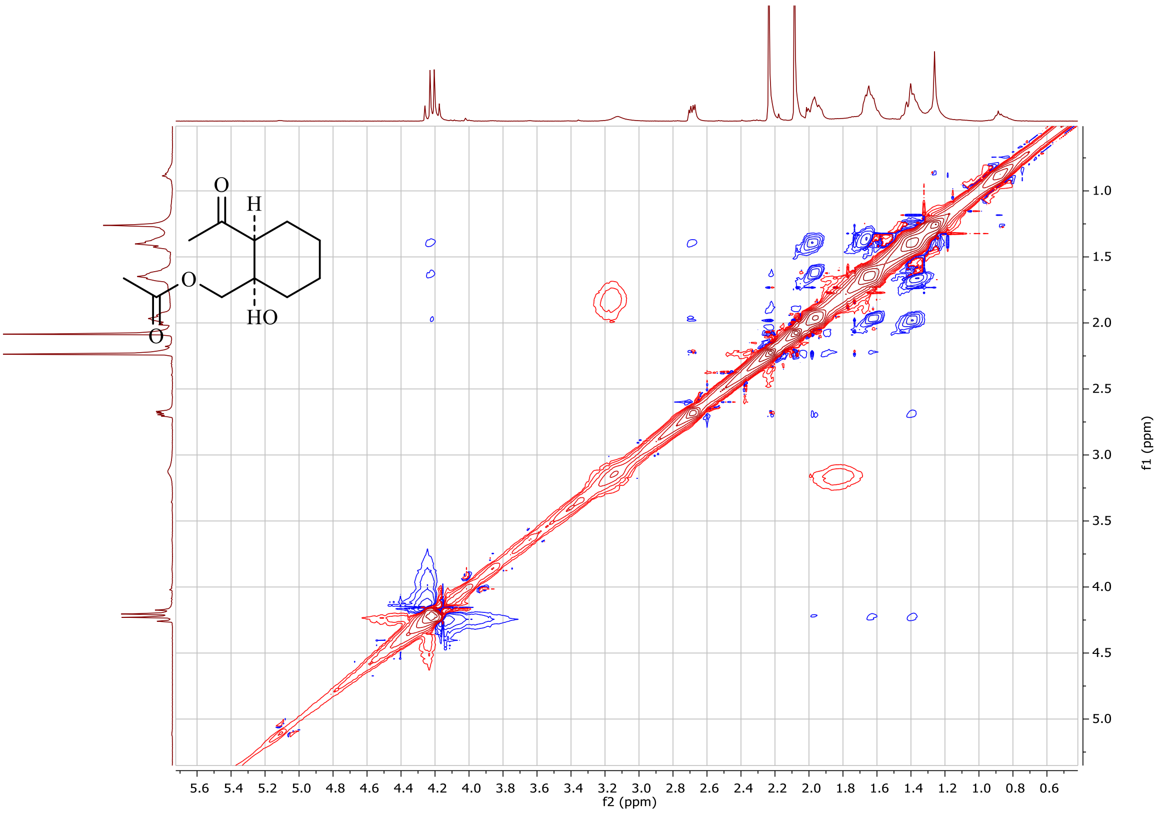


Figure S87. NOESY spectrum of the compound **29**.
